# Vaginal herb use and *Chlamydia trachomatis* infection: cross-sectional study among women of various ethnic groups in Suriname

**DOI:** 10.1136/bmjopen-2018-025417

**Published:** 2019-05-16

**Authors:** Jannie J Van der Helm, Maarten Franciscus Schim van der Loeff, Esther de Vries, Charlotte van der Veer, Antoon W Grünberg, Dennis Mans, Henry J C de Vries

**Affiliations:** 1 Department of Infectious Diseases, Public Health Service Amsterdam, Amsterdam, The Netherlands; 2 Department of Public Health, Ministry of Health, Paramaribo, Suriname; 3 Department of Pharmacology, Faculty of Medical Sciences, Anton de Kom University of Suriname, Paramaribo, Suriname; 4 Department of Dermatology, Amsterdam UMC location Academic Medical Centre, Amsterdam Institute for Infection and Immunity (AI&II), Amsterdam, The Netherlands

**Keywords:** herb therapy, vaginal douching, vaginal, ethnicity, Suriname

## Abstract

**Objective:**

Vaginal steam baths with herb leaves (herb use) is practised by some Surinamese women. We assessed herb use among women from the five most prevalent ethnic groups, and if herb use is associated with *Chlamydia trachomatis* infection.

**Setting:**

Participants were recruited at a sexually transmitted infection (STI) clinic and a family planning clinic (FP) in Paramaribo, Suriname.

**Participants:**

1040 women were included subsequently, comprising the following ethnic groups: Creole (26.7%), Hindustani (24.6%), Javanese (15.7%), Maroon (13.3%) and mixed descent (19.7%).

**Methods:**

Nurses collected a questionnaire and vaginal swabs for nucleic acid amplification *C. trachomatis* testing.

**Primary outcomes:**

Determinants of vaginal herb use and *C. trachomatis* infection via univariable and multivariable logistic regression.

**Results:**

Herb use was most common among Maroon (68.8%) and Creole women (25.2%). In multivariable analysis including only Maroon and Creole women, determinants significantly associated with vaginal herb use were (OR; 95% CI): Maroon ethnic descent (5.33; 3.26 to 8.71 vs Creole), recruitment at the STI clinic (2.04; 1.24 to 3.36 vs FP), lower education levels (3.80; 1.68 to 8.57 lower vs higher, and 2.02; 0.90 to 4.51 middle vs higher). Lower age and recruitment at the STI clinic were associated with *C. trachomatis* infection, but not vaginal herb use.

**Conclusion:**

In Suriname, vaginal herb use is common among Maroon and Creole women. Education, ethnic group and recruitment site were determinants for herb use. Vaginal herb use was not a determinant of *C. trachomatis* infection. Future research should focus on the effect of herb use on the vaginal microbiome and mucosal barrier.

**Strengths and limitations of this study**

- This is the first study into the relationship between vaginal herb use and a sexually transmitted infection (STI) in Suriname.
- Moreover, determinants such as education and ethnic group are related to vaginal herb use.
- Patients were recruited at two different settings, an STI outpatient clinic versus a family planning clinic, allowing a broader analysis of various populations.
- We did not assess if vaginal herbs were used intravaginally and/or externally.
- We did not study the effect of vaginal herb use on the vaginal microbiota.

## Introduction

Depending on cultural habits, women worldwide engage in a variety of vaginal hygiene practices, such as the use of intravaginal douches, herbal vaginal steam baths or the direct insertion of herbs into the vagina.^1–4^ Studies in the USA showed that women who had less income, less education, were unmarried, lived in the southern States and were of African–American descent were more likely to engage in such practices when compared with white women.^2^

Vaginal practices are intended for feeling clean and fresh, getting rid of vaginal malodour, or removing residual menstrual blood,^5 6^ and for improving the appearance of the vagina and for enhancing sensation during intercourse, and securing the relationship with, and economic support by the male partner.^2^ However, these practices may increase the risk of acquiring infections, such as HIV,^4^ human papiloma virus,^7^ but also bacterial sexually transmitted infections (STI) and trichomoniasis.^8^ Particularly, intravaginal practices, such as use of water and soap, the insertion of a cloth or a piece of paper into the vagina, to dry and tighten the vagina was associated with acquiring HIV.^4^ An underlying mechanism for this may be that intravaginal practices deplete the relative amount of vaginal *Lactobacillus sp.* 4 9 Lactobacilli produce antimicrobials and acidify the vagina by lactic-acid production and are thus considered to be a hallmark for vaginal health; their depletion is known to mediate HIV and STI acquisition.^4 10–13^

Risk for STI may be further increased by a breakdown of the vaginal epithelial barrier and sometimes even the occurrence of lacerations.^3^ In addition, a lack of vaginal fluids increases the risk of condom rupture. Although several studies have reported an association between vaginal practices and STI,^1 8 14^ other studies did not.^15 16^ Unfortunately, earlier studies did not consider the type of vaginal practice nor the frequency of their use.

Suriname is located on the northeast coast of South America, bordering the Atlantic Ocean to the north and surrounded by French Guiana to the east, Brazil to the south and Guyana to the west. The population of approximately 570 000 is characterised by the ethnical and cultural diversity. The largest ethnic groups are the Creoles and Maroons (both originating from enslaved Africans imported in the 17th to 19th century), and the Hindustani and Javanese (descendants from indentured labourers from the former British Indies and Dutch Indies respectively, who arrived around the turn of the 20th century). There are, in addition, Indigenous Amerindians, people of mixed descent, and descendants from Chinese labourers, European colonists and immigrants from various Latin American and Caribbean countries such as Brazil, Guyana, French Guiana, Haiti and Cuba.^17^

Among these ethnic groups, Afro-Surinamese women (Creoles and Maroons) in particular engage in a variety of vaginal practices including vaginal steam baths containing certain herbs for drying and tightening the vagina, cleansing after menstruation or after birth to prevent puerperal fever, ‘placing the uterus back into position’, or preventing a flabby abdominal wall.^3^ Typically, for a herbal genital steam bath, a woman sits with spread legs on a bucket or bidet containing warm water with certain herbs to steam her inner genital parts. Depending on the herbs, the cooled down bath is used to wash the genital parts after steaming.^18^ For direct internal application, Dettol (the brand name of the antiseptic chloroxylenol), yoghurt, lemon juice, vinegar as well as commercial products such as effervescent tablets, gels or emulsions are used. Commercial products in the form of wipes, emulsions, crèmes and mousses can also be used externally.^3 18^

In this study, we examined the type and frequency of vaginal practices in women visiting an STI outpatient clinic and a family planning (FP) clinic; in particular, the use of herbs and the reasons for their use were identified. Moreover, we assessed factors associated with vaginal herb use, and examined whether vaginal herb use is a determinant of cervicovaginal *Chlamydia trachomatis* infection.

## Methods

The data used for the current study were gathered in the course of a larger study concerning chlamydia, the Urogenital Chlamydia Rapid Test Evaluation in Paramaribo and Amsterdam (CUSTEPA) study.^17 19 20^

### Study population

Recruitment took place in Suriname’s capital city Paramaribo in the period 2009–2010 at two locations: the Dermatological Service, an integrated outpatient STI clinic that offers free-of-charge examination and treatment of STIs and infectious skin diseases such as leprosy and leishmaniasis; and the Lobi Foundation, an FP clinic. The study was cross sectional, and each participant was given a unique code. Subsequently, a nurse interviewed the participant about demographic characteristics, including self-reported ethnic background, sexual behaviour, symptoms, STI history and vaginal hygiene. Criteria for exclusion were age below 18 years and previous participation within the CUSTEPA study.

### Patient and public involvement

Representatives of the Maroon population were consulted on the outcome of the study.

### Vaginal hygiene practices

Using a structured questionnaire, nurses asked women in detail about the use of products for vaginal practices such as douches, herbs or other home-made products, and if so, at which frequency. The questionnaires included several options such as the use of herbal genital steam baths, vinegar, water, yoghurt, and two often used commercial products: Lactacyd (Omega Pharma Nederland BV, Rotterdam, The Netherlands) that consists of lactic acid-containing wipes, crèmes, emulsion and mousses, and Dettol (Reckitt Benckiser Group, Slough, UK). Participants could specify the products they used. Precoded answer options for the frequency of vaginal practices were: daily, weekly, monthly or less than once a month. Women who used vaginal herbs were asked about their most recent application, and the reasons for use (hygiene, sexual pleasure, health or other). Items mentioned under reason ‘other’ that fitted one of the three former reasons were regrouped accordingly.

### Specimen collection and testing procedures

Vaginal swabs were collected by trained nurses, stored and shipped to the Public Health Laboratory in Amsterdam, as described earlier.^19^ Here, the samples were tested for *C. trachomatis* rRNA (APTIMA CT, Hologic Gen-Probe, San Diego, USA) according to the manufacturer’s instructions. Test results were sent to the clinics in Suriname, and participants (and partners if indicated) were managed as described earlier.^19^

### Statistical analysis

To examine whether epidemiological characteristics and behaviour, including the use and frequency of vaginal practices, differed between women from the five major ethnic groups, characteristics and behaviours of these groups were compared using the χ^2^ test; age was compared using the Kruskal-Wallis test. Univariable logistic regression analysis was used to assess determinants for the use of vaginal herbs. In univariable analysis, the following variables were assessed: age, education, ethnic group, ethnicity of sexual partner, recruitment site, condom use, number of sexual partners in the preceding 1 and 12 month(s), having had sex in exchange for money or goods, and vaginal symptoms. Variables with p<0.05 in univariable analysis were entered into multivariable logistic regression models; variables were removed stepwise until only significant variables were retained, but based on earlier studies age was forced into the model.^1 2^ A second logistic regression analysis was conducted to assess whether vaginal herb use was independently associated with *C. trachomatis* infection. Variables univariately associated with *C. trachomatis* infection were entered into a multivariable model, and stepwise removed until only significant variables were retained; vaginal herb use was forced into the model. P values <0.05 were considered statistically significant. SPSS statistics V.21 (IBM) was used for the analysis.

## Results

### Study population

We initially included 1093 women who self-identified with one of the following ethnic backgrounds: 17 (1.6%) Caucasian, 12 (1.1%) Chinese, 278 (25.4%) Creole, 256 (23.4%) Hindustani, 19 (1.7%) Indigenous, 163 (14.9%) Javanese, 138 (12.6%) Maroon, 205 (18.8%) mixed descent and 5 unknown (0.5%). Due to the small numbers, we excluded Caucasian, Chinese, Indigenous women and women with unknown background from further analysis. As a result, the study population included 1040 women of either Creole, Hindustani, Javanese, Maroon or mixed descent (table 1).

**Table 1:** Epidemiological characteristics of Creole, Hindustani, Javanese, Maroon and mixed descent women (n=1040) recruited at the family planning clinic and sexually transmitted infections (STI) clinic, Paramaribo, Suriname, 2009–2010

	Creole	Hindustani	Javanese	Maroon	Mixed descent	P value
	(n=278)	(n=256)	(n=163)	(n=138)	(n=205)	
	n (%)	n (%)	n (%)	n (%)	n (%)	
Demographic characteristics						
Recruitment site						<0.001
Family planning clinic	199 (71.6)	226 (88.3)	146 (89.6)	85 (61.6)	138 (67.3)	
STI clinic	79 (28.4)	30 (11.7)	17 (10.4)	53 (38.4)	67 (32.7)	
Median age in years (IQR)	29 (24–35)	31 (26–39)	30 (25–36)	28 (23–34)	27 (23–33)	<0.001
Age in years						
<25	81 (29.1)	44 (17.2)	39 (23.9)	48 (34.8)	69 (33.7)	0.001
25–29	64 (23.0)	63 (24.6)	41 (25.2)	29 (21.0)	53 (25.9)	
30–34	60 (21.6)	50 (19.5)	33 (20.2)	30 (21.7)	40 (19.5)	
≥35	73 (26.3)	99 (38.7)	50 (30.7)	31 (22.5)	43 (21.0)	
Education						<0.001
Low	83 (29.9)	103 (40.2)	51 (31.3)	78 (56.5)	40 (19.5)	
Medium	147 (52.9)	130 (50.8)	89 (54.6)	41 (29.7)	109 (53.2)	
High	43 (15.5)	21 (8.2)	21 (12.9)	9 (6.5)	52 (25.4)	
Unknown	5 (1.8)	2 (0.8)	2 (1.2)	10 (7.2)	4 (2.0)	
Symptoms						
Any symptoms	204 (73.4)	181 (70.7)	120 (73.6)	117 (84.8)	140 (68.3)	0.012
Dysuria†	60 (21.8)	80 (31.2)	39 (23.9)	46 (33.6)	42 (20.6)	0.008
Dyspareunia‡, §	66 (24.3)	73 (29.0)	41 (25.2)	41 (31.1)	51 (24.9)	0.506
Change in fluor/vaginal discharge¶	149 (54.0)	126 (49.2)	92 (56.4)	79 (57.7)	110 (53.7)	0.488
Irregular menstruation**	81 (29.3)	71 (28.1)	32 (19.9)	51 (38.1)	52 (25.7)	0.012
Abdominal pain††	106 (39.8)	106 (41.9)	67 (42.1)	74 (54.4)	78 (38.6)	0.041
Vaginal hygiene						
Performs vaginal hygiene	109 (39.2)	51 (19.9)	29 (17.8)	112 (81.2)	78 (38.0)	<0.001
Vaginal products used‡‡						
Water	35 (12.6)	28 (10.9)	17 (10.4)	29 (21.0)	31 (15.1)	0.039
Herbs	70 (25.2)	9 (3.5)	8 (4.9)	95 (68.8)	29 (14.1)	<0.001
Lactacyd	9 (3.2)	5 (2.0)	1 (0.6)	2 (1.4)	14 (6.8)	0.003
Vinegar	9 (3.2)	2 (0.8)	4 (2.5)	2 (1.4)	9 (4.4)	0.118
Dettol	4 (1.4)	4 (1.6)	0 (0.0)	0 (0.0)	0 (0.0)	0.105
Other§§	10 (3.6)	7 (2.7)	2 (1.2)	1 (0.7)	4 (2.0)	0.314
Frequency of vaginal hygiene¶¶, ***						0.027
Daily	36 (43.9)	11 (32.4)	7 (41.2)	53 (63.1)	25 (39.7)	
Once a week	18 (22.0)	12 (35.3)	3 (17.6)	19 (22.6)	11 (17.5)	
Once a month	19 (23.2)	7 (20.6)	5 (29.4)	7 (8.3)	20 (31.7)	
Less than once a month	9 (11.0)	4 (11.8)	2 (11.8)	5 (6.0)	7 (11.1)	
Reason for vaginal use of herbs‡‡,†††, ‡‡‡						
Hygiene	50 (71.4)	4 (44.4)	5 (62.5)	49 (51.6)	16 (55.2)	0.107
Sexual	24 (34.3)	0 (0.0)	1 (12.5)	46 (48.4)	11 (37.9)	0.015, 0.485
Health	11 (15.7)	0 (0.0)	1 (12.5)	16 (16.8)	2 (6.9)	–*
Other	3 (4.3)	0 (0.0)	0 (0.0)	6 (6.3)	0 (0.0)	
Last vaginal use of herbs§§§, ‡‡‡						–*
Today	7 (12.7)	0 (0.0)	0 (0.0)	19 (26.4)	1 (5.9)	
Yesterday	5 (9.1)	0 (0.0)	0 (0.0)	23 (31.9)	1 (5.9)	
Last week	20 (36.4)	0 (0.0)	2 (33.3)	15 (20.8)	4 (23.5)	
More than a week ago	23 (41.8)	4 (100.0)	4 (66.7)	15 (20.8)	11 (64.7)	
Sexual behaviour						
Number of partners in the preceding month¶¶¶						<0.001
0	17 (6.2)	8 (3.1)	2 (1.2)	10 (7.5)	11 (5.7)	
1	237 (87.1)	236 (92.5)	148 (91.4)	117 (87.3)	158 (82.3)	
2	16 (5.9)	6 (2.4)	11 (6.8)	7 (5.2)	10 (5.2)	
>2	2 (0.7)	5 (2.0)	1 (0.6)	0 (0.0)	13 (6.8)	
Condom use during sex****						<0.001
Always	49 (18.0)	17 (6.7)	10 (6.2)	23 (16.8)	37 (18.0)	
Never or inconsistent	223 (82.0)	236 (93.3)	152 (93.8)	114 (83.2)	168 (82.0)	
Number of partners in the preceding 12 months						<0.001
0	8 (2.9)	12 (4.7)	5 (3.1)	11 (8.0)	11 (5.4)	
1	208 (74.8)	209 (81.6)	123 (75.5)	82 (59.4)	127 (62.0)	
2	35 (12.6)	27 (10.5)	20 (12.3)	33 (23.9)	35 (17.1)	
>2	27 (9.7)	8 (3.1)	15 (9.2)	12 (8.7)	32 (15.6)	
Ethnic sexual mixing						
Reported≥1 sexual partner from another ethnic group	89 (32.0)	45 (17.6)	80 (49.1)	43 (31.2)	123 (60.0)	<0.001
Sex in exchange for money or goods§	7 (2.6)	6 (2.4)	0 (0.0)	5 (3.7)	19 (9.4)	<0.001
*Chlamydia trachomatis* infection††††	37 (13.3)	16 (6.2)	25 (15.3)	15 (10.9)	29 (14.1)	0.022

*P values could not be obtained due to low numbers.

†Five missings.

‡Pain during sexual intercourse.

§Sixteen missings.

¶Three missings.

**Fourteen missings.

††Twenty-four missings.

‡‡Multiple options could be chosen.

§§Other vaginal practices are products like lactacyd but from other brands; soap, antifungal and eggs.

¶¶Ninety-nine missings.

***The denominator for the percentages is the group of women who indicated they performed vaginal hygiene.

†††Fifty-six missings, % of those who filled in the question,.

‡‡‡The denominator for the percentages is the group of women who indicated they performed vaginal hygiene.

§§§Fifty-seven missings.

¶¶¶Twenty-five missings.

****Eleven missings.

††††As diagnosed by nucleic acid amplification test.

The majority of participants were recruited at the FP clinic (between 61.6% and 89.6% per ethnic background). The median age ranged from 27 years (IQR, 23–33 years) for mixed descent women to 31 years (IQR, 26–39 years) for Hindustani women (p<0.001). Most women from Maroon descent (56.5%) had lower education whereas most of those from the other ethnic backgrounds had at least medium education (p<0.001).

Vaginal symptoms were reported frequently, ranging from 68.3% (mixed descent women) to 84.8% (Maroon women; p=0.01). In all ethnic groups, more than 80% of women reported one sexual partner during the preceding month, and more than 60% reported one sexual partner during the preceding 12 months. Discordant mixing (intercourse with a partner of a different ethnic group) was most frequently seen among mixed descent and Javanese women (60.0% and 49.1%, respectively), followed by Creole, Maroon and Hindustani women. The prevalence of *C. trachomatis* infections was highest in Javanese women (15.3%) followed by mixed descent women (14.1%) and Creole women (13.3%) (p=0.02).

### Vaginal hygiene

Table 1 shows the ways in which vaginal hygiene was performed within the study population. Vaginal hygiene was most common in Maroon women (81.2%) followed by Creole (39.2%) and mixed descent women (38.0%). Between 10.4% and 21.0% of women used tap water for vaginal cleansing, with the largest proportion in Maroon (21.0%) and mixed descent women (15.1%). Lactacyd, vinegar and other substances such as Dettol were used less often.

In those women that performed vaginal hygiene, daily use was reported by Maroons (63.1%), Creoles (43.9%), Javanese (41.2%), mixed descent (39.7%) and Hindustani (32.4%).

### Vaginal herb use

Vaginal herb use was reported most frequent by Maroon women (68.8%), followed by Creole women (25.2%), women of mixed descent (14.1%), Javanese (4.9%) and Hindustani (3.5%) (table 1). Hygiene was the most often mentioned reason for vaginal herb use (across all groups ranging from 68.1% to 100%), followed by sexual pleasure (ranging from 0% to 63.9%). Due to low numbers of women reporting vaginal herb use among women of Hindustani, Javanese and mixed descent, only Maroon and Creole women were included in the further analyses of vaginal herb use.

In univariable analysis, vaginal herb use was significantly associated with recruitment location, education, ethnic background, vaginal symptoms and abdominal pain (table 2). Although not significant, younger women reported vaginal herb use more often than women aged 35 years and above. In multivariable analysis, vaginal herb use was significantly associated with recruitment at the STI clinic (OR 2.04; 95% CI 1.24 to 3.36 vs the FP clinic), lower education levels (OR 3.80; 95% CI 1.68 to 8.57 lower vs higher education; and OR 2.02; 95% CI 0.90 to 4.51 medium vs higher education) and Maroon ethnicity (OR, 5.33; 95% CI 3.26 to 8.71 vs Creole). Although not significant in multivariable analysis, younger age was associated with vaginal herb use.

**Table 2:** Univariable and multivariable logistic regression analyses of determinants associated with vaginal herb use among Creole and Maroon women, Paramaribo, Suriname, 2009–2010

	Use of vaginal herbs	Univariable OR (95% CI)	P value	Multivariable-adjusted OR (95% CI)*	P value
	n/N (%)				
Demographic characteristics					
Ethnic groups					
Creole	70/278 (25.2)	1	<0.001	1	<0.001
Maroon	95/138 (68.8)	6.57 (4.18 to 10.30)		5.33 (3.26 to 8.71)	
Recruitment site					
Family planning clinic	96/284 (33.8)	1	<0.001	1	0.005
STI clinic	69/132 (52.3)	2.15 (1.41 to 3.27)		2.04 (1.24 to 3.36)	
Age in years			0.068		0.067
<25	59/129 (45.7)	1.99 (1.15 to 3.42)		2.23 (1.17 to 4.27)	
25–29	35/93 (37.6)	1.42 (0.79 to 2.57)		1.78 (0.88 to 3.57)	
30–34	40/90 (44.4)	1.88 (1.04 to 3.40)		2.23 (1.13 to 4.42)	
≥35	31/104 (29.8)	1		1	
Education†			<0.001		0.002
Low	88/161 (54.7)	5.06 (2.38 to 10.79)		3.80 (1.68 to 8.57)	
Medium	59/188 (31.4)	1.92 (0.90 to 4.09)		2.02 (0.90 to 4.51)	
High	10/52 (19.2)	1		1	
Sexual behaviour					
Number partners preceding month‡			0.106		
0	10/27 (37.0)	1			
1	135/354 (38.1)	1.05 (0.47 to 2.36)			
≥2	15/25 (60.0)	2.55 (0.83 to 7.80)			
Number partners preceding 12 months			0.216		
0	9/19 (47.4)	1			
1	107/290 (36.9)	0.65 (0.26 to 1.65)			
≥2	49/107 (45.8)	0.94 (0.35 to 2.50)			
Ethnic sexual mixing					
No	115/284 (40.5)	1	0.612		
Yes	50/132 (37.9)	0.90 (0.59 to 1.37)			
Sex in exchange for money or goods§					
No	155/396 (39.1)	1	0.191		
Yes	7/12 (58.3)	2.17 (0.68 to 6.98)			
Symptoms					
Any symptoms					
No	24/95 (25.3)	1	0.001		
Yes	141/321 (43.9)	2.32 (1.39 to 3.87)			
Dysuria¶					
No	114/306 (37.3)	1	0.104		
Yes	49/106 (46.2)	1.45 (0.93 to 2.26)			
Dyspareunia (pain during sexual intercourse)**					
No	113/297 (38.0)	1	0.287		
Yes	47/107 (43.9)	1.28 (0.82 to 2.00)			
Change in fluor/vaginal discharge††					
No	71/185 (38.4)	1	0.619		
Yes	93/228 (40.8)	1.11 (0.74 to 1.65)			
Irregular menstruation‡‡					
No	102/278 (36.7)	1	0.122		
Yes	59/132 (44.7)	1.40 (0.92 to 2.13)			
Abdominal pain§§					
No	78/222 (35.1)	1	0.026		
Yes	83/180 (46.1)	1.58 (1.06 to 2.36)			

*ORs in the multivariable model are adjusted for all factors for which adjusted ORs are shown. In the final model, 401 participants were included.

†Fifteen missings.

‡Ten missings.

§Eight missings.

¶Four missings.

**Twelve missings.

††Three missings.

‡‡Six missings.

§§Fourteen missings.

STI, sexually transmitted infections.

As described in table 3, a wide variety of herbs were used. Maroon women predominantly used ‘kill somebody’ or ‘kill your darling *Dimorphandra conjugata* (Splitg.) Sandw. (Fabaceae)’,^13^ the ‘jambolan or dyamu *Syzygium cumini* (L.) Skeels (Myrtaceae)’,^9^ the ‘towel or wasduku *Clidemia capitellata* (Bonpl.) D. Don (Melastomataceae)’,^9^ the ‘guavaberry or andoya *Campomanesia aromatica* (Aubl.) Griseb. (Myrtaceae)’,^6^ and ‘pikin bë/witi baka píyjá páu (small red/white backed pineapple) *Vismia* Vand. sp. (Hypericaceae)’.^7^ Creole women mostly mentioned ‘sea island cotton or redikatun *Gossypium barbadense* L. (Malvaceae)’^16^ in addition to the leaves of the ‘tropical-almond or amandra *Terminalia catappa* L. (Combretaceae)’, the ‘mess apple or broko pi (‘broken penis’) *Bellucia grossularioides* (L.) Triana (Melastomataceae)’, the ‘guava or guyaba *Psidium guajava* L. (Myrtaceae)’, the ‘jungle geranium or faya lobi (‘fiery love’) *Ixora coccinea* L. (Rubiaceae)’, and the ‘ant bush or kapasiwiwiri (‘herb of the nine-banded armadillo’) *Siparuna guianensis* Aubl. (Monimiaceae)’.

**Table 3:** Vaginal products mentioned among female participants performing vaginal hygiene, Paramaribo, Suriname, 2009–2010

Vaginal product (scientific names if applicable)	Maroon ethnicity (n=112)	Creole ethnicity (n=109)	Other ethnicities* (n=158)
Almond leaves (*Terminalia catappa*)	1	3	1
Andoya (*Campomanesia aromatica*)	6	1	1
Blaka masusa	0	1	0
Blaka uma	0	5	2
Broko pie (*Bellucia grossularioides*)	1	1	0
Dettol	0	4	4
Djamu (*Syzygium cumini*)	9	4	0
Douche gel	0	0	1
Dram (alcohol)	0	1	0
Eggs	0	1	0
Eva products	0	1	0
Faya lobi (*Ixora coccinea)*	2	0	0
Feififinga wiwiri	1	1	0
Guave (*Psidium guajava)*	3	2	0
Intimate wash products	0	3	5
Kill somebody (*Dimorphandra conjugata)*	13	4	1
Lactacyd	2	9	20
Manjablad (Mango leaves)	4	9	3
Odany jewa	0	0	3
Paraklem	4	1	0
Pedreku	1	1	2
Pikin bë (*Vismia* Vand. sp)	7	5	14
Redikatun (*Gossypium barbadense*)	4	16	6
Suku trobi	7	3	0
Twigs	0	0	1
Uma anesi	4	1	2
Unknown leaves	13	6	6
Vagisil	0	1	0
Vinegar	2	9	15
Wasduku (*Clidemia capitellata*)	9	1	3
Water	29	35	76
Yarakopie	2	3	1
Yoghurt	0	0	1

*Hindustani, Javanese and mixed race ethnicity.

### Vaginal herb use and *C. trachomatis* infection

Vaginal herb use was not associated with *C. trachomatis infection*, neither in univariable nor in multivariable analysis (adjusted OR [aOR], 1.20; 95% CI 0.65 to 2.22, p=0.564; table 4). In contrast, recruitment location, age and reporting sex in exchange for money or goods were associated with *C. trachomatis* infection in univariable analysis. In multivariable analysis, chlamydia infection was associated with recruitment at the STI clinic versus FP clinic (OR, 2.59; 95% CI 1.39 to 4.83; p=0.003) and also with younger age, both for <25 years, and 25–29 years versus ≥35 years (resp aOR, 4.37; 95% CI 1.59 to 12.00, aOR 4.96; 95% CI 1.72 to 14.26 and aOR 1.20; 95% CI 0.33 to 4.35; p=0.002).

**Table 4:** Univariable and multivariable logistic regression analyses of variables associated with cervicovaginal *Chlamydia trachomatis* infection among Creole and Maroon women, Paramaribo, Suriname, 2009–2010

	NAAT positive	Univariable OR (95% CI)	P value	Multivariable-adjusted OR (95% CI)*	P value
	n/N (%)				
Demographic characteristics					
Ethnic group					
Creole	37/278 (13.3)	1	0.479		
Maroon	15/138 (10.9)	0.79 (0.42 to 1.50)			
Recruitment site					
Family planning clinic	25/284 (8.8)	1	0.001	1	0.003
STI clinic	27/132 (20.5)	2.66 (1.48 to 4.80)		2.59 (1.39 to 4.83)	
Age in years			0.001		0.002
<25	25/129 (19.4)	4.76 (1.75 to 12.92)		4.37 (1.59 to 12.00)	
25–29	17/93 (18.3)	4.43 (1.56 to 12.54)		4.96 (1.72 to 14.26)	
30–34	5/90 (5.6)	1.17 (0.33 to 4.16)		1.20 (0.33 to 4.35)	
≥35	5/104 (4.8)	1		1	
Education			0.444		
Low	19/161 (11.8)	1.03 (0.39 to 2.73)			
Medium	23/188 (12.2)	1.07 (0.41 to 2.78)			
High	6/52 (11.5)	1			
Unknown	4/15 (26.7)	2.79 (0.67 to 11.60)			
Vaginal hygiene					
Performed vaginal hygiene					
No	21/195 (10.8)	1	0.317		
Yes	31/221 (14.0)	1.35 (0.75 to 2.44)			
Herb use					
No	27/251 (10.8)	1	0.187	1	0.564
Yes	25/165 (15.2)	1.48 (0.83 to 2.66)		1.20 (0.65 to 2.22)	
Frequency of performing vaginal hygiene†					
Daily	16/89 (18.0)	1.82 (0.90 to 3.68)	0.282		
At least once a week	7/36 (19.4)	2.00 (0.78 to 513)			
At least once a month	2/25 (8.0)	0.72 (0.16 to 3.28)			
Less then once a month	3/14 (21.4)	2.26 (0.58 to 8.76)			
Never	21/195 (10.8)	1			
Sexual behaviour					
Number of partners preceding month‡					
0–1	43/381 (11.3)	1	0.018		
≥2	7/25 (28.0)	3.06 (1.21 to 7.74)			
Condom use§					
Always	11/72 (15.3)	1.30 (0.63 to 2.68)	0.473		
Never or inconsistent	41/337 (12.2)	1			
Number of partners in the preceding 12 months					
0–1	37/309 12.0)	1	0.582		
≥2	15/107 (14.0)	1.20 (0.63 to 2.28)			
Ethnic sexual mixing					
Reported only sexual partners from own ethnic group	32/284 (11.3)	1	0.267		
Reported at least one sexual partner from another ethnic group	20/132 (15.2)	1.41 (0.77 to 2.57)			
Sex in exchange for money or goods¶					
No	47/396 (11.9)	1	0.038		
Yes	4/12 (33.3)	3.71 (1.08 to 12.81)			

*ORs in the multivariable model are adjusted for all factors for which adjusted ORs are shown. In the final model, 416 participants were included.

†Fifty-seven missings.

‡Ten missings.

§Seven missings.

¶Eight missings.

NAAT, nucleic acid amplification test; STI, sexually transmitted infection.

## Discussion

We assessed the use and frequency of various vaginal practices among female STI clinic and FP clinic visitors in Suriname, and found that: (1) vaginal practices are commonly used, and most frequently by Maroon, Creole and mixed decent women; (2) vaginal herb use is more common among women with lower education; (3) vaginal herb use is most frequently practised for hygienic reasons and (4) vaginal herb use is not associated with *C. trachomatis* infection.

Ethnic background was the most important determinant for vaginal herb use. The extensive vaginal use of herbs among Maroon women has previously been described in French Guiana, where a prevalence of 96.1% was found.^21^

Women who experienced vaginal symptoms were more likely to engage in vaginal herb use. Since this is a cross-sectional study, it is not clear if vaginal symptoms are a cause, or an effect of vaginal practices. A Cambodian study also found an association between vaginal douching and vaginal symptoms.^22^ Similarly, female sex workers in China were more likely to engage in vaginal practices when having STI-related symptoms, and reportedly engaged more in vaginal douching when experiencing vaginal symptoms.^23^ In contrast, a study with Jamaican women who attended a public STI clinic found that vaginal itching led to a lower frequency of vaginal douching.^24^ Prospective (intervention) studies could shed light on any casual links between vaginal practices and vaginal symptoms. The high prevalence of vaginal herb use among Maroon women has been previously reported, and it is conceivable that Maroon women do not engage in vaginal herb use as consequence of vaginal symptoms but rather out of (cultural) habit.^21^

As previously described, hygiene was the most important reason for vaginal herb use, followed by sexual reasons.^3^ Moreover, Maroon women mentioned as a reason to make oneself more attractive for one’s partner.

We found no association between vaginal herb use and infection with *C. trachomatis*. This confirms earlier findings of the above-mentioned Chinese study where no association was found among sex workers between vaginal practices (mostly disinfectants after sex with clients) and STI (syphilis, *Neisseria gonorrhoea* and *C. trachomatis* combined).^23^

This study has some limitations. This study did not assess the mode of vaginal practices, for example, whether the products were applied intravaginally and/or externally. Previous studies have found associations between the internal use of vaginal practices and HIV.^4^ Therefore, we cannot exclude that the lack of association between vaginal herb use and *C. trachomatis* infection may be explained by predominantly external use of herbs. Moreover, we did not study the effect of vaginal herb use on the vaginal microbiota. Lactobacilli generally constitute a healthy vaginal microbiota as they generate an acidic environment (pH 4.0–4.5) and produce antimicrobials, that restrict the growth of most pathogens.^25^ A non-lactobacillus-dominated vaginal microbiota is thus considered dysbiotic. Intravaginal practices have been linked to developing vaginal dysbiosis,^4 9 26 27^ but these studies studied douching behaviour in general and did not differentiate between different types of intravaginal products. The use of an over-the-counter lactic acid containing douche was recently studied prospectively among healthy Dutch women and this was not found to significantly impact the vaginal microbiota composition, although an increased odds for having non-lactobacillus-dominated vaginal microbiota among users was observed.^28^ Additionally, douching significantly increased the odds for testing positive for *Candida albicans.* Three douching intervention studies failed to show a significant effect of douching cessation on the vaginal microbiota,^9 29^ although one study did observe significantly reduced candidiasis prevalence.^30^ The specific effect of herb use on the vaginal microbiota is currently unknown and may differ by study population and by specific herb used. Compared with Caucasian women, women of African descent are more likely to have vaginal microbiota not dominated by *Lactobacillus sp.* 11 31 32 More research is needed to evaluate the impact of vaginal herb use on the vaginal microbiome and its potential to cause dysbiosis. Since vaginal herb use involves female hygiene, sexuality and cultural identity, it is considered a sensitive subject in Surinamese society.^33^ Therefore, the anthropological and psychological aspects of vaginal herb use should be studied in detail to shed more light on this widely used phenomenon.

In conclusion, in the multiethnic society of Suriname, many Maroon and Creole women use vaginal herbs. Apart from ethnic group, education and being recruited at an STI clinic (as opposed to an FP clinic) were the main determinants for vaginal herb use. Vaginal herb use was not associated with *C. trachomatis* infection. Whether vaginal herb use has beneficial or possible negative effects on female health needs to be assessed in future studies.

## Supplementary Material

Reviewer comments

Author's manuscript

## References

[1] Blythe MJ, Fortenberry JD, Orr DP Douching behaviors reported by adolescent and young adult women at high risk for sexually transmitted infections. J Pediatr Adolesc Gynecol 2003;16:95–100. 10.1016/S1083-3188(03)00027-5 12742144

[2] Misra DP, Trabert B, Atherly-Trim S Variation and predictors of vaginal douching behavior. Womens Health Issues 2006;16:275–82. 10.1016/j.whi.2006.03.005 17055380 PMC1832159

[3] van Andel T, de Korte S, Koopmans D, et al Dry sex in Suriname. J Ethnopharmacol 2008;116:84–8. 10.1016/j.jep.2007.11.003 18083316

[4] Low N, Chersich MF, Schmidlin K, et al Intravaginal practices, bacterial vaginosis, and HIV infection in women: individual participant data meta-analysis. PLoS Med 2011;8:e1000416 10.1371/journal.pmed.1000416 21358808 PMC3039685

[5] Oh MK, Merchant JS, Brown P Douching behavior in high-risk adolescents. What do they use, when and why do they douche? J Pediatr Adolesc Gynecol 2002;15:83–8. 12057529 10.1016/s1083-3188(01)00148-6

[6] Markham CM, Tortolero SR, Addy RC, et al Factors associated with frequent vaginal douching among alternative school youth. J Adolesc Health 2007;41:509–12. 10.1016/j.jadohealth.2007.05.022 17950172 PMC2083649

[7] Bui TC, Thai TN, Tran LT, et al Association between vaginal douching and genital human papillomavirus infection among women in the United States. J Infect Dis 2016;214:1370–5. 10.1093/infdis/jiw388 27553042 PMC6281373

[8] Tsai CS, Shepherd BE, Vermund SH Does douching increase risk for sexually transmitted infections? A prospective study in high-risk adolescents. Am J Obstet Gynecol 2009;200:38.e1–38.e8. 10.1016/j.ajog.2008.06.026 18667177 PMC3199592

[9] Brotman RM, Klebanoff MA, Nansel TR, et al A longitudinal study of vaginal douching and bacterial vaginosis–a marginal structural modeling analysis. Am J Epidemiol 2008;168:188–96. 10.1093/aje/kwn103 18503038 PMC2574994

[10] Borgdorff H, Tsivtsivadze E, Verhelst R, et al Lactobacillus-dominated cervicovaginal microbiota associated with reduced HIV/STI prevalence and genital HIV viral load in African women. ISME J 2014;8:1781–93. 10.1038/ismej.2014.26 24599071 PMC4139719

[11] Borgdorff H, van der Veer C, van Houdt R, et al The association between ethnicity and vaginal microbiota composition in Amsterdam, the Netherlands. PLoS One 2017;12:e0181135 10.1371/journal.pone.0181135 28700747 PMC5507447

[12] Gosmann C, Anahtar MN, Handley SA, et al Lactobacillus-deficient cervicovaginal bacterial communities are associated with increased HIV acquisition in young South African women. Immunity 2017;46:29–37. 10.1016/j.immuni.2016.12.013 28087240 PMC5270628

[13] van der Veer C, Bruisten SM, van der Helm JJ, et al The cervicovaginal microbiota in women notified for chlamydia trachomatis infection: a case-control study at the sexually transmitted infection outpatient clinic in Amsterdam, The Netherlands. Clin Infect Dis 2017;64:24–31. 10.1093/cid/ciw586 27567124

[14] Turner AN, Morrison CS, Munjoma MW, et al Vaginal practices of HIV-negative Zimbabwean women. Infect Dis Obstet Gynecol 2010;2010:1–7. 10.1155/2010/387671 PMC2943083 20871844

[15] Ness RB, Hillier SL, Kip KE, et al Douching, pelvic inflammatory disease, and incident gonococcal and chlamydial genital infection in a cohort of high-risk women. Am J Epidemiol 2005;161:186–95. 10.1093/aje/kwi025 15632269

[16] Ott MA, Ofner S, Fortenberry JD Beyond douching: use of feminine hygiene products and STI risk among young women. J Sex Med 2009;6:1335–40. 10.1111/j.1743-6109.2008.01152.x 19170863 PMC3020654

[17] van der Helm JJ, Bom RJ, Grünberg AW, et al Urogenital Chlamydia trachomatis infections among ethnic groups in Paramaribo, Suriname; determinants and ethnic sexual mixing patterns. PLoS One 2013;8:e68698 10.1371/journal.pone.0068698 23874730 PMC3714285

[18] van ’t Klooster C, Haabo V, Ruysschaert S, et al Herbal bathing: an analysis of variation in plant use among Saramaccan and Aucan Maroons in Suriname. J Ethnobiol Ethnomed 2018;14:20 10.1186/s13002-018-0216-9 29544521 PMC5856216

[19] van der Helm JJ, Sabajo LO, Grunberg AW, et al Point-of-care test for detection of urogenital chlamydia in women shows low sensitivity. A performance evaluation study in two clinics in Suriname. PLoS One 2012;7:e32122 10.1371/journal.pone.0032122 22393383 PMC3290553

[20] Bom RJ, van der Helm JJ, Bruisten SM, et al The role of Surinamese migrants in the transmission of Chlamydia trachomatis between Paramaribo, Suriname and Amsterdam, The Netherlands. PLoS One 2013;8:e77977 10.1371/journal.pone.0077977 24236009 PMC3827209

[21] van Melle A, Parriault MC, Basurko C, et al Knowledge, attitudes, behaviors, and practices differences regarding HIV in populations living along the Maroni river: particularities of operational interest for Amerindian and Maroon populations. AIDS Care 2015;27:1112–7. 10.1080/09540121.2015.1032203 25909579

[22] Heng LS, Yatsuya H, Morita S, et al Vaginal douching in Cambodian women: its prevalence and association with vaginal candidiasis. J Epidemiol 2010;20:70–6. 10.2188/jea.JE20081046 20009371 PMC3900782

[23] Li J, Jiang N, Yue X, et al Vaginal douching and sexually transmitted infections among female sex workers: a cross-sectional study in three provinces in China. Int J STD AIDS 2015;26:420–7. 10.1177/0956462414543937 25015933

[24] Carter M, Gallo M, Anderson C, et al Intravaginal cleansing among women attending a sexually transmitted infection clinic in Kingston, Jamaica. West Indian Med J 2013;62:56–61. 24171329 PMC4450344

[25] Hickey RJ, Zhou X, Pierson JD, et al Understanding vaginal microbiome complexity from an ecological perspective. Transl Res 2012;160:267–82. 10.1016/j.trsl.2012.02.008 22683415 PMC3444549

[26] Hutchinson KB, Kip KE, Ness RB Gynecologic Infection Follow-Through I. Vaginal douching and development of bacterial vaginosis among women with normal and abnormal vaginal microflora. Sex Transm Dis 2007;34:671–5. 17413534 10.1097/01.olq.0000258435.34879.da

[27] McClelland RS, Richardson BA, Graham SM, et al A prospective study of risk factors for bacterial vaginosis in HIV-1-seronegative African women. Sex Transm Dis 2008;35:617–23. 10.1097/OLQ.0b013e31816907fa 18418290 PMC3902781

[28] Veer CV, Bruisten S, Houdt RV editors. O04.2 Effects of over-the-counter lactic acid-containing vaginal douching products on the vaginal microbiota. Sex Transm Infect 2017. 10.1186/s12866-019-1545-0 PMC6659218 31345159

[29] Klebanoff MA, Andrews WW, Yu KF, et al A pilot study of vaginal flora changes with randomization to cessation of douching. Sex Transm Dis 2006;33:610–3. 10.1097/01.olq.0000216050.41305.c1 16614585

[30] Sivapalasingam S, McClelland RS, Ravel J, et al An effective intervention to reduce intravaginal practices among HIV-1 uninfected Kenyan women. AIDS Res Hum Retroviruses 2014;30:1046–57. 10.1089/aid.2013.0251 25265254 PMC4208596

[31] Ravel J, Gajer P, Abdo Z, et al Vaginal microbiome of reproductive-age women. Proc Natl Acad Sci U S A 2011;108(Suppl 1):4680–7. 10.1073/pnas.1002611107 20534435 PMC3063603

[32] Fettweis JM, Brooks JP, Serrano MG, et al Differences in vaginal microbiome in African American women versus women of European ancestry. Microbiology 2014;160(Pt 10):2272–82. 10.1099/mic.0.081034-0 25073854 PMC4178329

[33] Terborg JRH Sexual behaviour and sexually transmitted diseases among Saramaka and Ndjuka Maroons in the hinterland of Suriname. Paramaribo: MZ Primary Health Care Suriname: ProHealth, 2001;83.

