# Supplementary material for: Vaginal herb use and Chlamydia trachomatis infection: cross-sectional study among women of various ethnic groups in Suriname
Source: BMJ Open. 2019 May 16;9(5):e025417. doi: 10.1136/bmjopen-2018-025417 (PMC6530446; doi:10.1136/bmjopen-2018-025417)
Supplement: Author's manuscript [file bmjopen-2018-025417.draft_revisions.pdf]

BMJ Open

BMJ Open is committed to open peer review. As part of this commitment we make the peer review history of every article we publish publicly available.

When an article is published we post the peer reviewers' comments and the authors' responses online. We also post the versions of the paper that were used during peer review. These are the versions that the peer review comments apply to.

The versions of the paper that follow are the versions that were submitted during the peer review process. They are not the versions of record or the final published versions. They should not be cited or distributed as the published version of this manuscript.

BMJ Open is an open access journal and the full, final, typeset and author-corrected version of record of the manuscript is available on our site with no access controls, subscription charges or pay-per-view fees (http://bmjopen.bmj.com).

If you have any questions on BMJ Open's open peer review process please email info.bmjopen@bmj.com

BMJ Open

Vaginal herb use and Chlamydia trachomatis infection: a cross-sectional study among women of various ethnic groups in Suriname

Journal:	*BMJ Open*
Manuscript ID	bmjopen-2018-025417
Article Type:	Research
Date Submitted by the Author:	31-Jul-2018

Complete List of Authors:	Van der Helm, Jannie; Public Health Service Amsterdam, Department of Research<br>Schim van der Loeff, Maarten; GGD Amsterdam<br>de Vries, Esther; Public Health Service Amsterdam, Department of Research<br>van der Veer, Charlotte; GGD Amsterdam, Streeklaboratorium<br>Grünberg, Antoon; Ministry of Health, Paramaribo, Suriname, Department of Public Health<br>Mans, Dennis; Department of Pharmacology<br>de Vries, H; University of Amsterdam, Department of Dermatology

Keywords:	Suriname, ethnicity, vaginal, vaginal douching, herb therapy

SCHOLARONE™ Manuscripts

For peer review only

Vaginal herb use and *Chlamydia trachomatis* infection: a cross-sectional study among women of various ethnic groups in Suriname

Running title: Vaginal herbal use and chlamydia in Suriname

Jannie J. van der Helm PhD[1], Maarten F. Schim van der Loeff MD, PhD[2,5,], Esther de Vries, MSc[2], Charlotte van der Veer[2], Antoon W. Grünberg MD, PhD[3], and Dennis R. Mans PhD[4], Henry J.C. de Vries MD, PhD[2,5]

1. Centre for Environmental Safety and Security, National Institute of Public Health and the Environment, Bilthoven
2. Public Health Service (GGD) Amsterdam, Department of Infectious diseases, Amsterdam, the Netherlands
3. Department of Public Health, Ministry of Health, Paramaribo, Suriname
4. Department of Pharmacology, Faculty of Medical Sciences, Anton de Kom University of Suriname, Paramaribo, Suriname
5. Amsterdam UMC, University of Amsterdam, department of dermatology, Amsterdam Institute for Infection and Immunity (AI&II), Meibergdreef 9, Amsterdam, Netherlands

Corresponding author:
Henry John Christiaan de Vries
Department of Dermatology, Academic Medical Center, University of Amsterdam
Meibergdreef 9, 1105 AZ Amsterdam-Zuidoost, the Netherlands,
Phone: +31 (0)20 566 9111, Fax: +31 (0)20 566 4440 email: h.j.devries@amc.nl

Word count of the abstract: 242
Word count of the text: 2872
Number of references: 33
Number of tables: 4
Number of figures: 0

Conflict of Interest: None reported

Funding: This work was supported by the Research and Development fund of the Public Health Service of Amsterdam [project no 2369 and 2371] and AGIS healthcare insurance [RVVZ no 1417000]. The funders had no role in study design, data collection and analysis, decision to publish, or preparation of the manuscript.

Contributorship statement: JvdH collected the data, analysed the data and wrote the manuscript; MSvdL supervised the analysis and wrote the manuscript; EdV analysed the data and wrote the first manuscript draft; CvdV wrote the manuscript; AWG designed the study, collected data and

For peer review only

For peer review only - http://bmjopen.bmj.com/site/about/guidelines.xhtml

wrote the manuscript; DRM collected the data and wrote the manuscript; and HdV designed the study, and wrote the manuscript.

Abstract

Objective: Vaginal steam baths with herb leaves (herb use) is practiced by some Surinamese women. We assessed herb use among women from the five most prevalent ethnic groups, and if herb use is associated with *Chlamydia trachomatis* infection.

Setting: Participants were recruited at a sexually transmitted infection clinic (STI) and a family planning clinic (FP) in Paramaribo, Suriname.

Participants: 1040 women were included subsequently, comprising the following ethnic groups: Creole (26.7%), Hindustani (24.6%), Javanese (15.7%), Maroon (13.3%) and Mixed descent (19.7%).

Methods: Nurses collected a questionnaire and vaginal swabs for nucleic acid amplification *C. trachomatis* testing.

Primary outcomes: Determinants of vaginal herb use and *C. trachomatis* infection via univariable and multivariable logistic regression.

Results: Herb use was most common among Maroon (68.8%) and Creole women (25.2%). In multivariable analysis including only Maroon and Creole women, determinants significantly associated with vaginal herb use were (odds ratio; 95%CI): Maroon ethnic descent (5.33; 3.26-8.71 versus Creole), recruitment at the STI clinic (2.04;1.24-3.36 versus FP), lower education levels (3.80;1.68-8.57 lower versus higher, and 2.02;0.90-4.51 middle versus higher). Lower age and recruitment at the STI clinic *were associated with C. trachomatis* infection, but not vaginal herb use.

Conclusion: In Suriname, vaginal herb use is common among Maroon and Creole women. Education, ethnic group and recruitment site were determinants for herb use. Vaginal herb use was not a determinant of *C. trachomatis* infection. Future research should focus on the effect of herb use on the vaginal microbiome and mucosal barrier.

For peer review only - http://bmjopen.bmj.com/site/about/guidelines.xhtml

Strengths and limitations of this study:

- This is the first study to show that vaginal herb use in Suriname is common among Maroon and Creole women (of African descent).
- Moreover, we show that education, ethnic group and recruitment site (STI outpatient clinic vs Family Planning clinic) are determinants for vaginal herb use.
- Lastly we show that vaginal herb use is not a determinant of C. trachomatis infection.
- We did not assess if vaginal herbs were used intra-vaginally and/or externally.
- We did not study the effect of vaginal herb use on the vaginal microbiota.

Keywords: herb therapy; vaginal douching; vaginal; ethnicity; Suriname

Data sharing statement: No data other than mentioned in the manuscript are available.

For peer review only

For peer review only - http://bmjopen.bmj.com/site/about/guidelines.xhtml

Introduction

Depending on cultural habits, women worldwide engage in a variety of vaginal hygiene practices, such as the use of intra-vaginal douches, herbal vaginal steam baths, or the direct insertion of herbs into the vagina.(1-4) Studies in the United States of America showed that women who had less income, less education, were unmarried, lived in the southern States, and were of African-American descent were more likely to engage in such practices when compared to white women.(2)

Vaginal practices are not only intended for feeling clean and fresh, getting rid of vaginal malodour, or removing residual menstrual blood(5, 6), but also for improving the appearance of the vagina and for enhancing sensation during intercourse, and securing the relationship with, and economic support by the male partner.(2) However, these practices may increase the risk of acquiring infections, such as HIV(4), HPV(7), but also bacterial sexually transmitted infections (STI) and trichmoniasis.(8) Particularly intra-vaginal practices, such as use of water and soap, the insertion of a cloth or a piece of paper into the vagina, to dry and tighten the vagina was associated with acquiring HIV.(4) An underlying mechanism for this may be that intra-vaginal practices deplete the relative amount of vaginal *Lactobacillus spp.*(4, 9) Lactobacilli produce anti-microbials and acidify the vagina by lactic-acid production and are thus considered to be a hallmark for vaginal health; their depletion is known to mediate HIV and STI acquisition.(4, 10-13)

Risk for STI may be further increased by a breakdown of the vaginal epithelial barrier and sometimes even the occurrence of lacerations.(3) In addition, a lack of vaginal fluids increases the risk of condom rupture. Although several studies have reported an association between vaginal practices and STI(1, 8, 14), other studies did not.(15, 16) Unfortunately earlier studies did not consider the type of vaginal practice nor the frequency of their use.

Suriname is located on the northeast coast of South America, bordering the Atlantic Ocean to the north and surrounded by French Guiana to the east, Brazil to the south, and Guyana to the west. The population of approximately 570,000 is ethnically and culturally among the most varied in the world. The largest ethnic groups are the Creoles and Maroons (both originating from enslaved Africans imported in the 17th to 19th century), and the Hindustani and Javanese (descendants from indentured laborers from the former British Indies and Dutch Indies, respectively, arrived around the turn of the 20th century). There are, in addition, Indigenous Amerindians, people of Mixed descent, and descendants from Chinese laborers, European colonists, and immigrants from various Latin American and Caribbean countries such as Brazil, Guyana, French Guiana, Haiti, and Cuba.(17)

Particularly Afro-Surinamese women (Creoles and Maroons) engage in a variety of vaginal practices including vaginal steam baths containing certain herbs for drying and tightening the vagina, cleansing after menstruation or after birth to prevent puerperal fever, ‘placing the uterus back into position’, or preventing a flabby abdominal wall.(3) Typically, for a herbal genital steam bath, a woman sits with spread legs on a bucket or bidet containing warm water with certain herbs to steam her inner genital parts. Depending on the herbs, the cooled down bath is used to wash the genital parts after steaming.(18) For direct internal application, Dettol® (the brand name of the antiseptic chloroxylenol), yoghurt, lemon juice, vinegar as well as specific commercial products such as effervescent tablets,

For peer review only - http://bmjopen.bmj.com/site/about/guidelines.xhtml

gels, or emulsions are used. Commercial products in the form of wipes, emulsions, crèmes, and mousses can also be used externally,

In this study we examined the type and frequency of vaginal practices in women, in particular the use of herbs and the reasons for their use were identified. Moreover, we assessed factors associated with vaginal herb use, and examined whether vaginal herb use is a determinant of cervicovaginal *C. trachomatis* infection.

Methods

Ethics statement

The data used for the current study were gathered in the course of a larger study concerning chlamydia, the Urogenital Chlamydia Rapid Test Evaluation in Paramaribo and Amsterdam (CUSTE-PA) study.(17, 19, 20) The study was approved by the Ethics Committees of the Ministry of Health of the Republic of Suriname (VG010-2007) and the Academic Medical Center, University of Amsterdam, The Netherlands (MEC07/127). Women participated after given written informed consent.

Study population

Recruitment took place in Suriname's capital city Paramaribo in the period 2009-2010 at two locations: the Dermatological Service, an integrated outpatient STI clinic that offers free-of-charge examination and treatment of STIs and infectious skin diseases such as leprosy and leishmaniasis; and the Lobi Foundation, a family planning (FP) clinic. The study was cross-sectional, and each participant was given a unique code. Subsequently, a nurse interviewed the participant about demographic characteristics, including self-reported ethnic background, sexual behaviour, symptoms, STI history, and vaginal hygiene. Criteria for exclusion were age below 18 years and previous participation.

Patient and Public Involvement

Representatives of the Maroon population were consulted on the outcome of the study.

Vaginal hygiene practices

Women were asked in detail about the use of products for vaginal practices such as douches, herbs, or other home-made products, and if so, at which frequency. The questionnaires included several options such as the use of herbal genital steam baths, vinegar, water, yoghurt, and two often used commercial products: Lactacyd® (Omega Pharma Nederland BV, Rotterdam, The Netherlands) that consists of lactic acid-containing wipes, crèmes, emulsion, and mousses, and Dettol® (Reckitt Benckiser Group plc, Slough, UK). Participants could specify the products they used. Pre-coded answer options for the frequency of vaginal practices were: daily, weekly, monthly, or less than once a month. Women who used vaginal herbs were asked about their reasons and most recent application.

Specimen collection and testing procedures

For peer review only - http://bmjopen.bmj.com/site/about/guidelines.xhtml

Vaginal swabs were collected by trained nurses, stored and shipped to the Public Health Laboratory in Amsterdam, as described earlier.(19) Here, the samples were tested for *C. trachomatis* rRNA (APTIMA CT, Hologic Gen-Probe Inc, San Diego, USA) according to the manufacturer's instructions. Test results were sent to the clinics in Suriname, and participants (and partners if indicated) were managed as described earlier.

Statistical analysis

To examine whether epidemiological characteristics and behaviour, including the use and frequency of vaginal practices, differed between women from the five major ethnic groups, characteristics and behaviours of these groups were compared using the χ^{2}-test; age was compared using the Kruskal-Wallis test. Univariable logistic regression analysis was used to assess determinants for the use of vaginal herbal use. In univariable analysis the following variables were assessed: age, education, ethnic group, ethnicity of sexual partner, recruitment site, condom use, number of sexual partners in the preceding one and twelve month(s), having had sex in exchange for money or goods, and vaginal symptoms. Variables with $P<0.05$ in univariable analysis were entered into multivariable logistic regression models; variables were removed stepwise until only significant variables were retained, but based on earlier studies age was forced into the model.(1, 2). A second logistic regression analysis was conducted to assess whether vaginal herb use was independently associated with *C. trachomatis* infection. Variables univariately associated with *C. trachomatis* infection were entered into a multivariable model, and stepwise removed until only significant variables were retained; vaginal herb use was forced into the model. P values < 0.05 were considered statistically significant. SPSS statistics version 21 (IBM, City, State, USA) was used for the analysis.

Results

Study population

We included 1,093 women: 17 (1,6%) Caucasian, 12 (1.1%) Chinese, 278 (25.4%) Creole, 256 (23.4%) Hindustani, 19 (1.7%) Indigenous, 163 (14.9%) Javanese, 138 (12.6%) Maroon, 205 (18.8%) Mixed descent, and 5 unknown (0.5%). Caucasian, Chinese, Indigenous, and unknown women were excluded from further analysis due to their relatively small numbers. As a result, the study population included 1,040 women of Creole, Hindustani, Javanese, Maroon, and Mixed descent (Table 1).

The majority of participants was included at the FP clinic (between 61.6% and 89.6% per ethnic background). The median age ranged from 27 years (interquartile range (IQR), 23-33 years) for Mixed descent women to 31 years (IQR, 26-39 years) for Hindustani women (P<0.001). Most women from Maroon descent (56.5%) had lower education whereas most of those from the other ethnic backgrounds had at least medium education (P<0.001).

Vaginal symptoms were reported frequently, ranging from 68.3% (Mixed descent women) to 84.8% (Maroon women; P=0.01). In all ethnic groups, more than 80% of women reported one sexual

For peer review only - http://bmjopen.bmj.com/site/about/guidelines.xhtml

partner during the preceding month, and more than 60% reported one sexual partner during the preceding 12 months. Discordant mixing (intercourse with a partner of a different ethnic group) was most frequently seen among Mixed descent and Javanese women (60.0% and 49.1%, respectively), followed by Creole, Maroon, and Hindustani women. The prevalence of *C. trachomatis* infections was highest in Javanese women (15.3%) followed by Mixed descent women (14.1%) and Creole women (13.3%) (P = 0.02).

Vaginal practices

Table 1 shows vaginal practices exercised by the different ethnic groups in Suriname. Vaginal practices were most common in Maroon women (81.2%) followed by Creole (39.2%) and Mixed descent women (38.0%). Between 10.4% and 21.0% of women used tap water for vaginal cleansing, with the largest proportion in Maroon (21.0%) and Mixed descent women (15.1%). Lactacyd®, vinegar, and other substances such as Dettol® were used much less

In those women that used vaginal practices, daily use was reported by Maroons (63.1%), Creoles (43.9%), Javanese (41.2%), Mixed descent (39.7%), and Hindustani (32.4%).

Vaginal herb use

Vaginal herb use was reported most frequent by Maroon women (68.8%), followed by Creole women (25.2%), women of Mixed descent (14.1%), Javanese (4.9%) and Hindustani (3.5%) (Table 1). Hygiene was the most often mentioned reason for vaginal herb use (across all groups ranging from 68.1% to 100%), followed by sexual pleasure (ranging from 0% to 63.9%). Due to low numbers of women reporting vaginal herb use among women of Hindustani, Javanese and Mixed descent, only Maroon and Creole women were included in the further analyses of vaginal herb use.

In univariable analysis, the use of vaginal herbs was significantly associated with recruitment location, education, ethnic background, vaginal symptoms, and abdominal pain (Table 2). Younger women tended to report vaginal herb use more often than women aged 35 years and above, but this was not significant. In multivariable analysis, herbal use was significantly associated with recruitment at the STI clinic (OR 2.04; 95%CI 1.24-3.36 versus the FP clinic), lower education levels (OR 3.80; 95%CI 1.68-8.57 lower versus higher education; and OR 2.02; 95%CI 0.90-4.51 medium versus higher education), and Maroon etnicity (OR, 5.33; 95%CI 3.26-8.71 versus Creole).Also in multivariable analysis younger age was associated with vaginal herb use, but this was not significant.

A wide variety of herbs was used for vaginal practices (Table 3). Maroon women predominantly used kill somebody or kill your darling *Dimorphandra conjugata* (Splitg.) Sandw. (Fabaceae) (13), the jambolan or dyamu *Syzygium cumini* (L.) Skeels (Myrtaceae) (9), the towel or wasduku *Clidemia capitellata* (Bonpl.) D.Don (Melastomataceae) (9), the guavaberry or andoya *Campomanesia aromatica* (Aubl.) Griseb. (Myrtaceae) (6), and pikin bë/witi baka píyjá páu (small red/white backed pineapple) *Vismia* Vand. spp. (Hypericaceae) (7). Creole women mostly mentioned 'sea island cotton or redikatun *Gossypium barbadense* L. (Malvaceae) (16) in addition to the leaves of the tropical-almond or amandra *Terminalia catappa* L. (Combretaceae), the mess apple or broko pi ('broken penis') *Bellucia grossularioides* (L.) Triana (Melastomataceae), the guava or guyaba *Psidium*

For peer review only - http://bmjopen.bmj.com/site/about/guidelines.xhtml

guajava L. (Myrtaceae), the jungle geranium or faya lobi ('fiery love') *Ixora coccinea* L. (Rubiaceae), and the ant bush or kapasiwiwiri ('herb of the nine-banded armadillo') *Siparuna guianensis* Aubl. (Monimiaceae).

V*aginal herb use and C. trachomatis infection*

*V*aginal herb use was not associated with chlamydia positivity, neither in univariable nor in multivariable analysis (aOR 1.48; (95%CI 0.83-2.66 vaginal herb users versus non-users). In contrast, recruitment location, age, and reporting sex in exchange for money or goods, were associated with *C. trachomatis* infection in univariable analysis (Table 4). In multivariable analysis, recruitment at the STI clinic (OR, 2.59 95%CI 1.39-4.83 versus FP clinic) and, younger age (e.g. OR, 4.37; 95%CI 1.59-12.00 for < 25 years, and OR, 4.96; 95% 1.72-14.62 for 25-29 years versus ≥ 35 years) were associated with chlamydia infection.

Discussion

We assessed the use and frequency of various vaginal practices among female STI clinic and FP clinic visitors in Suriname, and found that: (1) vaginal practices are commonly used, and most frequently by Maroon, Creole and Mixed decent women; (2) vaginal herb use is more common among women with lower education; (3) vaginal herb use is most frequently practiced for hygienic reasons; and (4) vaginal herb use is not associated with *C. trachomatis* infection.

Ethnic background was the most important determinant for vaginal herb use. The extensive vaginal use of herbs among Maroon women has previously been described in French Guiana, where a prevalence of 96.1% was found.(21)

Women who experienced vaginal symptoms were more likely to engage in vaginal herb use. Since this is a cross-sectional study, it is not clear if vaginal symptoms are a cause, or an effect of vaginal practices. A Cambodian study also found an association between vaginal douching and vaginal symptoms.(22) Similarly, female sex workers in China were more likely to engage in vaginal practices when having STI-related symptoms, and reportedly engaged more in vaginal douching when experiencing vaginal symptoms.(23) In contrast, a study with Jamaican women who attended a public STI clinic found that vaginal itching led to a lower frequency of vaginal douching.(24) Prospective (intervention) studies could shed light on any casual links between vaginal practices and vaginal symptoms. The high prevalence of vaginal herb use among Maroon women has been previously reported, and it is conceivable that Maroon women do not engage in vaginal herb use as consequence of vaginal symptoms but rather out of (cultural) habit.(21)

As previously described, hygiene was the most important reason for vaginal herb use, followed by sexual reasons.(3) Moreover, Maroon women mentioned as a reason to make oneself more attractive for one's partner.

We found no association between vaginal herb use and infection with *C. trachomatis*. This confirms earlier findings of the above mentioned Chinese study where no association was found among sex

For peer review only - http://bmjopen.bmj.com/site/about/guidelines.xhtml

workers between vaginal practices (mostly disinfectants after sex with clients) and STI (syphilis, *Neisseria gonorrhoea* and *C. trachomatis* combined).(23)

This study has some limitations. This study did not assess the mode of vaginal practices, e.g. whether the products were applied intra-vaginally and/or externally. Previous studies have found associations between the internal use of vaginal practices and HIV.(4) Therefore we cannot exclude that the lack of association between vaginal herb use and *C. trachomatis* infection may be explained by predominantly external use of herbs. Moreover we did not study the effect of vaginal herb use on the vaginal microbiota. Lactobacilli generally constitute a healthy vaginal microbiota as they generate an acidic environment (pH 4.0-4.5) and produce antimicrobials, that restrict the growth of most pathogens.(25) A non-lactobacillus-dominated vaginal microbiota is thus considered dysbiotic. Intra-vaginal practices have been linked to developing vaginal dysbiosis(4, 9, 26, 27), but these studies studied douching behaviour in general and did not differentiate between different types of intra-vaginal products. The use of an over-the-counter lactic-acid containing douche was recently studied prospectively among healthy Dutch women and this was not found to significantly impact the vaginal microbiota composition, although an increased odds for having non-lactobacillus dominated vaginal microbiota among users was observed.(28) Additionally, douching significantly increased the odds for testing positive for *Candida albicans.* Three douching intervention studies failed to show a significant effect of douching cessation on the vaginal microbiota(9, 29), although one study did observe significantly reduced candidiasis prevalence.(30) The specific effect of herb use on the vaginal microbiota is currently unknown and may differ by study population and by specific herb used. Compared to Caucasian Women, women of African descent are more likely to have vaginal microbiota not dominated by *Lactobacillus spp.*.(11, 31, 32) More research is needed to evaluate the impact of vaginal herb use on the vaginal microbiome and its potential to cause dysbiosis. Since vaginal herb use involves female hygiene, sexuality, and cultural identity, it is considered a sensitive subject in Surinamese society.(33) Therefore, the anthropologic and psychological aspects of vaginal herb use should be studied in detail to shed more light on this widely used phenomenon.

In conclusion, in the multi-ethnic society of Suriname, many Maroon and Creole women use vaginal herbs. Apart from ethnic group, education and being recruited at an STI clinic (as opposed to a FP clinic) were the main determinants for vaginal herb use. Vaginal herb use was not associated with *C. trachomatis* infection . Whether vaginal herb use has beneficial or possible negative effects on female health needs to be assessed in future studies.

Acknowledgements

The author would like to thank dr. L.O.A. Sabajo, all nurses and laboratory technicians of the Dermatological Service and the Lobi Foundation for data collection, and Titia Heijman for helpful suggestions

For peer review only - http://bmjopen.bmj.com/site/about/guidelines.xhtml

Bibliography

1. Blythe MJ, Fortenberry JD, Orr DP. Douching behaviors reported by adolescent and young adult women at high risk for sexually transmitted infections. J Pediatr Adolesc Gynecol. 2003;16(2):95-100.
2. Misra DP, Trabert B, Atherly-Trim S. Variation and predictors of vaginal douching behavior. Womens Health Issues. 2006;16(5):275-82.
3. van Andel T, de Korte S, Koopmans D, Behari-Ramdas J, Ruysschaert S. Dry sex in Suriname. J Ethnopharmacol. 2008;116(1):84-8.
4. Low N, Chersich MF, Schmidlin K, Egger M, Francis SC, van de Wijgert JH, et al. Intravaginal practices, bacterial vaginosis, and HIV infection in women: individual participant data meta-analysis. PLoS Med. 2011;8(2):e1000416.
5. Oh MK, Merchant JS, Brown P. Douching behavior in high-risk adolescents. What do they use, when and why do they douche? J Pediatr Adolesc Gynecol. 2002;15(2):83-8.
6. Markham CM, Tortolero SR, Addy RC, Baumler ER, McKirahan NK, Escobar-Chaves SL, et al. Factors associated with frequent vaginal douching among alternative school youth. J Adolesc Health. 2007;41(5):509-12.
7. Bui TC, Thai TN, Tran LT, Shete SS, Ramondetta LM, Basen-Engquist KM. Association Between Vaginal Douching and Genital Human Papillomavirus Infection Among Women in the United States. J Infect Dis. 2016;214(9):1370-5.
8. Tsai CS, Shepherd BE, Vermund SH. Does douching increase risk for sexually transmitted infections? A prospective study in high-risk adolescents. Am J Obstet Gynecol. 2009;200(1):38 e1-8.
9. Brotman RM, Klebanoff MA, Nansel TR, Andrews WW, Schwebke JR, Zhang J, et al. A longitudinal study of vaginal douching and bacterial vaginosis--a marginal structural modeling analysis. Am J Epidemiol. 2008;168(2):188-96.
10. Borgdorff H, Tsivtsivadze E, Verhelst R, Marzorati M, Jurriaans S, Ndayisaba GF, et al. Lactobacillus-dominated cervicovaginal microbiota associated with reduced HIV/STI prevalence and genital HIV viral load in African women. ISME J. 2014;8(9):1781-93.
11. Borgdorff H, van der Veer C, van Houdt R, Alberts CJ, de Vries HJ, Bruisten SM, et al. The association between ethnicity and vaginal microbiota composition in Amsterdam, the Netherlands. PLoS One. 2017;12(7):e0181135.
12. Gosmann C, Anahtar MN, Handley SA, Farcasanu M, Abu-Ali G, Bowman BA, et al. Lactobacillus-Deficient Cervicovaginal Bacterial Communities Are Associated with Increased HIV Acquisition in Young South African Women. Immunity. 2017;46(1):29-37.
13. van der Veer C, Bruisten SM, van der Helm JJ, de Vries HJ, van Houdt R. The Cervicovaginal Microbiota in Women Notified for Chlamydia trachomatis Infection: A Case-Control Study at the Sexually Transmitted Infection Outpatient Clinic in Amsterdam, The Netherlands. Clin Infect Dis. 2017;64(1):24-31.
14. Turner AN, Morrison CS, Munjoma MW, Moyo P, Chipato T, van de Wijgert JH. Vaginal practices of HIV-negative Zimbabwean women. Infect Dis Obstet Gynecol. 2010;2010.
15. Ness RB, Hillier SL, Kip KE, Richter HE, Soper DE, Stamm CA, et al. Douching, pelvic inflammatory disease, and incident gonococcal and chlamydial genital infection in a cohort of high-risk women. Am J Epidemiol. 2005;161(2):186-95.
16. Ott MA, Ofner S, Fortenberry JD. Beyond douching: use of feminine hygiene products and STI risk among young women. J Sex Med. 2009;6(5):1335-40.
17. van der Helm JJ, Bom RJ, Grunberg AW, Bruisten SM, Schim van der Loeff MF, Sabajo LO, et al. Urogenital Chlamydia trachomatis infections among ethnic groups in Paramaribo, Suriname; determinants and ethnic sexual mixing patterns. PLoS One. 2013;8(7):e68698.
18. van 't Klooster C, Haabo V, Ruysschaert S, Vossen T, van Andel TR. Herbal bathing: an analysis of variation in plant use among Saramaccan and Aucan Maroons in Suriname. J Ethnobiol Ethnomed. 2018;14(1):20.
19. van der Helm JJ, Sabajo LO, Grunberg AW, Morre SA, Speksnijder AG, de Vries HJ. Point-of-care test for detection of urogenital chlamydia in women shows low sensitivity. A performance evaluation study in two clinics in Suriname. PLoS One. 2012;7(2):e32122.
20. Bom RJ, van der Helm JJ, Bruisten SM, Grunberg AW, Sabajo LO, Schim van der Loeff MF, et al. The role of Surinamese migrants in the transmission of Chlamydia trachomatis between Paramaribo, Suriname and Amsterdam, The Netherlands. PLoS One. 2013;8(11):e77977.
21. van Melle A, Parriault MC, Basurko C, Jolivet A, Flamand C, Pigeon P, et al. Knowledge, attitudes, behaviors, and practices differences regarding HIV in populations living along the Maroni river:

For peer review only - http://bmjopen.bmj.com/site/about/guidelines.xhtml

particularities of operational interest for Amerindian and Maroon populations. AIDS Care. 2015;27(9):1112-7.
22. Heng LS, Yatsuya H, Morita S, Sakamoto J. Vaginal douching in Cambodian women: its prevalence and association with vaginal candidiasis. J Epidemiol. 2010;20(1):70-6.
23. Li J, Jiang N, Yue X, Gong X. Vaginal douching and sexually transmitted infections among female sex workers: a cross-sectional study in three provinces in China. Int J STD AIDS. 2015;26(6):420-7.
24. Carter M, Gallo M, Anderson C, Snead MC, Wiener J, Bailey A, et al. Intravaginal cleansing among women attending a sexually transmitted infection clinic in Kingston, Jamaica. West Indian Med J. 2013;62(1):56-61.
25. Hickey RJ, Zhou X, Pierson JD, Ravel J, Forney LJ. Understanding vaginal microbiome complexity from an ecological perspective. Transl Res. 2012;160(4):267-82.
26. Hutchinson KB, Kip KE, Ness RB, Gynecologic Infection Follow-Through I. Vaginal douching and development of bacterial vaginosis among women with normal and abnormal vaginal microflora. Sex Transm Dis. 2007;34(9):671-5.
27. McClelland RS, Richardson BA, Graham SM, Masese LN, Gitau R, Lavreys L, et al. A prospective study of risk factors for bacterial vaginosis in HIV-1-seronegative African women. Sex Transm Dis. 2008;35(6):617-23.
28. Veer CV, Bruisten S, Houdt RV, editors. O04.2 Effects of over-the-counter lactic acid-containing vaginal douching products on the vaginal microbiota. Sex Transm Infect; 2017.
29. Klebanoff MA, Andrews WW, Yu KF, Brotman RM, Nansel TR, Zhang J, et al. A pilot study of vaginal flora changes with randomization to cessation of douching. Sex Transm Dis. 2006;33(10):610-3.
30. Sivapalasingam S, McClelland RS, Ravel J, Ahmed A, Cleland CM, Gajer P, et al. An effective intervention to reduce intravaginal practices among HIV-1 uninfected Kenyan women. AIDS Res Hum Retroviruses. 2014;30(11):1046-54.
31. Ravel J, Gajer P, Abdo Z, Schneider GM, Koenig SS, McCulle SL, et al. Vaginal microbiome of reproductive-age women. Proc Natl Acad Sci U S A. 2011;108 Suppl 1:4680-7.
32. Fettweis JM, Brooks JP, Serrano MG, Sheth NU, Girerd PH, Edwards DJ, et al. Differences in vaginal microbiome in African American women versus women of European ancestry. Microbiology. 2014;160(Pt 10):2272-82.
33. Terborg JRH. Sexual behaviour and sexually transmitted diseases among Saramaka and Ndjuka Maroons in the hinterland of Suriname. Paramaribo: MZ Primary Health Care Suriname : Pro-Health; 2001. v, 83 p. p.

For peer review only

For peer review only - http://bmjopen.bmj.com/site/about/guidelines.xhtml

Table 1. Epidemiological characteristics of Creole, Hindustani, Javanese, Maroon and Mixed descent women (n=1040) recruited at the Family planning clinic and STI clinic, Paramaribo, Suriname, 2009-2010.

		Creole	Hindustani	Javanese	Maroon	Mixed descent	P value
		(N=278)	(N=256)	(N=163)	(N=138)	(N=205)	
		n (%)	n (%)	n (%)	n (%)	n (%)	
**Demographic characteristics**							
Recruitment site							<0.001
	Family planning clinic	199 (71.6)	226 (88.3)	146 (89.6)	85 (61.6)	138 (67.3)	
	STI clinic	79 (28.4)	30 (11.7)	17 (10.4)	53 (38.4)	67 (32.7)	
Median age in years [IQR]		29 [24-35]	31 [26-39]	30 [25-36]	28 [23-34]	27 [23-33]	<0.001
Age in years	<25	81 (29.1)	44 (17.2)	39 (23.9)	48 (34.8)	69 (33.7)	0.001
	25-29	64 (23.0)	63 (24.6)	41 (25.2)	29 (21.0)	53 (25.9)	
	30-34	60 (21.6)	50 (19.5)	33 (20.2)	30 (21.7)	40 (19.5)	
	≥35	73 (26.3)	99 (38.7)	50 (30.7)	31 (22.5)	43 (21.0)	

For peer review only - http://bmjopen.bmj.com/site/about/guidelines.xhtml

Education							<0.001
	Low	83 (29.9)	103 (40.2)	51 (31.3)	78 (56.5)	40 (19.5)	
	Medium	147 (52.9)	130 (50.8)	89 (54.6)	41 (29.7)	109 (53.2)	
	High	43 (15.5)	21 (8.2)	21 (12.9)	9 (6.5)	52 (25.4)	
	Unknown	5 (1.8)	2 (0.8)	2 (1.2)	10 (7.2)	4 (2.0)	
**Symptoms**							
Any symptoms		204 (73.4)	181 (70.7)	120 (73.6)	117 (84.8)	140 (68.3)	0.012
Dysuria[a]		60 (21.8)	80 (31.2)	39 (23.9)	46 (33.6)	42 (20.6)	0.008
Dyspareunia** [b]		66 (24.3)	73 (29.0)	41 (25.2)	41 (31.1)	51 (24.9)	0.506
Change in fluor/vaginal discharge[c]		149 (54.0)	126 (49.2)	92 (56.4)	79 (57.7)	110 (53.7)	0.488
Irregular menstruation [d]		81 (29.3)	71 (28.1)	32 (19.9)	51 (38.1)	52 (25.7)	0.012
Abdominal pain[e]		106 (39.8)	106 (41.9)	67 (42.1)	74 (54.4)	78 (38.6)	0.041
**Vaginal hygiene**							

For peer review only - http://bmjopen.bmj.com/site/about/guidelines.xhtml

Use of vaginal practices		109 (39.2)	51 (19.9)	29 (17.8)	112 (81.2)	78 (38.0)	<0.001
Vaginal practices[f]	Water	35 (12.6)	28 (10.9)	17 (10.4)	29 (21.0)	31 (15.1)	0.039
	Herbs	70 (25.2)	9 (3.5)	8 (4.9)	95 (68.8)	29 (14.1)	<0.001
	Lactacyd®	9 (3.2)	5 (2.0)	1 (0.6)	2 (1.4)	14 (6.8)	0.003
	Vinegar	9 (3.2)	2 (0.8)	4 (2.5)	2 (1.4)	9 (4.4)	0.118
	Dettol®	4 (1.4)	4 (1.6)	0 (0.0)	0 (0.0)	0 (0.0)	0.105
	Other[g]	10 (3.6)	7 (2.7)	2 (1.2)	1 (0.7)	4 (2.0)	0.314
Frequency of vaginal practices[h] #							0.027
	Daily	36 (43.9)	11 (32.4)	7 (41.2)	53 (63.1)	25 (39.7)	
	Once a week	18 (22.0)	12 (35.3)	3 (17.6)	19 (22.6)	11 (17.5)	
	Once a month	19 (23.2)	7 (20.6)	5 (29.4)	7 (8.3)	20 (31.7)	
	Less than once a month	9 (11.0)	4 (11.8)	2 (11.8)	5 (6.0)	7 (11.1)	
Reason vaginal use of herbs[f,i] ##	Hygiene	50 (71.4)	4 (44.4)	5 (62.5)	49 (51.6)	16 (55.2)	0.107
	Sexual	24 (34.3)	0 (0.0)	1 (12.5)	46 (48.4)	11 (37.9)	0.0150.485
	Health	11 (15.7)	0 (0.0)	1 (12.5)	16 (16.8)	2 (6.9)	-*
	Other	3 (4.3)	0 (0.0)	0 (0.0)	6 (6.3)	0 (0.0)	

For peer review only - http://bmjopen.bmj.com/site/about/guidelines.xhtml

Last vaginal use of herbs[j] ##							-*
	Today	7 (12.7)	0 (0.0)	0 (0.0)	19 (26.4)	1 (5.9)	
	Yesterday	5 (9.1)	0 (0.0)	0 (0.0)	23 (31.9)	1 (5.9)	
	Last week	20 (36.4)	0 (0.0)	2 (33.3)	15 (20.8)	4 (23.5)	
	More than a week ago	23 (41.8)	4 (100.0)	4 (66.7)	15 (20.8)	11 (64.7)	
**Sexual behaviour**							
Number of partners in the preceding month[k]							<0.001
	0	17 (6.2)	8 (3.1)	2 (1.2)	10 (7.5)	11 (5.7)	
	1	237 (87.1)	236 (92.5)	148 (91.4)	117 (87.3)	158 (82.3)	
	2	16 (5.9)	6 (2.4)	11 (6.8)	7 (5.2)	10 (5.2)	
	>2	2 (0.7)	5 (2.0)	1 (0.6)	0 (0.0)	13 (6.8)	
Condom use during sex[l]							<0.001
	Always	49 (18.0)	17 (6.7)	10 (6.2)	23 (16.8)	37 (18.0)	
	Never or inconsistent	223 (82.0)	236 (93.3)	152 (93.8)	114 (83.2)	168 (82.0)	

For peer review only - http://bmjopen.bmj.com/site/about/guidelines.xhtml

Number of partners in the preceding 12 months							<0.001
	0	8 (2.9)	12 (4.7)	5 (3.1)	11 (8.0)	11 (5.4)	
	1	208 (74.8)	209 (81.6)	123 (75.5)	82 (59.4)	127 (62.0)	
	2	35 (12.6)	27 (10.5)	20 (12.3)	33 (23.9)	35 (17.1)	
	>2	27 (9.7)	8 (3.1)	15 (9.2)	12 (8.7)	32 (15.6)	
Ethnic sexual mixing	Reported ≥ 1 sexual partner from another ethnic group	89 (32.0)	45 (17.6)	80 (49.1)	43 (31.2)	123 (60.0)	<0.001
Sex in exchange for money or goods[b]		7 (2.6)	6 (2.4)	0 (0.0)	5 (3.7)	19 (9.4)	<0.001
Chlamydia trachomatis infection ###		37 (13.3)	16 (6.2)	25 (15.3)	15 (10.9)	29 (14.1)	0.022

a: 5 missings, b: 16 missings, c: 3 missings, d: 14 missings, e:24 missings, f: multiple options could be chosen, g: other vaginal practices are products like lactacyd but from other brands; soap, anti fungal and eggs., h: 99 missings, i: 56 missings, % of those who filled in the question, j: 57 missings, k: 25 missings, l: 11 missings.

*: *p* values could not obtained due to low numbers. IQR = interquartile range.

** pain during sexual intercourse

the denominator for the percentages is the group of women who indicated they did vaginal practices.

the denominator for the percentages is the group of women who indicated they used vaginal herbs.

as diagnosed by nucleic acid amplification test

For peer review only

For peer review only - http://bmjopen.bmj.com/site/about/guidelines.xhtml

Table 2. Univariable and multivariable logistic regression analyses of determinants associated with vaginal herb use among Creole and Maroon women, Paramaribo, Suriname, 2009-2010.

		Use of vaginal herbs	Univariable OR (95% CI)	*P* value	Multivariable Adjusted OR (95% CI)*	*P* value
		n/N (%)				
**Demographic characteristics**						
Ethnic groups	Creole	70/278 (25.2)	1	<0.001	1	<0.001
	Maroon	95/138 (68.8)	6.57 (4.18-10.30)		5.33 (3.26-8.71)	
Recruitment site	Family planning clinic	96/284 (33.8)	1	<0.001	1	0.005
	STI clinic	69/132 (52.3)	2.15 (1.41-3.27)		2.04 (1.24-3.36)	
Age in years				0.068		0.067
	<25	59/129 (45.7)	1.99 (1.15-3.42)		2.23 (1.17-4.27)	
	25-29	35/93 (37.6)	1.42 (0.79-2.57)		1.78 (0.88-3.57)	
	30-34	40/90 (44.4)	1.88 (1.04-3.40)		2.23 (1.13-4.42)	
	≥35	31/104 (29.8)	1		1	
Education[a]				<0.001		0.002
	Low	88/161 (54.7)	5.06 (2.38-10.79)		3.80 (1.68-8.57)	
	Medium	59/188 (31.4)	1.92 (0.90-4.09)		2.02 (0.90-4.51)	
	High	10/52 (19.2)	1		1	

For peer review only - http://bmjopen.bmj.com/site/about/guidelines.xhtml

**Sexual behaviour**				
Number partners preceding month[b]				0.106
	0	10/27 (37.0)	1	
	1	135/354 (38.1)	1.05 (0.47-2.36)	
	≥2	15/25 (60.0)	2.55 (0.83-7.80)	
Number partners preceding 12 months				0.216
	0	9/19 (47.4)	1	
	1	107/290 (36.9)	0.65 (0.26-1.65)	
	≥2	49/107 (45.8)	0.94 (0.35-2.50)	
Ethnic sexual mixing	No	115/284 (40.5)	1	0.612
	Yes	50/132 (37.9)	0.90 (0.59-1.37)	
Sex in exchange for money or goods[c]	No	155/396 (39.1)	1	0.191
	Yes	7/12 (58.3)	2.17 (0.68-6.98)	
**Symptoms**				
Any symptoms	No	24/95 (25.3)	1	0.001
	Yes	141/321 (43.9)	2.32 (1.39-3.87)	
Dysuria[d]	No	114/306 (37.3)	1	0.104
	Yes	49/106 (46.2)	1.45 (0.93-2.26)	

For peer review only - http://bmjopen.bmj.com/site/about/guidelines.xhtml

Dyspareunia (pain during sexual inter-course)[e]	No<br>Yes	113/297 (38.0)<br>47/107 (43.9)	1<br>1.28 (0.82-2.00)	0.287
Change in fluor/vaginal discharge[f]	No<br>Yes	71/185 (38.4)<br>93/228 (40.8)	1<br>1.11 (0.74-1.65)	0.619
Irregular menstruation[g]	No<br>Yes	102/278 (36.7)<br>59/132 (44.7)	1<br>1.40 (0.92-2.13)	0.122
Abdominal pain[h]	No<br>Yes	78/222 (35.1)<br>83/180 (46.1)	1<br>1.58 (1.06-2.36)	0.026

*ORs in the multivariable model are adjusted for all factors for which adjusted ORs are shown. In the final model 401 participants were included.
a:15 missings, b: 10 missings, c: 8 missings, d: 4 missings , e: 12 missings, f: 3 missings, g: 6 missings, h: 14 missings

For peer review only - http://bmjopen.bmj.com/site/about/guidelines.xhtml

Table 3. Vaginal products mentioned among female participants using vaginal practices Paramaribo, Suriname, 2009-2010.

Vaginal practice (scientific names if applicable)	Maroon ethnicity (N=112)	Creole ethnicity (N=109)	Other ethnicities[a] (N=158)
Almond leaves (*Terminalia catappa*)	1	3	1
Andoya (*Campomanesia aromatica*)	6	1	1
Blaka masusa	0	1	0
Blaka uma	0	5	2
Broko pie (*Bellucia grossularioides*)	1	1	0
Dettol®	0	4	4
Djamu (*Syzygium cumini*)	9	4	0
Douchegel	0	0	1
Dram (alcohol)	0	1	0
Eggs	0	1	0
Eva products	0	1	0
Faya lobi (*Ixora coccinea)*	2	0	0
Feififingawiwiri	1	1	0
Guave (*Psidium guajava)*	3	2	0
Intimate wash products	0	3	5
Kill somebody (*Dimorphandra conjugata)*	13	4	1
Lactacyd®	2	9	20
Manjablad (Mango leaves)	4	9	3
Odany jewa	0	0	3
Paraklem	4	1	0
Pedreku	1	1	2
Pikin bë (*Vismia* Vand. spp)	7	5	14
Redikatun (*Gossypium barbadense*)	4	16	6
Suku trobi	7	3	0
Twigs	0	0	1
Umaanesi	4	1	2
Unknown leaves	13	6	6
Vagisil	0	1	0
Vinegar	2	9	15

For peer review only - http://bmjopen.bmj.com/site/about/guidelines.xhtml

Wasduku (*Clidemia capitellata*)	9	1	3
Water	29	35	76
Yarakopie	2	3	1
Yoghurt	0	0	1

a: Hindustani, Javanese and Mixed race ethnicity

For peer review only

Table 4. Univariable and multivariable logistic regression analyses of variables associated with cervicovaginal *C. trachomatis* infection among Creole and Maroon women, Paramaribo, Suriname, 2009-2010.

		NAAT positive	Univariable OR (95% CI)	*P* value	Multivariable Adjusted OR (95% CI)*	*P* value
		n/N (%)				
**Demographic characteristics**						
Ethnic group	Creole	37/278 (13.3)	1	0.479		
	Maroon	15/138 (10.9)	0.79 (0.42-1.50)			
Recruitment site	Family planning clinic	25/284 (8.8)	1	0.001	1	0.003
	STI clinic	27/132 (20.5)	2.66 (1.48-4.80)		2.59 (1.39-4.83)	
Age in years				0.001		0.002
	<25	25/129 (19.4)	4.76 (1.75-12.92)		4.37 (1.59-12.00)	
	25-29	17/93 (18.3)	4.43 (1.56-12.54)		4.96 (1.72-14.26)	
	30-34	5/90 (5.6)	1.17 (0.33-4.16)		1.20 (0.33-4.35)	
	≥35	5/104 (4.8)	1		1	

For peer review only - http://bmjopen.bmj.com/site/about/guidelines.xhtml

Education				0.444		
	Low	19/161 (11.8)	1.03 (0.39-2.73)			
	Medium	23/188 (12.2)	1.07 (0.41-2.78)			
	High	6/52 (11.5)	1			
	Unknown	4/15 (26.7)	2.79 (0.67-11.60)			
**Vaginal hygiene**						
Use of vaginal practices	No	21/195 (10.8)	1	0.317		
	Yes	31/221 (14.0)	1.35 (0.75-2.44)			
Herb use	No	27/251 (10.8)	1	0.187	1	0.564
	Yes	25/165 (15.2)	1.48 (0.83-2.66)		1.20 (0.65-2.22)	
Frequency of the use of vaginal practices[b]	Daily	16/89 (18.0)	1.82 (0.90-3.68)	0.282		
	At least once a week	7/36 (19.4)	2.00 (0.78-513)			
	At least once a month	2/25 (8.0)	0.72 (0.16-3.28)			
	Less then once a month	3/14 (21.4)	2.26 (0.58-8.76)			
	Never	21/195 (10.8)	1			
**Sexual behaviour**						
Number of partners preceding month[c]	0-1	43/381 (11.3)	1	0.018		
	≥2	7/25 (28.0)	3.06 (1.21-7.74)			

For peer review only - http://bmjopen.bmj.com/site/about/guidelines.xhtml

Condom use[d]	Always	11/72 (15.3)	1.30 (0.63-2.68)	0.473
	Never or inconsistent	41/337 (12.2)	1	
Number of partners in the preceding 12 months	0-1	37/309 12.0)	1	0.582
	≥2	15/107 (14.0)	1.20 (0.63-2.28)	
Ethnic sexual mixing	Reported only sexual partners from own ethnic group	32/284 (11.3)	1	0.267
	Reported at least one sexual partner from another ethnic group	20/132 (15.2)	1.41 (0.77-2.57)	
Sex in exchange for money or goods[e]	No	47/396 (11.9)	1	0.038
	Yes	4/12 (33.3)	3.71 (1.08-12.81)	

*ORs in the multivariable model are adjusted for all factors for which adjusted ORs are shown. In the final model 416 participants were included.
Footnote: NA: not applicable.
, b: 57 missings, c: 10 missings, d: 7 missings, e: 8 missings,

For peer review only - http://bmjopen.bmj.com/site/about/guidelines.xhtml

STROBE Statement—Checklist of items that should be included in reports of ***cross-sectional studies***

	Item No	Recommendation	Reported on page #
**Title and abstract**	1	(*a*) Indicate the study's design with a commonly used term in the title or the abstract	1
		(*b*) Provide in the abstract an informative and balanced summary of what was done and what was found	2
**Introduction**			
Background/rationale	2	Explain the scientific background and rationale for the investigation being reported	4
Objectives	3	State specific objectives, including any prespecified hypotheses	4/5
**Methods**			
Study design	4	Present key elements of study design early in the paper	5
Setting	5	Describe the setting, locations, and relevant dates, including periods of recruitment, exposure, follow-up, and data collection	5
Participants	6	(*a*) Give the eligibility criteria, and the sources and methods of selection of participants	5
Variables	7	Clearly define all outcomes, exposures, predictors, potential confounders, and effect modifiers. Give diagnostic criteria, if applicable	5/6
Data sources/ measurement	8*	For each variable of interest, give sources of data and details of methods of assessment (measurement). Describe comparability of assessment methods if there is more than one group	*6*
Bias	9	Describe any efforts to address potential sources of bias	5/6
Study size	10	Explain how the study size was arrived at	Ref 19
Quantitative variables	11	Explain how quantitative variables were handled in the analyses. If applicable, describe which groupings were chosen and why	6
Statistical methods	12	(*a*) Describe all statistical methods, including those used to control for confounding	6
		(*b*) Describe any methods used to examine subgroups and interactions	6
		(*c*) Explain how missing data were addressed	15
		(*d*) If applicable, describe analytical methods taking account of sampling strategy	n.a.
		(*e*) Describe any sensitivity analyses	6
**Results**			

For peer review only

For peer review only - http://bmjopen.bmj.com/site/about/guidelines.xhtml

Participants	13*	(a) Report numbers of individuals at each stage of study—eg numbers potentially eligible, examined for eligibility, confirmed eligible, included in the study, completing follow-up, and analysed	6
		(b) Give reasons for non-participation at each stage	n.a.
		(c) Consider use of a flow diagram	n.a.
Descriptive data	14*	(a) Give characteristics of study participants (eg demographic, clinical, social) and information on exposures and potential confounders	6/13
		(b) Indicate number of participants with missing data for each variable of interest	15
Outcome data	15*	Report numbers of outcome events or summary measures	7/16/20
Main results	16	(*a*) Give unadjusted estimates and, if applicable, confounder-adjusted estimates and their precision (eg, 95% confidence interval). Make clear which confounders were adjusted for and why they were included	16/20
		(*b*) Report category boundaries when continuous variables were categorized	13-21
		(*c*) If relevant, consider translating estimates of relative risk into absolute risk for a meaningful time period	n.a.
Other analyses	17	Report other analyses done—eg analyses of subgroups and interactions, and sensitivity analyses	n.a.
**Discussion**			
Key results	18	Summarise key results with reference to study objectives	8
Limitations	19	Discuss limitations of the study, taking into account sources of potential bias or imprecision. Discuss both direction and magnitude of any potential bias	8
Interpretation	20	Give a cautious overall interpretation of results considering objectives, limitations, multiplicity of analyses, results from similar studies, and other relevant evidence	8/9
Generalisability	21	Discuss the generalisability (external validity) of the study results	9
**Other information**			
Funding	22	Give the source of funding and the role of the funders for the present study and, if applicable, for the original study on which the present article is based	1

*Give information separately for exposed and unexposed groups.

Note: An Explanation and Elaboration article discusses each checklist item and gives methodological background and published examples of transparent reporting. The STROBE checklist is best used in conjunction with this article (freely available on the Web sites of PLoS Medicine at http://www.plosmedicine.org/, Annals of Internal Medicine at

For peer review only - http://bmjopen.bmj.com/site/about/guidelines.xhtml

http://www.annals.org/, and Epidemiology at http://www.epidem.com/). Information on the STROBE Initiative is available at www.strobe-statement.org.

For peer review only

For peer review only - http://bmjopen.bmj.com/site/about/guidelines.xhtml

BMJ Open

Vaginal herb use and Chlamydia trachomatis infection: a cross-sectional study among women of various ethnic groups in Suriname

Journal:	*BMJ Open*
Manuscript ID	bmjopen-2018-025417.R1
Article Type:	Research
Date Submitted by the Author:	14-Dec-2018
Complete List of Authors:	Van der Helm, Jannie; Public Health Service Amsterdam, Department of Research<br>Schim van der Loeff, Maarten; GGD Amsterdam<br>de Vries, Esther; Public Health Service Amsterdam, Department of Research<br>van der Veer, Charlotte; GGD Amsterdam, Streeklaboratorium<br>Grünberg, Antoon; Ministry of Health, Paramaribo, Suriname, Department of Public Health<br>Mans, Dennis; Department of Pharmacology<br>de Vries, H; University of Amsterdam, Department of Dermatology
**Primary Subject Heading**:	Sexual health
Secondary Subject Heading:	Global health, Infectious diseases, Obstetrics and gynaecology, Public health, Epidemiology
Keywords:	Suriname, ethnicity, vaginal, vaginal douching, herb therapy

SCHOLARONE™
Manuscripts

Vaginal herb use and *Chlamydia trachomatis* infection: a cross-sectional study among women of various ethnic groups in Suriname

Running title: Vaginal herbal use and chlamydia in Suriname

Jannie J. van der Helm PhD[1], Maarten F. Schim van der Loeff MD, PhD[2,5,], Esther de Vries, MSc[2], Charlotte van der Veer[2], Antoon W. Grünberg MD, PhD[3], and Dennis R. Mans PhD[4], Henry J.C. de Vries MD, PhD[2,5]

1. Centre for Environmental Safety and Security, National Institute of Public Health and the Environment, Bilthoven
2. Public Health Service (GGD) Amsterdam, Department of Infectious diseases, Amsterdam, the Netherlands
3. Department of Public Health, Ministry of Health, Paramaribo, Suriname
4. Department of Pharmacology, Faculty of Medical Sciences, Anton de Kom University of Suriname, Paramaribo, Suriname
5. Amsterdam UMC, University of Amsterdam, department of dermatology, Amsterdam Institute for Infection and Immunity (AI&II), Meibergdreef 9, Amsterdam, Netherlands

Corresponding author:
Henry John Christiaan de Vries
Department of Dermatology, Academic Medical Center, University of Amsterdam
Meibergdreef 9, 1105 AZ Amsterdam-Zuidoost, the Netherlands,
Phone: +31 (0)20 566 9111, Fax: +31 (0)20 566 4440 email: h.j.devries@amc.nl

Word count of the abstract: 242
Word count of the text: 2872
Number of references: 33
Number of tables: 4
Number of figures: 0

Conflict of Interest: None reported

Funding: This work was supported by the Research and Development fund of the Public Health Service of Amsterdam [project no 2369 and 2371] and AGIS healthcare insurance [RVVZ no 1417000]. The funders had no role in study design, data collection and analysis, decision to publish, or preparation of the manuscript.

Contributorship statement: JvdH collected the data, analysed the data and wrote the manuscript; MSvdL supervised the analysis and wrote the manuscript; EdV analysed the data and wrote the first manuscript draft; CvdV wrote the manuscript; AWG designed the study, collected data and

For peer review only - http://bmjopen.bmj.com/site/about/guidelines.xhtml

wrote the manuscript; DRM collected the data and wrote the manuscript; and HdV designed the study, and wrote the manuscript.

Abstract

Objective: Vaginal steam baths with herb leaves (herb use) is practiced by some Surinamese women. We assessed herb use among women from the five most prevalent ethnic groups, and if herb use is associated with *Chlamydia trachomatis* infection.

Setting: Participants were recruited at a sexually transmitted infection clinic (STI) and a family planning clinic (FP) in Paramaribo, Suriname.

Participants: 1040 women were included subsequently, comprising the following ethnic groups: Creole (26.7%), Hindustani (24.6%), Javanese (15.7%), Maroon (13.3%) and Mixed descent (19.7%).

Methods: Nurses collected a questionnaire and vaginal swabs for nucleic acid amplification *C. trachomatis* testing.

Primary outcomes: Determinants of vaginal herb use and *C. trachomatis* infection via univariable and multivariable logistic regression.

Results: Herb use was most common among Maroon (68.8%) and Creole women (25.2%). In multivariable analysis including only Maroon and Creole women, determinants significantly associated with vaginal herb use were (odds ratio; 95%CI): Maroon ethnic descent (5.33; 3.26-8.71 versus Creole), recruitment at the STI clinic (2.04;1.24-3.36 versus FP), lower education levels (3.80;1.68-8.57 lower versus higher, and 2.02;0.90-4.51 middle versus higher). Lower age and recruitment at the STI clinic *were associated with C. trachomatis* infection, but not vaginal herb use.

Conclusion: In Suriname, vaginal herb use is common among Maroon and Creole women. Education, ethnic group and recruitment site were determinants for herb use. Vaginal herb use was not a determinant of *C. trachomatis* infection. Future research should focus on the effect of herb use on the vaginal microbiome and mucosal barrier.

For peer review only - http://bmjopen.bmj.com/site/about/guidelines.xhtml

Strengths and limitations of this study:

- This is the first study into the relationship between vaginal herb use and a sexually transmitted infection in Suriname.
- Moreover, we show that education, ethnic group and recruitment site (STI outpatient clinic vs Family Planning clinic) are determinants for vaginal herb use.
- Lastly we show that vaginal herb use is not a determinant of C. trachomatis infection.
- We did not assess if vaginal herbs were used intra-vaginally and/or externally.
- We did not study the effect of vaginal herb use on the vaginal microbiota.

Keywords: herb therapy; vaginal douching; vaginal; ethnicity; Suriname

Data sharing statement: Deidentified participant data are available upon request through the corresponding author Henry J C de Vries. ORCID identifier 0000-0001-9784-547X

For peer review only - http://bmjopen.bmj.com/site/about/guidelines.xhtml

Introduction

Depending on cultural habits, women worldwide engage in a variety of vaginal hygiene practices, such as the use of intra-vaginal douches, herbal vaginal steam baths, or the direct insertion of herbs into the vagina.(1-4) Studies in the United States of America showed that women who had less income, less education, were unmarried, lived in the southern States, and were of African-American descent were more likely to engage in such practices when compared to white women.(2)

Vaginal practices are not only intended for feeling clean and fresh, getting rid of vaginal malodour, or removing residual menstrual blood(5, 6), but also for improving the appearance of the vagina and for enhancing sensation during intercourse, and securing the relationship with, and economic support by the male partner.(2) However, these practices may increase the risk of acquiring infections, such as HIV(4), HPV(7), but also bacterial sexually transmitted infections (STI) and trichmoniasis.(8) Particularly intra-vaginal practices, such as use of water and soap, the insertion of a cloth or a piece of paper into the vagina, to dry and tighten the vagina was associated with acquiring HIV.(4) An underlying mechanism for this may be that intra-vaginal practices deplete the relative amount of vaginal *Lactobacillus spp.*(4, 9) Lactobacilli produce anti-microbials and acidify the vagina by lactic-acid production and are thus considered to be a hallmark for vaginal health; their depletion is known to mediate HIV and STI acquisition.(4, 10-13)

Risk for STI may be further increased by a breakdown of the vaginal epithelial barrier and sometimes even the occurrence of lacerations.(3) In addition, a lack of vaginal fluids increases the risk of condom rupture. Although several studies have reported an association between vaginal practices and STI(1, 8, 14), other studies did not.(15, 16) Unfortunately earlier studies did not consider the type of vaginal practice nor the frequency of their use.

Suriname is located on the northeast coast of South America, bordering the Atlantic Ocean to the north and surrounded by French Guiana to the east, Brazil to the south, and Guyana to the west. The population of approximately 570,000 is characterized by the ethnical and cultural diversity. The largest ethnic groups are the Creoles and Maroons (both originating from enslaved Africans imported in the 17th to 19th century), and the Hindustani and Javanese (descendants from indentured laborers from the former British Indies and Dutch Indies, respectively, arrived around the turn of the 20th century). There are, in addition, Indigenous Amerindians, people of Mixed descent, and descendants from Chinese laborers, European colonists, and immigrants from various Latin American and Caribbean countries such as Brazil, Guyana, French Guiana, Haiti, and Cuba.(17)

Among these ethnic groups, Afro-Surinamese women (Creoles and Maroons) in particular engage in a variety of vaginal practices including vaginal steam baths containing certain herbs for drying and tightening the vagina, cleansing after menstruation or after birth to prevent puerperal fever, 'placing the uterus back into position', or preventing a flabby abdominal wall.(3) Typically, for a herbal genital steam bath, a woman sits with spread legs on a bucket or bidet containing warm water with certain herbs to steam her inner genital parts. Depending on the herbs, the cooled down bath is used to wash the genital parts after steaming.(18) For direct internal application, Dettol® (the brand name of the antiseptic chloroxylenol), yoghurt, lemon juice, vinegar as well as commercial products such as

For peer review only - http://bmjopen.bmj.com/site/about/guidelines.xhtml

effervescent tablets, gels, or emulsions are used. Commercial products in the form of wipes, emulsions, crèmes, and mousses can also be used externally.(3,18)

In this study we examined the type and frequency of vaginal practices in women visiting an STI outpatient clinic and a family planning (FP) clinic, in particular the use of herbs and the reasons for their use were identified. Moreover, we assessed factors associated with vaginal herb use, and examined whether vaginal herb use is a determinant of cervicovaginal *C. trachomatis* infection.

Methods

Ethics statement

The data used for the current study were gathered in the course of a larger study concerning chlamydia, the Urogenital Chlamydia Rapid Test Evaluation in Paramaribo and Amsterdam (CUSTEPA) study.(17, 19, 20) The study was approved by the Ethics Committees of the Ministry of Health of the Republic of Suriname (VG010-2007) and the Academic Medical Center, University of Amsterdam, The Netherlands (MEC07/127). Women participated after given written informed consent.

Study population

Recruitment took place in Suriname's capital city Paramaribo in the period 2009-2010 at two locations: the Dermatological Service, an integrated outpatient STI clinic that offers free-of-charge examination and treatment of STIs and infectious skin diseases such as leprosy and leishmaniasis; and the Lobi Foundation, a FP clinic. The study was cross-sectional, and each participant was given a unique code. Subsequently, a nurse interviewed the participant about demographic characteristics, including self-reported ethnic background, sexual behaviour, symptoms, STI history, and vaginal hygiene. Criteria for exclusion were age below 18 years and previous participation within the CUSTEPA study.

Patient and Public Involvement

Representatives of the Maroon population were consulted on the outcome of the study.

Vaginal hygiene practices

Using a structured questionnaire, nurses asked women in detail about the use of products for vaginal practices such as douches, herbs, or other home-made products, and if so, at which frequency. The questionnaires included several options such as the use of herbal genital steam baths, vinegar, water, yoghurt, and two often used commercial products: Lactacyd® (Omega Pharma Nederland BV, Rotterdam, The Netherlands) that consists of lactic acid-containing wipes, crèmes, emulsion, and mousses, and Dettol® (Reckitt Benckiser Group plc, Slough, UK). Participants could specify the products they used. Pre-coded answer options for the frequency of vaginal practices were: daily, weekly, monthly, or less than once a month. Women who used vaginal herbs were asked about their most recent application, and the reasons for use (hygiene, sexual pleasure, health or other).

For peer review only - http://bmjopen.bmj.com/site/about/guidelines.xhtml

Items mentioned under reason "other" that fitted one of the 3 former reasons were regrouped accordingly.

Specimen collection and testing procedures

Vaginal swabs were collected by trained nurses, stored and shipped to the Public Health Laboratory in Amsterdam, as described earlier.(19) Here, the samples were tested for *C. trachomatis* rRNA (APTIMA CT, Hologic Gen-Probe Inc, San Diego, USA) according to the manufacturer's instructions. Test results were sent to the clinics in Suriname, and participants (and partners if indicated) were managed as described earlier.(19)

Statistical analysis

To examine whether epidemiological characteristics and behaviour, including the use and frequency of vaginal practices, differed between women from the five major ethnic groups, characteristics and behaviours of these groups were compared using the χ^2-test; age was compared using the Kruskal-Wallis test. Univariable logistic regression analysis was used to assess determinants for the use of vaginal herbal. In univariable analysis the following variables were assessed: age, education, ethnic group, ethnicity of sexual partner, recruitment site, condom use, number of sexual partners in the preceding one and twelve month(s), having had sex in exchange for money or goods, and vaginal symptoms. Variables with P<0.05 in univariable analysis were entered into multivariable logistic regression models; variables were removed stepwise until only significant variables were retained, but based on earlier studies age was forced into the model.(1, 2). A second logistic regression analysis was conducted to assess whether vaginal herb use was independently associated with *C. trachomatis* infection. Variables univariately associated with *C. trachomatis* infection were entered into a multivariable model, and stepwise removed until only significant variables were retained; vaginal herb use was forced into the model. P values < 0.05 were considered statistically significant. SPSS statistics version 21 (IBM, City, State, USA) was used for the analysis.

Results

Study population

We initially included 1,093 women who self-identified with one of the following ethnic backgrounds: 17 (1.6%) Caucasian, 12 (1.1%) Chinese, 278 (25.4%) Creole, 256 (23.4%) Hindustani, 19 (1.7%) Indigenous, 163 (14.9%) Javanese, 138 (12.6%) Maroon, 205 (18.8%) Mixed descent, and 5 unknown (0.5%). Due to the small numbers we excluded Caucasian, Chinese, Indigenous women, and women with unknown background from further analysis. As a result, the study population included 1,040 women of either Creole, Hindustani, Javanese, Maroon, or Mixed descent (Table 1).

The majority of participants was recruited at the FP clinic (between 61.6% and 89.6% per ethnic background). The median age ranged from 27 years (interquartile range (IQR), 23-33 years) for Mixed descent women to 31 years (IQR, 26-39 years) for Hindustani women (P<0.001). Most women

For peer review only - http://bmjopen.bmj.com/site/about/guidelines.xhtml

from Maroon descent (56.5%) had lower education whereas most of those from the other ethnic backgrounds had at least medium education (P<0.001).

Vaginal symptoms were reported frequently, ranging from 68.3% (Mixed descent women) to 84.8% (Maroon women; P=0.01). In all ethnic groups, more than 80% of women reported one sexual partner during the preceding month, and more than 60% reported one sexual partner during the preceding 12 months. Discordant mixing (intercourse with a partner of a different ethnic group) was most frequently seen among Mixed descent and Javanese women (60.0% and 49.1%, respectively), followed by Creole, Maroon, and Hindustani women. The prevalence of *C. trachomatis* infections was highest in Javanese women (15.3%) followed by Mixed descent women (14.1%) and Creole women (13.3%) (P = 0.02).

Vaginal hygiene

Table 1 shows the ways in which vaginal hygiene was performed within the study population. Vaginal hygiene was most common in Maroon women (81.2%) followed by Creole (39.2%) and Mixed descent women (38.0%). Between 10.4% and 21.0% of women used tap water for vaginal cleansing, with the largest proportion in Maroon (21.0%) and Mixed descent women (15.1%). Lactacyd®, vinegar, and other substances such as Dettol® were used less often.

In those women that performed vaginal hygiene, daily use was reported by Maroons (63.1%), Creoles (43.9%), Javanese (41.2%), Mixed descent (39.7%), and Hindustani (32.4%).

Vaginal herb use

Vaginal herb use was reported most frequent by Maroon women (68.8%), followed by Creole women (25.2%), women of Mixed descent (14.1%), Javanese (4.9%) and Hindustani (3.5%) (Table 1). Hygiene was the most often mentioned reason for vaginal herb use (across all groups ranging from 68.1% to 100%), followed by sexual pleasure (ranging from 0% to 63.9%). Due to low numbers of women reporting vaginal herb use among women of Hindustani, Javanese and Mixed descent, only Maroon and Creole women were included in the further analyses of vaginal herb use.

In univariable analysis, vaginal herb use was significantly associated with recruitment location, education, ethnic background, vaginal symptoms, and abdominal pain (Table 2). Although not significant, younger women reported vaginal herb use more often than women aged 35 years and above. In multivariable analysis, vaginal herb use was significantly associated with recruitment at the STI clinic (OR 2.04; 95%CI 1.24-3.36 versus the FP clinic), lower education levels (OR 3.80; 95%CI 1.68-8.57 lower versus higher education; and OR 2.02; 95%CI 0.90-4.51 medium versus higher education), and Maroon ethnicity (OR, 5.33; 95%CI 3.26-8.71 versus Creole). Although not significant in multivariable analysis, younger age was associated with vaginal herb use.

As described in table 3, a wide variety of herbs were used. Maroon women predominantly used “kill somebody” or “kill your darling *Dimorphandra conjugata* (Splitg.) Sandw. (Fabaceae)” (13), the “jambolan or dyamu *Syzygium cumini* (L.) Skeels (Myrtaceae)” (9), the “towel or wasduku *Clidemia capitellata* (Bonpl.) D.Don (Melastomataceae)” (9), the “guavaberry or andoya *Campomanesia aromatica* (Aubl.) Griseb. (Myrtaceae)” (6), and “pikin bë/witi baka píyjá páu (small red/white backed

For peer review only - http://bmjopen.bmj.com/site/about/guidelines.xhtml

pineapple) *Vismia* Vand. spp. (Hypericaceae)" (7). Creole women mostly mentioned "sea island cotton or redikatun *Gossypium barbadense* L. (Malvaceae)" (16) in addition to the leaves of the "tropical-almond or amandra *Terminalia catappa* L. (Combretaceae)", the "mess apple or broko pi ('broken penis') *Bellucia grossularioides* (L.) Triana (Melastomataceae)", the "guava or guyaba *Psidium guajava* L. (Myrtaceae)", the "jungle geranium or faya lobi ('fiery love') *Ixora coccinea* L. (Rubiaceae)", and the "ant bush or kapasiwiwiri ('herb of the nine-banded armadillo') *Siparuna guianensis* Aubl. (Monimiaceae)".

V*aginal herb use and C. trachomatis infection*

V*aginal* herb use was not associated with *C. trachomatis infection*, neither in univariable nor in multivariable analysis (aOR, 1.20; 95%CI: 0.65-2.22: p=0.564), (Table 4). In contrast, recruitment location, age, and reporting sex in exchange for money or goods, were associated with *C. trachomatis* infection in univariable analysis. In multivariable analysis, chlamydia infection was associated with recruitment at the STI clinic versus FP clinic (OR, 2.59; 95%CI: 1.39-4.83; p=0.003) and also with younger age, both for < 25 years, and 25-29 years versus ≥ 35 years (resp aOR, 4.37; 95%CI 1.59-12.00, aOR 4.96; CI95% 1.72-14.26 and aOR 1.20; CI95% 0.33-4.35; p=0.002).

Discussion

We assessed the use and frequency of various vaginal practices among female STI clinic and FP clinic visitors in Suriname, and found that: (1) vaginal practices are commonly used, and most frequently by Maroon, Creole and Mixed decent women; (2) vaginal herb use is more common among women with lower education; (3) vaginal herb use is most frequently practiced for hygienic reasons; and (4) vaginal herb use is not associated with *C. trachomatis* infection.

Ethnic background was the most important determinant for vaginal herb use. The extensive vaginal use of herbs among Maroon women has previously been described in French Guiana, where a prevalence of 96.1% was found.(21)

Women who experienced vaginal symptoms were more likely to engage in vaginal herb use. Since this is a cross-sectional study, it is not clear if vaginal symptoms are a cause, or an effect of vaginal practices. A Cambodian study also found an association between vaginal douching and vaginal symptoms.(22) Similarly, female sex workers in China were more likely to engage in vaginal practices when having STI-related symptoms, and reportedly engaged more in vaginal douching when experiencing vaginal symptoms.(23) In contrast, a study with Jamaican women who attended a public STI clinic found that vaginal itching led to a lower frequency of vaginal douching.(24) Prospective (intervention) studies could shed light on any casual links between vaginal practices and vaginal symptoms. The high prevalence of vaginal herb use among Maroon women has been previously reported, and it is conceivable that Maroon women do not engage in vaginal herb use as consequence of vaginal symptoms but rather out of (cultural) habit.(21)

For peer review only - http://bmjopen.bmj.com/site/about/guidelines.xhtml

As previously described, hygiene was the most important reason for vaginal herb use, followed by sexual reasons.(3) Moreover, Maroon women mentioned as a reason to make oneself more attractive for one's partner.

We found no association between vaginal herb use and infection with *C. trachomatis*. This confirms earlier findings of the above mentioned Chinese study where no association was found among sex workers between vaginal practices (mostly disinfectants after sex with clients) and STI (syphilis, *Neisseria gonorrhoea* and *C. trachomatis* combined).(23)

This study has some limitations. This study did not assess the mode of vaginal practices, e.g. whether the products were applied intra-vaginally and/or externally. Previous studies have found associations between the internal use of vaginal practices and HIV.(4) Therefore we cannot exclude that the lack of association between vaginal herb use and *C. trachomatis* infection may be explained by predominantly external use of herbs. Moreover we did not study the effect of vaginal herb use on the vaginal microbiota. Lactobacilli generally constitute a healthy vaginal microbiota as they generate an acidic environment (pH 4.0-4.5) and produce antimicrobials, that restrict the growth of most pathogens.(25) A non-lactobacillus-dominated vaginal microbiota is thus considered dysbiotic. Intra-vaginal practices have been linked to developing vaginal dysbiosis(4, 9, 26, 27), but these studies studied douching behaviour in general and did not differentiate between different types of intra-vaginal products. The use of an over-the-counter lactic-acid containing douche was recently studied prospectively among healthy Dutch women and this was not found to significantly impact the vaginal microbiota composition, although an increased odds for having non-lactobacillus dominated vaginal microbiota among users was observed.(28) Additionally, douching significantly increased the odds for testing positive for *Candida albicans.* Three douching intervention studies failed to show a significant effect of douching cessation on the vaginal microbiota(9, 29), although one study did observe significantly reduced candidiasis prevalence.(30) The specific effect of herb use on the vaginal microbiota is currently unknown and may differ by study population and by specific herb used. Compared to Caucasian Women, women of African descent are more likely to have vaginal microbiota not dominated by *Lactobacillus spp.*.(11, 31, 32) More research is needed to evaluate the impact of vaginal herb use on the vaginal microbiome and its potential to cause dysbiosis. Since vaginal herb use involves female hygiene, sexuality, and cultural identity, it is considered a sensitive subject in Surinamese society.(33) Therefore, the anthropologic and psychological aspects of vaginal herb use should be studied in detail to shed more light on this widely used phenomenon.

In conclusion, in the multi-ethnic society of Suriname, many Maroon and Creole women use vaginal herbs. Apart from ethnic group, education and being recruited at an STI clinic (as opposed to a FP clinic) were the main determinants for vaginal herb use. Vaginal herb use was not associated with *C. trachomatis* infection . Whether vaginal herb use has beneficial or possible negative effects on female health needs to be assessed in future studies.

For peer review only - http://bmjopen.bmj.com/site/about/guidelines.xhtml

Acknowledgements

The author would like to thank dr. L.O.A. Sabajo, all nurses and laboratory technicians of the Dermatological Service and the Lobi Foundation for data collection, and Titia Heijman for helpful suggestions

For peer review only - http://bmjopen.bmj.com/site/about/guidelines.xhtml

Bibliography

1. Blythe MJ, Fortenberry JD, Orr DP. Douching behaviors reported by adolescent and young adult women at high risk for sexually transmitted infections. J Pediatr Adolesc Gynecol. 2003;16(2):95-100.
2. Misra DP, Trabert B, Atherly-Trim S. Variation and predictors of vaginal douching behavior. Womens Health Issues. 2006;16(5):275-82.
3. van Andel T, de Korte S, Koopmans D, Behari-Ramdas J, Ruysschaert S. Dry sex in Suriname. J Ethnopharmacol. 2008;116(1):84-8.
4. Low N, Chersich MF, Schmidlin K, Egger M, Francis SC, van de Wijgert JH, et al. Intravaginal practices, bacterial vaginosis, and HIV infection in women: individual participant data meta-analysis. PLoS Med. 2011;8(2):e1000416.
5. Oh MK, Merchant JS, Brown P. Douching behavior in high-risk adolescents. What do they use, when and why do they douche? J Pediatr Adolesc Gynecol. 2002;15(2):83-8.
6. Markham CM, Tortolero SR, Addy RC, Baumler ER, McKirahan NK, Escobar-Chaves SL, et al. Factors associated with frequent vaginal douching among alternative school youth. J Adolesc Health. 2007;41(5):509-12.
7. Bui TC, Thai TN, Tran LT, Shete SS, Ramondetta LM, Basen-Engquist KM. Association Between Vaginal Douching and Genital Human Papillomavirus Infection Among Women in the United States. J Infect Dis. 2016;214(9):1370-5.
8. Tsai CS, Shepherd BE, Vermund SH. Does douching increase risk for sexually transmitted infections? A prospective study in high-risk adolescents. Am J Obstet Gynecol. 2009;200(1):38 e1-8.
9. Brotman RM, Klebanoff MA, Nansel TR, Andrews WW, Schwebke JR, Zhang J, et al. A longitudinal study of vaginal douching and bacterial vaginosis--a marginal structural modeling analysis. Am J Epidemiol. 2008;168(2):188-96.
10. Borgdorff H, Tsivtsivadze E, Verhelst R, Marzorati M, Jurriaans S, Ndayisaba GF, et al. Lactobacillus-dominated cervicovaginal microbiota associated with reduced HIV/STI prevalence and genital HIV viral load in African women. ISME J. 2014;8(9):1781-93.
11. Borgdorff H, van der Veer C, van Houdt R, Alberts CJ, de Vries HJ, Bruisten SM, et al. The association between ethnicity and vaginal microbiota composition in Amsterdam, the Netherlands. PLoS One. 2017;12(7):e0181135.
12. Gosmann C, Anahtar MN, Handley SA, Farcasanu M, Abu-Ali G, Bowman BA, et al. Lactobacillus-Deficient Cervicovaginal Bacterial Communities Are Associated with Increased HIV Acquisition in Young South African Women. Immunity. 2017;46(1):29-37.
13. van der Veer C, Bruisten SM, van der Helm JJ, de Vries HJ, van Houdt R. The Cervicovaginal Microbiota in Women Notified for Chlamydia trachomatis Infection: A Case-Control Study at the Sexually Transmitted Infection Outpatient Clinic in Amsterdam, The Netherlands. Clin Infect Dis. 2017;64(1):24-31.
14. Turner AN, Morrison CS, Munjoma MW, Moyo P, Chipato T, van de Wijgert JH. Vaginal practices of HIV-negative Zimbabwean women. Infect Dis Obstet Gynecol. 2010;2010.
15. Ness RB, Hillier SL, Kip KE, Richter HE, Soper DE, Stamm CA, et al. Douching, pelvic inflammatory disease, and incident gonococcal and chlamydial genital infection in a cohort of high-risk women. Am J Epidemiol. 2005;161(2):186-95.
16. Ott MA, Ofner S, Fortenberry JD. Beyond douching: use of feminine hygiene products and STI risk among young women. J Sex Med. 2009;6(5):1335-40.
17. van der Helm JJ, Bom RJ, Grunberg AW, Bruisten SM, Schim van der Loeff MF, Sabajo LO, et al. Urogenital Chlamydia trachomatis infections among ethnic groups in Paramaribo, Suriname; determinants and ethnic sexual mixing patterns. PLoS One. 2013;8(7):e68698.
18. van 't Klooster C, Haabo V, Ruysschaert S, Vossen T, van Andel TR. Herbal bathing: an analysis of variation in plant use among Saramaccan and Aucan Maroons in Suriname. J Ethnobiol Ethnomed. 2018;14(1):20.
19. van der Helm JJ, Sabajo LO, Grunberg AW, Morre SA, Speksnijder AG, de Vries HJ. Point-of-care test for detection of urogenital chlamydia in women shows low sensitivity. A performance evaluation study in two clinics in Suriname. PLoS One. 2012;7(2):e32122.
20. Bom RJ, van der Helm JJ, Bruisten SM, Grunberg AW, Sabajo LO, Schim van der Loeff MF, et al. The role of Surinamese migrants in the transmission of Chlamydia trachomatis between Paramaribo, Suriname and Amsterdam, The Netherlands. PLoS One. 2013;8(11):e77977.

For peer review only - http://bmjopen.bmj.com/site/about/guidelines.xhtml

21. van Melle A, Parriault MC, Basurko C, Jolivet A, Flamand C, Pigeon P, et al. Knowledge, attitudes, behaviors, and practices differences regarding HIV in populations living along the Maroni river: particularities of operational interest for Amerindian and Maroon populations. AIDS Care. 2015;27(9):1112-7.
22. Heng LS, Yatsuya H, Morita S, Sakamoto J. Vaginal douching in Cambodian women: its prevalence and association with vaginal candidiasis. J Epidemiol. 2010;20(1):70-6.
23. Li J, Jiang N, Yue X, Gong X. Vaginal douching and sexually transmitted infections among female sex workers: a cross-sectional study in three provinces in China. Int J STD AIDS. 2015;26(6):420-7.
24. Carter M, Gallo M, Anderson C, Snead MC, Wiener J, Bailey A, et al. Intravaginal cleansing among women attending a sexually transmitted infection clinic in Kingston, Jamaica. West Indian Med J. 2013;62(1):56-61.
25. Hickey RJ, Zhou X, Pierson JD, Ravel J, Forney LJ. Understanding vaginal microbiome complexity from an ecological perspective. Transl Res. 2012;160(4):267-82.
26. Hutchinson KB, Kip KE, Ness RB, Gynecologic Infection Follow-Through I. Vaginal douching and development of bacterial vaginosis among women with normal and abnormal vaginal microflora. Sex Transm Dis. 2007;34(9):671-5.
27. McClelland RS, Richardson BA, Graham SM, Masese LN, Gitau R, Lavreys L, et al. A prospective study of risk factors for bacterial vaginosis in HIV-1-seronegative African women. Sex Transm Dis. 2008;35(6):617-23.
28. Veer CV, Bruisten S, Houdt RV, editors. O04.2 Effects of over-the-counter lactic acid-containing vaginal douching products on the vaginal microbiota. Sex Transm Infect; 2017.
29. Klebanoff MA, Andrews WW, Yu KF, Brotman RM, Nansel TR, Zhang J, et al. A pilot study of vaginal flora changes with randomization to cessation of douching. Sex Transm Dis. 2006;33(10):610-3.
30. Sivapalasingam S, McClelland RS, Ravel J, Ahmed A, Cleland CM, Gajer P, et al. An effective intervention to reduce intravaginal practices among HIV-1 uninfected Kenyan women. AIDS Res Hum Retroviruses. 2014;30(11):1046-54.
31. Ravel J, Gajer P, Abdo Z, Schneider GM, Koenig SS, McCulle SL, et al. Vaginal microbiome of reproductive-age women. Proc Natl Acad Sci U S A. 2011;108 Suppl 1:4680-7.
32. Fettweis JM, Brooks JP, Serrano MG, Sheth NU, Girerd PH, Edwards DJ, et al. Differences in vaginal microbiome in African American women versus women of European ancestry. Microbiology. 2014;160(Pt 10):2272-82.
33. Terborg JRH. Sexual behaviour and sexually transmitted diseases among Saramaka and Ndjuka Maroons in the hinterland of Suriname. Paramaribo: MZ Primary Health Care Suriname : ProHealth; 2001. v, 83 p. p.

For peer review only - http://bmjopen.bmj.com/site/about/guidelines.xhtml

Table 1. Epidemiological characteristics of Creole, Hindustani, Javanese, Maroon and Mixed descent women (n=1040) recruited at the Family planning clinic and STI clinic, Paramaribo, Suriname, 2009-2010.

		Creole	Hindustani	Javanese	Maroon	Mixed descent	P value
		(N=278)	(N=256)	(N=163)	(N=138)	(N=205)	
		n (%)	n (%)	n (%)	n (%)	n (%)	
**Demographic characteristics**							
Recruitment site							<0.001
	Family planning clinic	199 (71.6)	226 (88.3)	146 (89.6)	85 (61.6)	138 (67.3)	
	STI clinic	79 (28.4)	30 (11.7)	17 (10.4)	53 (38.4)	67 (32.7)	
Median age in years [IQR]		29 [24-35]	31 [26-39]	30 [25-36]	28 [23-34]	27 [23-33]	<0.001
Age in years	<25	81 (29.1)	44 (17.2)	39 (23.9)	48 (34.8)	69 (33.7)	0.001
	25-29	64 (23.0)	63 (24.6)	41 (25.2)	29 (21.0)	53 (25.9)	
	30-34	60 (21.6)	50 (19.5)	33 (20.2)	30 (21.7)	40 (19.5)	
	≥35	73 (26.3)	99 (38.7)	50 (30.7)	31 (22.5)	43 (21.0)	

For peer review only - http://bmjopen.bmj.com/site/about/guidelines.xhtml

Education							<0.001
	Low	83 (29.9)	103 (40.2)	51 (31.3)	78 (56.5)	40 (19.5)	
	Medium	147 (52.9)	130 (50.8)	89 (54.6)	41 (29.7)	109 (53.2)	
	High	43 (15.5)	21 (8.2)	21 (12.9)	9 (6.5)	52 (25.4)	
	Unknown	5 (1.8)	2 (0.8)	2 (1.2)	10 (7.2)	4 (2.0)	
**Symptoms**							
Any symptoms		204 (73.4)	181 (70.7)	120 (73.6)	117 (84.8)	140 (68.3)	0.012
Dysuria[a]		60 (21.8)	80 (31.2)	39 (23.9)	46 (33.6)	42 (20.6)	0.008
Dyspareunia** [b]		66 (24.3)	73 (29.0)	41 (25.2)	41 (31.1)	51 (24.9)	0.506
Change in fluor/vaginal discharge[c]		149 (54.0)	126 (49.2)	92 (56.4)	79 (57.7)	110 (53.7)	0.488
Irregular menstruation [d]		81 (29.3)	71 (28.1)	32 (19.9)	51 (38.1)	52 (25.7)	0.012
Abdominal pain[e]		106 (39.8)	106 (41.9)	67 (42.1)	74 (54.4)	78 (38.6)	0.041
**Vaginal hygiene**							

For peer review only - http://bmjopen.bmj.com/site/about/guidelines.xhtml

Performs vaginal hygiene		109 (39.2)	51 (19.9)	29 (17.8)	112 (81.2)	78 (38.0)	<0.001
Vaginal products used[f]	Water	35 (12.6)	28 (10.9)	17 (10.4)	29 (21.0)	31 (15.1)	0.039
	Herbs	70 (25.2)	9 (3.5)	8 (4.9)	95 (68.8)	29 (14.1)	<0.001
	Lactacyd®	9 (3.2)	5 (2.0)	1 (0.6)	2 (1.4)	14 (6.8)	0.003
	Vinegar	9 (3.2)	2 (0.8)	4 (2.5)	2 (1.4)	9 (4.4)	0.118
	Dettol®	4 (1.4)	4 (1.6)	0 (0.0)	0 (0.0)	0 (0.0)	0.105
	Other[g]	10 (3.6)	7 (2.7)	2 (1.2)	1 (0.7)	4 (2.0)	0.314
Frequency of vaginal hygiene[h] #							0.027
	Daily	36 (43.9)	11 (32.4)	7 (41.2)	53 (63.1)	25 (39.7)	
	Once a week	18 (22.0)	12 (35.3)	3 (17.6)	19 (22.6)	11 (17.5)	
	Once a month	19 (23.2)	7 (20.6)	5 (29.4)	7 (8.3)	20 (31.7)	
	Less than once a month	9 (11.0)	4 (11.8)	2 (11.8)	5 (6.0)	7 (11.1)	
Reason for vaginal use of herbs[f,i] ##	Hygiene	50 (71.4)	4 (44.4)	5 (62.5)	49 (51.6)	16 (55.2)	0.107
	Sexual	24 (34.3)	0 (0.0)	1 (12.5)	46 (48.4)	11 (37.9)	0.0150.485
	Health	11 (15.7)	0 (0.0)	1 (12.5)	16 (16.8)	2 (6.9)	-*
	Other	3 (4.3)	0 (0.0)	0 (0.0)	6 (6.3)	0 (0.0)	

For peer review only - http://bmjopen.bmj.com/site/about/guidelines.xhtml

Last vaginal use of herbs[j] ##							-*
	Today	7 (12.7)	0 (0.0)	0 (0.0)	19 (26.4)	1 (5.9)	
	Yesterday	5 (9.1)	0 (0.0)	0 (0.0)	23 (31.9)	1 (5.9)	
	Last week	20 (36.4)	0 (0.0)	2 (33.3)	15 (20.8)	4 (23.5)	
	More than a week ago	23 (41.8)	4 (100.0)	4 (66.7)	15 (20.8)	11 (64.7)	
**Sexual behaviour**							
Number of partners in the preceding month[k]							<0.001
	0	17 (6.2)	8 (3.1)	2 (1.2)	10 (7.5)	11 (5.7)	
	1	237 (87.1)	236 (92.5)	148 (91.4)	117 (87.3)	158 (82.3)	
	2	16 (5.9)	6 (2.4)	11 (6.8)	7 (5.2)	10 (5.2)	
	>2	2 (0.7)	5 (2.0)	1 (0.6)	0 (0.0)	13 (6.8)	
Condom use during sex[l]							<0.001
	Always	49 (18.0)	17 (6.7)	10 (6.2)	23 (16.8)	37 (18.0)	
	Never or inconsistent	223 (82.0)	236 (93.3)	152 (93.8)	114 (83.2)	168 (82.0)	

For peer review only - http://bmjopen.bmj.com/site/about/guidelines.xhtml

Number of partners in the preceding 12 months							<0.001
	0	8 (2.9)	12 (4.7)	5 (3.1)	11 (8.0)	11 (5.4)	
	1	208 (74.8)	209 (81.6)	123 (75.5)	82 (59.4)	127 (62.0)	
	2	35 (12.6)	27 (10.5)	20 (12.3)	33 (23.9)	35 (17.1)	
	>2	27 (9.7)	8 (3.1)	15 (9.2)	12 (8.7)	32 (15.6)	
Ethnic sexual mixing	Reported ≥ 1 sexual partner from another ethnic group	89 (32.0)	45 (17.6)	80 (49.1)	43 (31.2)	123 (60.0)	<0.001
Sex in exchange for money or goods[b]		7 (2.6)	6 (2.4)	0 (0.0)	5 (3.7)	19 (9.4)	<0.001
Chlamydia trachomatis infection ###		37 (13.3)	16 (6.2)	25 (15.3)	15 (10.9)	29 (14.1)	0.022

a: 5 missings, b: 16 missings, c: 3 missings, d: 14 missings, e:24 missings, f: multiple options could be chosen, g: other vaginal practices are products like lactacyd but from other brands; soap, anti fungal and eggs., h: 99 missings, i: 56 missings, % of those who filled in the question, j: 57 missings, k: 25 missings, l: 11 missings.
*: *p* values could not obtained due to low numbers. IQR = interquartile range.

** pain during sexual intercourse

the denominator for the percentages is the group of women who indicated they performed vaginal hygiene.

the denominator for the percentages is the group of women who indicated they performed vaginal hygiene.

as diagnosed by nucleic acid amplification test

For peer review only - http://bmjopen.bmj.com/site/about/guidelines.xhtml

Table 2. Univariable and multivariable logistic regression analyses of determinants associated with vaginal herb use among Creole and Maroon women, Paramaribo, Suriname, 2009-2010.

		Use of vaginal herbs	Univariable OR (95%CI)	*P* value	Multivariable Adjusted OR (95%CI)*	*P* value
		n/N (%)				
**Demographic characteristics**						
Ethnic groups	Creole	70/278 (25.2)	1	<0.001	1	<0.001
	Maroon	95/138 (68.8)	6.57 (4.18-10.30)		5.33 (3.26-8.71)	
Recruitment site	Family planning clinic	96/284 (33.8)	1	<0.001	1	0.005
	STI clinic	69/132 (52.3)	2.15 (1.41-3.27)		2.04 (1.24-3.36)	
Age in years				0.068		0.067
	<25	59/129 (45.7)	1.99 (1.15-3.42)		2.23 (1.17-4.27)	
	25-29	35/93 (37.6)	1.42 (0.79-2.57)		1.78 (0.88-3.57)	
	30-34	40/90 (44.4)	1.88 (1.04-3.40)		2.23 (1.13-4.42)	
	≥35	31/104 (29.8)	1		1	
Education[a]				<0.001		0.002
	Low	88/161 (54.7)	5.06 (2.38-10.79)		3.80 (1.68-8.57)	
	Medium	59/188 (31.4)	1.92 (0.90-4.09)		2.02 (0.90-4.51)	
	High	10/52 (19.2)	1		1	

For peer review only - http://bmjopen.bmj.com/site/about/guidelines.xhtml

**Sexual behaviour**				
Number partners preceding month[b]				0.106
	0	10/27 (37.0)	1	
	1	135/354 (38.1)	1.05 (0.47-2.36)	
	≥2	15/25 (60.0)	2.55 (0.83-7.80)	
Number partners preceding 12 months				0.216
	0	9/19 (47.4)	1	
	1	107/290 (36.9)	0.65 (0.26-1.65)	
	≥2	49/107 (45.8)	0.94 (0.35-2.50)	
Ethnic sexual mixing	No	115/284 (40.5)	1	0.612
	Yes	50/132 (37.9)	0.90 (0.59-1.37)	
Sex in exchange for money or goods[c]	No	155/396 (39.1)	1	0.191
	Yes	7/12 (58.3)	2.17 (0.68-6.98)	
**Symptoms**				
Any symptoms	No	24/95 (25.3)	1	0.001
	Yes	141/321 (43.9)	2.32 (1.39-3.87)	
Dysuria[d]	No	114/306 (37.3)	1	0.104
	Yes	49/106 (46.2)	1.45 (0.93-2.26)	

For peer review only - http://bmjopen.bmj.com/site/about/guidelines.xhtml

Dyspareunia (pain during sexual intercourse)[e]	No<br>Yes	113/297 (38.0)<br>47/107 (43.9)	1<br>1.28 (0.82-2.00)	0.287
Change in fluor/vaginal discharge[f]	No<br>Yes	71/185 (38.4)<br>93/228 (40.8)	1<br>1.11 (0.74-1.65)	0.619
Irregular menstruation[g]	No<br>Yes	102/278 (36.7)<br>59/132 (44.7)	1<br>1.40 (0.92-2.13)	0.122
Abdominal pain[h]	No<br>Yes	78/222 (35.1)<br>83/180 (46.1)	1<br>1.58 (1.06-2.36)	0.026

*ORs in the multivariable model are adjusted for all factors for which adjusted ORs are shown. In the final model 401 participants were included.
a:15 missings, b: 10 missings, c: 8 missings, d: 4 missings , e: 12 missings, f: 3 missings, g: 6 missings, h: 14 missings

For peer review only - http://bmjopen.bmj.com/site/about/guidelines.xhtml

Table 3. Vaginal products mentioned among female participants performing vaginal hygiene, Paramaribo, Suriname, 2009-2010.

Vaginal product (scientific names if applicable)	Maroon ethnicity (N=112)	Creole ethnicity (N=109)	Other ethnicities[a] (N=158)
Almond leaves (*Terminalia catappa*)	1	3	1
Andoya (*Campomanesia aromatica*)	6	1	1
Blaka masusa	0	1	0
Blaka uma	0	5	2
Broko pie (*Bellucia grossularioides*)	1	1	0
Dettol®	0	4	4
Djamu (*Syzygium cumini*)	9	4	0
Douchegel	0	0	1
Dram (alcohol)	0	1	0
Eggs	0	1	0
Eva products	0	1	0
Faya lobi (*Ixora coccinea)*	2	0	0
Feififingawiwiri	1	1	0
Guave (*Psidium guajava)*	3	2	0
Intimate wash products	0	3	5
Kill somebody (*Dimorphandra conjugata)*	13	4	1
Lactacyd®	2	9	20
Manjablad (Mango leaves)	4	9	3
Odany jewa	0	0	3
Paraklem	4	1	0
Pedreku	1	1	2
Pikin bë (*Vismia* Vand. spp)	7	5	14
Redikatun (*Gossypium barbadense*)	4	16	6
Suku trobi	7	3	0
Twigs	0	0	1
Umaanesi	4	1	2
Unknown leaves	13	6	6
Vagisil	0	1	0
Vinegar	2	9	15

For peer review only - http://bmjopen.bmj.com/site/about/guidelines.xhtml

Wasduku (*Clidemia capitellata*)	9	1	3
Water	29	35	76
Yarakopie	2	3	1
Yoghurt	0	0	1

a: Hindustani, Javanese and Mixed race ethnicity

For peer review only - http://bmjopen.bmj.com/site/about/guidelines.xhtml

Table 4. Univariable and multivariable logistic regression analyses of variables associated with cervicovaginal *C. trachomatis* infection among Creole and Maroon women, Paramaribo, Suriname, 2009-2010.

		NAAT positive	Univariable OR (95%CI)	*P* value	Multivariable Adjusted OR (95%CI)*	*P* value
		n/N (%)				
**Demographic characteristics**						
Ethnic group	Creole	37/278 (13.3)	1	0.479		
	Maroon	15/138 (10.9)	0.79 (0.42-1.50)			
Recruitment site	Family planning clinic	25/284 (8.8)	1	0.001	1	0.003
	STI clinic	27/132 (20.5)	2.66 (1.48-4.80)		2.59 (1.39-4.83)	
Age in years				0.001		0.002
	<25	25/129 (19.4)	4.76 (1.75-12.92)		4.37 (1.59-12.00)	
	25-29	17/93 (18.3)	4.43 (1.56-12.54)		4.96 (1.72-14.26)	
	30-34	5/90 (5.6)	1.17 (0.33-4.16)		1.20 (0.33-4.35)	
	≥35	5/104 (4.8)	1		1	

Education				0.444		
	Low	19/161 (11.8)	1.03 (0.39-2.73)			
	Medium	23/188 (12.2)	1.07 (0.41-2.78)			
	High	6/52 (11.5)	1			
	Unknown	4/15 (26.7)	2.79 (0.67-11.60)			
**Vaginal hygiene**						
Performed vaginal hygiene	No	21/195 (10.8)	1	0.317		
	Yes	31/221 (14.0)	1.35 (0.75-2.44)			
Herb use	No	27/251 (10.8)	1	0.187	1	0.564
	Yes	25/165 (15.2)	1.48 (0.83-2.66)		1.20 (0.65-2.22)	
Frequency of performing vaginal hygiene[b]	Daily	16/89 (18.0)	1.82 (0.90-3.68)	0.282		
	At least once a week	7/36 (19.4)	2.00 (0.78-513)			
	At least once a month	2/25 (8.0)	0.72 (0.16-3.28)			
	Less then once a month	3/14 (21.4)	2.26 (0.58-8.76)			
	Never	21/195 (10.8)	1			
**Sexual behaviour**						
Number of partners preceding month[c]	0-1	43/381 (11.3)	1	0.018		
	≥2	7/25 (28.0)	3.06 (1.21-7.74)			

For peer review only - http://bmjopen.bmj.com/site/about/guidelines.xhtml

Condom use[d]	Always	11/72 (15.3)	1.30 (0.63-2.68)	0.473
	Never or inconsistent	41/337 (12.2)	1	
Number of partners in the preceding 12 months	0-1	37/309 12.0)	1	0.582
	≥2	15/107 (14.0)	1.20 (0.63-2.28)	
Ethnic sexual mixing	Reported only sexual partners from own ethnic group	32/284 (11.3)	1	0.267
	Reported at least one sexual partner from another ethnic group	20/132 (15.2)	1.41 (0.77-2.57)	
Sex in exchange for money or goods[e]	No	47/396 (11.9)	1	0.038
	Yes	4/12 (33.3)	3.71 (1.08-12.81)	

*ORs in the multivariable model are adjusted for all factors for which adjusted ORs are shown. In the final model 416 participants were included.
Footnote: NA: not applicable.
, b: 57 missings, c: 10 missings, d: 7 missings, e: 8 missings,

For peer review only - http://bmjopen.bmj.com/site/about/guidelines.xhtml

STROBE Statement—Checklist of items that should be included in reports of ***cross-sectional studies***

	Item No	Recommendation	Reported on page #
**Title and abstract**	1	(*a*) Indicate the study's design with a commonly used term in the title or the abstract	1
		(*b*) Provide in the abstract an informative and balanced summary of what was done and what was found	2
**Introduction**			
Background/rationale	2	Explain the scientific background and rationale for the investigation being reported	4
Objectives	3	State specific objectives, including any prespecified hypotheses	4/5
**Methods**			
Study design	4	Present key elements of study design early in the paper	5
Setting	5	Describe the setting, locations, and relevant dates, including periods of recruitment, exposure, follow-up, and data collection	5
Participants	6	(*a*) Give the eligibility criteria, and the sources and methods of selection of participants	5
Variables	7	Clearly define all outcomes, exposures, predictors, potential confounders, and effect modifiers. Give diagnostic criteria, if applicable	5/6
Data sources/ measurement	8*	For each variable of interest, give sources of data and details of methods of assessment (measurement). Describe comparability of assessment methods if there is more than one group	*6*
Bias	9	Describe any efforts to address potential sources of bias	5/6
Study size	10	Explain how the study size was arrived at	Ref 19
Quantitative variables	11	Explain how quantitative variables were handled in the analyses. If applicable, describe which groupings were chosen and why	6
Statistical methods	12	(*a*) Describe all statistical methods, including those used to control for confounding	6
		(*b*) Describe any methods used to examine subgroups and interactions	6
		(*c*) Explain how missing data were addressed	15
		(*d*) If applicable, describe analytical methods taking account of sampling strategy	n.a.
		(*e*) Describe any sensitivity analyses	6
**Results**			

For peer review only - http://bmjopen.bmj.com/site/about/guidelines.xhtml

Participants	13*	(a) Report numbers of individuals at each stage of study—eg numbers potentially eligible, examined for eligibility, confirmed eligible, included in the study, completing follow-up, and analysed	6
		(b) Give reasons for non-participation at each stage	n.a.
		(c) Consider use of a flow diagram	n.a.
Descriptive data	14*	(a) Give characteristics of study participants (eg demographic, clinical, social) and information on exposures and potential confounders	6/13
		(b) Indicate number of participants with missing data for each variable of interest	15
Outcome data	15*	Report numbers of outcome events or summary measures	7/16/20
Main results	16	(*a*) Give unadjusted estimates and, if applicable, confounder-adjusted estimates and their precision (eg, 95% confidence interval). Make clear which confounders were adjusted for and why they were included	16/20
		(*b*) Report category boundaries when continuous variables were categorized	13-21
		(*c*) If relevant, consider translating estimates of relative risk into absolute risk for a meaningful time period	n.a.
Other analyses	17	Report other analyses done—eg analyses of subgroups and interactions, and sensitivity analyses	n.a.
**Discussion**			
Key results	18	Summarise key results with reference to study objectives	8
Limitations	19	Discuss limitations of the study, taking into account sources of potential bias or imprecision. Discuss both direction and magnitude of any potential bias	8
Interpretation	20	Give a cautious overall interpretation of results considering objectives, limitations, multiplicity of analyses, results from similar studies, and other relevant evidence	8/9
Generalisability	21	Discuss the generalisability (external validity) of the study results	9
**Other information**			
Funding	22	Give the source of funding and the role of the funders for the present study and, if applicable, for the original study on which the present article is based	1

*Give information separately for exposed and unexposed groups.

Note: An Explanation and Elaboration article discusses each checklist item and gives methodological background and published examples of transparent reporting. The STROBE checklist is best used in conjunction with this article (freely available on the Web sites of PLoS Medicine at http://www.plosmedicine.org/, Annals of Internal Medicine at

For peer review only - http://bmjopen.bmj.com/site/about/guidelines.xhtml
For peer review only

http://www.annals.org/, and Epidemiology at http://www.epidem.com/). Information on the STROBE Initiative is available at www.strobe-statement.org.

For peer review only

For peer review only - http://bmjopen.bmj.com/site/about/guidelines.xhtml

Vaginal herb use and Chlamydia trachomatis infection: a cross-sectional study among women of various ethnic groups in Suriname

Journal:	*BMJ Open*
Manuscript ID	bmjopen-2018-025417.R2
Article Type:	Research
Date Submitted by the Author:	26-Feb-2019
Complete List of Authors:	Van der Helm, Jannie; Public Health Service Amsterdam, Department of Research<br>Schim van der Loeff, Maarten; GGD Amsterdam<br>de Vries, Esther; Public Health Service Amsterdam, Department of Research<br>van der Veer, Charlotte; GGD Amsterdam, Streeklaboratorium<br>Grünberg, Antoon; Ministry of Health, Paramaribo, Suriname, Department of Public Health<br>Mans, Dennis; Department of Pharmacology<br>de Vries, H; University of Amsterdam, Department of Dermatology
**Primary Subject Heading**:	Sexual health
Secondary Subject Heading:	Global health, Infectious diseases, Obstetrics and gynaecology, Public health, Epidemiology
Keywords:	Suriname, ethnicity, vaginal, vaginal douching, herb therapy

Vaginal herb use and *Chlamydia trachomatis* infection: a cross-sectional study among women of various ethnic groups in Suriname

Running title: Vaginal herbal use and chlamydia in Suriname

Jannie J. van der Helm PhD[1], Maarten F. Schim van der Loeff MD, PhD[2,5,], Esther de Vries, MSc[2], Charlotte van der Veer[2], Antoon W. Grünberg MD, PhD[3], and Dennis R. Mans PhD[4], Henry J.C. de Vries MD, PhD[2,5]

1. Centre for Environmental Safety and Security, National Institute of Public Health and the Environment, Bilthoven
2. Public Health Service (GGD) Amsterdam, Department of Infectious diseases, Amsterdam, the Netherlands
3. Department of Public Health, Ministry of Health, Paramaribo, Suriname
4. Department of Pharmacology, Faculty of Medical Sciences, Anton de Kom University of Suriname, Paramaribo, Suriname
5. Amsterdam UMC, University of Amsterdam, department of dermatology, Amsterdam Institute for Infection and Immunity (AI&II), Meibergdreef 9, Amsterdam, Netherlands

Corresponding author:
Henry John Christiaan de Vries
Department of Dermatology, Academic Medical Center, University of Amsterdam
Meibergdreef 9, 1105 AZ Amsterdam-Zuidoost, the Netherlands,
Phone: +31 (0)20 566 9111, Fax: +31 (0)20 566 4440 email: h.j.devries@amc.nl

Word count of the abstract: 242
Word count of the text: 2872
Number of references: 33
Number of tables: 4
Number of figures: 0

Conflict of Interest: None reported

Funding: This work was supported by the Research and Development fund of the Public Health Service of Amsterdam [project no 2369 and 2371] and AGIS healthcare insurance [RVVZ no 1417000]. The funders had no role in study design, data collection and analysis, decision to publish, or preparation of the manuscript.

Contributorship statement: JvdH collected the data, analysed the data and wrote the manuscript; MSvdL supervised the analysis and wrote the manuscript; EdV analysed the data and wrote the first manuscript draft; CvdV wrote the manuscript; AWG designed the study, collected data and

For peer review only - http://bmjopen.bmj.com/site/about/guidelines.xhtml

wrote the manuscript; DRM collected the data and wrote the manuscript; and HdV designed the study, and wrote the manuscript.

Abstract

Objective: Vaginal steam baths with herb leaves (herb use) is practiced by some Surinamese women. We assessed herb use among women from the five most prevalent ethnic groups, and if herb use is associated with *Chlamydia trachomatis* infection.

Setting: Participants were recruited at a sexually transmitted infection clinic (STI) and a family planning clinic (FP) in Paramaribo, Suriname.

Participants: 1040 women were included subsequently, comprising the following ethnic groups: Creole (26.7%), Hindustani (24.6%), Javanese (15.7%), Maroon (13.3%) and Mixed descent (19.7%).

Methods: Nurses collected a questionnaire and vaginal swabs for nucleic acid amplification *C. trachomatis* testing.

Primary outcomes: Determinants of vaginal herb use and *C. trachomatis* infection via univariable and multivariable logistic regression.

Results: Herb use was most common among Maroon (68.8%) and Creole women (25.2%). In multivariable analysis including only Maroon and Creole women, determinants significantly associated with vaginal herb use were (odds ratio; 95%CI): Maroon ethnic descent (5.33; 3.26-8.71 versus Creole), recruitment at the STI clinic (2.04;1.24-3.36 versus FP), lower education levels (3.80;1.68-8.57 lower versus higher, and 2.02;0.90-4.51 middle versus higher). Lower age and recruitment at the STI clinic *were associated with C. trachomatis* infection, but not vaginal herb use.

Conclusion: In Suriname, vaginal herb use is common among Maroon and Creole women. Education, ethnic group and recruitment site were determinants for herb use. Vaginal herb use was not a determinant of *C. trachomatis* infection. Future research should focus on the effect of herb use on the vaginal microbiome and mucosal barrier.

For peer review only - http://bmjopen.bmj.com/site/about/guidelines.xhtml

Strengths and limitations of this study:

- This is the first study into the relationship between vaginal herb use and a sexually transmitted infection in Suriname.
- Moreover, determinants such as education, ethnic group are related to vaginal herb use.
- Patients were recruited at 2 different settings STI outpatient clinic vs Family Planning clinic), allowing a broader analysis of various populations.
- We did not assess if vaginal herbs were used intra-vaginally and/or externally.
- We did not study the effect of vaginal herb use on the vaginal microbiota.

Keywords: herb therapy; vaginal douching; vaginal; ethnicity; Suriname

Data sharing statement: Deidentified participant data are available upon request through the corresponding author Henry J C de Vries. ORCID identifier 0000-0001-9784-547X

For peer review only - http://bmjopen.bmj.com/site/about/guidelines.xhtml

Introduction

Depending on cultural habits, women worldwide engage in a variety of vaginal hygiene practices, such as the use of intra-vaginal douches, herbal vaginal steam baths, or the direct insertion of herbs into the vagina.(1-4) Studies in the United States of America showed that women who had less income, less education, were unmarried, lived in the southern States, and were of African-American descent were more likely to engage in such practices when compared to white women.(2)

Vaginal practices are not only intended for feeling clean and fresh, getting rid of vaginal malodour, or removing residual menstrual blood(5, 6), but also for improving the appearance of the vagina and for enhancing sensation during intercourse, and securing the relationship with, and economic support by the male partner.(2) However, these practices may increase the risk of acquiring infections, such as HIV(4), HPV(7), but also bacterial sexually transmitted infections (STI) and trichmoniasis.(8) Particularly intra-vaginal practices, such as use of water and soap, the insertion of a cloth or a piece of paper into the vagina, to dry and tighten the vagina was associated with acquiring HIV.(4) An underlying mechanism for this may be that intra-vaginal practices deplete the relative amount of vaginal *Lactobacillus spp.*(4, 9) Lactobacilli produce anti-microbials and acidify the vagina by lactic-acid production and are thus considered to be a hallmark for vaginal health; their depletion is known to mediate HIV and STI acquisition.(4, 10-13)

Risk for STI may be further increased by a breakdown of the vaginal epithelial barrier and sometimes even the occurrence of lacerations.(3) In addition, a lack of vaginal fluids increases the risk of condom rupture. Although several studies have reported an association between vaginal practices and STI(1, 8, 14), other studies did not.(15, 16) Unfortunately earlier studies did not consider the type of vaginal practice nor the frequency of their use.

Suriname is located on the northeast coast of South America, bordering the Atlantic Ocean to the north and surrounded by French Guiana to the east, Brazil to the south, and Guyana to the west. The population of approximately 570,000 is characterized by the ethnical and cultural diversity. The largest ethnic groups are the Creoles and Maroons (both originating from enslaved Africans imported in the 17th to 19th century), and the Hindustani and Javanese (descendants from indentured laborers from the former British Indies and Dutch Indies, respectively, arrived around the turn of the 20th century). There are, in addition, Indigenous Amerindians, people of Mixed descent, and descendants from Chinese laborers, European colonists, and immigrants from various Latin American and Caribbean countries such as Brazil, Guyana, French Guiana, Haiti, and Cuba.(17)

Among these ethnic groups, Afro-Surinamese women (Creoles and Maroons) in particular engage in a variety of vaginal practices including vaginal steam baths containing certain herbs for drying and tightening the vagina, cleansing after menstruation or after birth to prevent puerperal fever, ‘placing the uterus back into position’, or preventing a flabby abdominal wall.(3) Typically, for a herbal genital steam bath, a woman sits with spread legs on a bucket or bidet containing warm water with certain herbs to steam her inner genital parts. Depending on the herbs, the cooled down bath is used to wash the genital parts after steaming.(18) For direct internal application, Dettol® (the brand name of the antiseptic chloroxylenol), yoghurt, lemon juice, vinegar as well as commercial products such as

For peer review only - http://bmjopen.bmj.com/site/about/guidelines.xhtml

effervescent tablets, gels, or emulsions are used. Commercial products in the form of wipes, emulsions, crèmes, and mousses can also be used externally.(3,18)

In this study we examined the type and frequency of vaginal practices in women visiting an STI outpatient clinic and a family planning (FP) clinic, in particular the use of herbs and the reasons for their use were identified. Moreover, we assessed factors associated with vaginal herb use, and examined whether vaginal herb use is a determinant of cervicovaginal *C. trachomatis* infection.

Methods

Ethics statement

The data used for the current study were gathered in the course of a larger study concerning chlamydia, the Urogenital Chlamydia Rapid Test Evaluation in Paramaribo and Amsterdam (CUSTEPA) study.(17, 19, 20) The study was approved by the Ethics Committees of the Ministry of Health of the Republic of Suriname (VG010-2007) and the Academic Medical Center, University of Amsterdam, The Netherlands (MEC07/127). Women participated after given written informed consent.

Study population

Recruitment took place in Suriname's capital city Paramaribo in the period 2009-2010 at two locations: the Dermatological Service, an integrated outpatient STI clinic that offers free-of-charge examination and treatment of STIs and infectious skin diseases such as leprosy and leishmaniasis; and the Lobi Foundation, a FP clinic. The study was cross-sectional, and each participant was given a unique code. Subsequently, a nurse interviewed the participant about demographic characteristics, including self-reported ethnic background, sexual behaviour, symptoms, STI history, and vaginal hygiene. Criteria for exclusion were age below 18 years and previous participation within the CUSTEPA study.

Patient and Public Involvement

Representatives of the Maroon population were consulted on the outcome of the study.

Vaginal hygiene practices

Using a structured questionnaire, nurses asked women in detail about the use of products for vaginal practices such as douches, herbs, or other home-made products, and if so, at which frequency. The questionnaires included several options such as the use of herbal genital steam baths, vinegar, water, yoghurt, and two often used commercial products: Lactacyd® (Omega Pharma Nederland BV, Rotterdam, The Netherlands) that consists of lactic acid-containing wipes, crèmes, emulsion, and mousses, and Dettol® (Reckitt Benckiser Group plc, Slough, UK). Participants could specify the products they used. Pre-coded answer options for the frequency of vaginal practices were: daily, weekly, monthly, or less than once a month. Women who used vaginal herbs were asked about their most recent application, and the reasons for use (hygiene, sexual pleasure, health or other).

For peer review only - http://bmjopen.bmj.com/site/about/guidelines.xhtml

Items mentioned under reason "other" that fitted one of the 3 former reasons were regrouped accordingly.

Specimen collection and testing procedures

Vaginal swabs were collected by trained nurses, stored and shipped to the Public Health Laboratory in Amsterdam, as described earlier.(19) Here, the samples were tested for *C. trachomatis* rRNA (APTIMA CT, Hologic Gen-Probe Inc, San Diego, USA) according to the manufacturer's instructions. Test results were sent to the clinics in Suriname, and participants (and partners if indicated) were managed as described earlier.(19)

Statistical analysis

To examine whether epidemiological characteristics and behaviour, including the use and frequency of vaginal practices, differed between women from the five major ethnic groups, characteristics and behaviours of these groups were compared using the χ^2-test; age was compared using the Kruskal-Wallis test. Univariable logistic regression analysis was used to assess determinants for the use of vaginal herbal. In univariable analysis the following variables were assessed: age, education, ethnic group, ethnicity of sexual partner, recruitment site, condom use, number of sexual partners in the preceding one and twelve month(s), having had sex in exchange for money or goods, and vaginal symptoms. Variables with P<0.05 in univariable analysis were entered into multivariable logistic regression models; variables were removed stepwise until only significant variables were retained, but based on earlier studies age was forced into the model.(1, 2). A second logistic regression analysis was conducted to assess whether vaginal herb use was independently associated with *C. trachomatis* infection. Variables univariately associated with *C. trachomatis* infection were entered into a multivariable model, and stepwise removed until only significant variables were retained; vaginal herb use was forced into the model. P values < 0.05 were considered statistically significant. SPSS statistics version 21 (IBM, City, State, USA) was used for the analysis.

Results

Study population

We initially included 1,093 women who self-identified with one of the following ethnic backgrounds: 17 (1.6%) Caucasian, 12 (1.1%) Chinese, 278 (25.4%) Creole, 256 (23.4%) Hindustani, 19 (1.7%) Indigenous, 163 (14.9%) Javanese, 138 (12.6%) Maroon, 205 (18.8%) Mixed descent, and 5 unknown (0.5%). Due to the small numbers we excluded Caucasian, Chinese, Indigenous women, and women with unknown background from further analysis. As a result, the study population included 1,040 women of either Creole, Hindustani, Javanese, Maroon, or Mixed descent (Table 1).

The majority of participants was recruited at the FP clinic (between 61.6% and 89.6% per ethnic background). The median age ranged from 27 years (interquartile range (IQR), 23-33 years) for Mixed descent women to 31 years (IQR, 26-39 years) for Hindustani women (P<0.001). Most women

For peer review only - http://bmjopen.bmj.com/site/about/guidelines.xhtml

from Maroon descent (56.5%) had lower education whereas most of those from the other ethnic backgrounds had at least medium education (P<0.001).

Vaginal symptoms were reported frequently, ranging from 68.3% (Mixed descent women) to 84.8% (Maroon women; P=0.01). In all ethnic groups, more than 80% of women reported one sexual partner during the preceding month, and more than 60% reported one sexual partner during the preceding 12 months. Discordant mixing (intercourse with a partner of a different ethnic group) was most frequently seen among Mixed descent and Javanese women (60.0% and 49.1%, respectively), followed by Creole, Maroon, and Hindustani women. The prevalence of *C. trachomatis* infections was highest in Javanese women (15.3%) followed by Mixed descent women (14.1%) and Creole women (13.3%) (P = 0.02).

Vaginal hygiene

Table 1 shows the ways in which vaginal hygiene was performed within the study population. Vaginal hygiene was most common in Maroon women (81.2%) followed by Creole (39.2%) and Mixed descent women (38.0%). Between 10.4% and 21.0% of women used tap water for vaginal cleansing, with the largest proportion in Maroon (21.0%) and Mixed descent women (15.1%). Lactacyd®, vinegar, and other substances such as Dettol® were used less often.

In those women that performed vaginal hygiene, daily use was reported by Maroons (63.1%), Creoles (43.9%), Javanese (41.2%), Mixed descent (39.7%), and Hindustani (32.4%).

Vaginal herb use

Vaginal herb use was reported most frequent by Maroon women (68.8%), followed by Creole women (25.2%), women of Mixed descent (14.1%), Javanese (4.9%) and Hindustani (3.5%) (Table 1). Hygiene was the most often mentioned reason for vaginal herb use (across all groups ranging from 68.1% to 100%), followed by sexual pleasure (ranging from 0% to 63.9%). Due to low numbers of women reporting vaginal herb use among women of Hindustani, Javanese and Mixed descent, only Maroon and Creole women were included in the further analyses of vaginal herb use.

In univariable analysis, vaginal herb use was significantly associated with recruitment location, education, ethnic background, vaginal symptoms, and abdominal pain (Table 2). Although not significant, younger women reported vaginal herb use more often than women aged 35 years and above. In multivariable analysis, vaginal herb use was significantly associated with recruitment at the STI clinic (OR 2.04; 95%CI 1.24-3.36 versus the FP clinic), lower education levels (OR 3.80; 95%CI 1.68-8.57 lower versus higher education; and OR 2.02; 95%CI 0.90-4.51 medium versus higher education), and Maroon ethnicity (OR, 5.33; 95%CI 3.26-8.71 versus Creole). Although not significant in multivariable analysis, younger age was associated with vaginal herb use.

As described in table 3, a wide variety of herbs were used. Maroon women predominantly used “kill somebody” or “kill your darling *Dimorphandra conjugata* (Splitg.) Sandw. (Fabaceae)” (13), the “jambolan or dyamu *Syzygium cumini* (L.) Skeels (Myrtaceae)” (9), the “towel or wasduku *Clidemia capitellata* (Bonpl.) D.Don (Melastomataceae)” (9), the “guavaberry or andoya *Campomanesia aromatica* (Aubl.) Griseb. (Myrtaceae)” (6), and “pikin bë/witi baka píyjá páu (small red/white backed

For peer review only - http://bmjopen.bmj.com/site/about/guidelines.xhtml

pineapple) *Vismia* Vand. spp. (Hypericaceae)" (7). Creole women mostly mentioned "sea island cotton or redikatun *Gossypium barbadense* L. (Malvaceae)" (16) in addition to the leaves of the "tropical-almond or amandra *Terminalia catappa* L. (Combretaceae)", the "mess apple or broko pi ('broken penis') *Bellucia grossularioides* (L.) Triana (Melastomataceae)", the "guava or guyaba *Psidium guajava* L. (Myrtaceae)", the "jungle geranium or faya lobi ('fiery love') *Ixora coccinea* L. (Rubiaceae)", and the "ant bush or kapasiwiwiri ('herb of the nine-banded armadillo') *Siparuna guianensis* Aubl. (Monimiaceae)".

V*aginal herb use and C. trachomatis infection*

V*a*ginal herb use was not associated with *C. trachomatis infection*, neither in univariable nor in multivariable analysis (aOR, 1.20; 95%CI: 0.65-2.22: p=0.564), (Table 4). In contrast, recruitment location, age, and reporting sex in exchange for money or goods, were associated with *C. trachomatis* infection in univariable analysis. In multivariable analysis, chlamydia infection was associated with recruitment at the STI clinic versus FP clinic (OR, 2.59; 95%CI: 1.39-4.83; p=0.003) and also with younger age, both for < 25 years, and 25-29 years versus ≥ 35 years (resp aOR, 4.37; 95%CI 1.59-12.00, aOR 4.96; CI95% 1.72-14.26 and aOR 1.20; CI95% 0.33-4.35; p=0.002).

Discussion

We assessed the use and frequency of various vaginal practices among female STI clinic and FP clinic visitors in Suriname, and found that: (1) vaginal practices are commonly used, and most frequently by Maroon, Creole and Mixed decent women; (2) vaginal herb use is more common among women with lower education; (3) vaginal herb use is most frequently practiced for hygienic reasons; and (4) vaginal herb use is not associated with *C. trachomatis* infection.

Ethnic background was the most important determinant for vaginal herb use. The extensive vaginal use of herbs among Maroon women has previously been described in French Guiana, where a prevalence of 96.1% was found.(21)

Women who experienced vaginal symptoms were more likely to engage in vaginal herb use. Since this is a cross-sectional study, it is not clear if vaginal symptoms are a cause, or an effect of vaginal practices. A Cambodian study also found an association between vaginal douching and vaginal symptoms.(22) Similarly, female sex workers in China were more likely to engage in vaginal practices when having STI-related symptoms, and reportedly engaged more in vaginal douching when experiencing vaginal symptoms.(23) In contrast, a study with Jamaican women who attended a public STI clinic found that vaginal itching led to a lower frequency of vaginal douching.(24) Prospective (intervention) studies could shed light on any casual links between vaginal practices and vaginal symptoms. The high prevalence of vaginal herb use among Maroon women has been previously reported, and it is conceivable that Maroon women do not engage in vaginal herb use as consequence of vaginal symptoms but rather out of (cultural) habit.(21)

For peer review only - http://bmjopen.bmj.com/site/about/guidelines.xhtml

As previously described, hygiene was the most important reason for vaginal herb use, followed by sexual reasons.(3) Moreover, Maroon women mentioned as a reason to make oneself more attractive for one's partner.

We found no association between vaginal herb use and infection with *C. trachomatis*. This confirms earlier findings of the above mentioned Chinese study where no association was found among sex workers between vaginal practices (mostly disinfectants after sex with clients) and STI (syphilis, *Neisseria gonorrhoea* and *C. trachomatis* combined).(23)

This study has some limitations. This study did not assess the mode of vaginal practices, e.g. whether the products were applied intra-vaginally and/or externally. Previous studies have found associations between the internal use of vaginal practices and HIV.(4) Therefore we cannot exclude that the lack of association between vaginal herb use and *C. trachomatis* infection may be explained by predominantly external use of herbs. Moreover we did not study the effect of vaginal herb use on the vaginal microbiota. Lactobacilli generally constitute a healthy vaginal microbiota as they generate an acidic environment (pH 4.0-4.5) and produce antimicrobials, that restrict the growth of most pathogens.(25) A non-lactobacillus-dominated vaginal microbiota is thus considered dysbiotic. Intra-vaginal practices have been linked to developing vaginal dysbiosis(4, 9, 26, 27), but these studies studied douching behaviour in general and did not differentiate between different types of intra-vaginal products. The use of an over-the-counter lactic-acid containing douche was recently studied prospectively among healthy Dutch women and this was not found to significantly impact the vaginal microbiota composition, although an increased odds for having non-lactobacillus dominated vaginal microbiota among users was observed.(28) Additionally, douching significantly increased the odds for testing positive for *Candida albicans.* Three douching intervention studies failed to show a significant effect of douching cessation on the vaginal microbiota(9, 29), although one study did observe significantly reduced candidiasis prevalence.(30) The specific effect of herb use on the vaginal microbiota is currently unknown and may differ by study population and by specific herb used. Compared to Caucasian Women, women of African descent are more likely to have vaginal microbiota not dominated by *Lactobacillus spp.*.(11, 31, 32) More research is needed to evaluate the impact of vaginal herb use on the vaginal microbiome and its potential to cause dysbiosis. Since vaginal herb use involves female hygiene, sexuality, and cultural identity, it is considered a sensitive subject in Surinamese society.(33) Therefore, the anthropologic and psychological aspects of vaginal herb use should be studied in detail to shed more light on this widely used phenomenon.

In conclusion, in the multi-ethnic society of Suriname, many Maroon and Creole women use vaginal herbs. Apart from ethnic group, education and being recruited at an STI clinic (as opposed to a FP clinic) were the main determinants for vaginal herb use. Vaginal herb use was not associated with *C. trachomatis* infection . Whether vaginal herb use has beneficial or possible negative effects on female health needs to be assessed in future studies.

For peer review only - http://bmjopen.bmj.com/site/about/guidelines.xhtml

Acknowledgements
The author would like to thank dr. L.O.A. Sabajo, all nurses and laboratory technicians of the Dermatological Service and the Lobi Foundation for data collection, and Titia Heijman for helpful suggestions

For peer review only - http://bmjopen.bmj.com/site/about/guidelines.xhtml

Bibliography

1. Blythe MJ, Fortenberry JD, Orr DP. Douching behaviors reported by adolescent and young adult women at high risk for sexually transmitted infections. J Pediatr Adolesc Gynecol. 2003;16(2):95-100.
2. Misra DP, Trabert B, Atherly-Trim S. Variation and predictors of vaginal douching behavior. Womens Health Issues. 2006;16(5):275-82.
3. van Andel T, de Korte S, Koopmans D, Behari-Ramdas J, Ruysschaert S. Dry sex in Suriname. J Ethnopharmacol. 2008;116(1):84-8.
4. Low N, Chersich MF, Schmidlin K, Egger M, Francis SC, van de Wijgert JH, et al. Intravaginal practices, bacterial vaginosis, and HIV infection in women: individual participant data meta-analysis. PLoS Med. 2011;8(2):e1000416.
5. Oh MK, Merchant JS, Brown P. Douching behavior in high-risk adolescents. What do they use, when and why do they douche? J Pediatr Adolesc Gynecol. 2002;15(2):83-8.
6. Markham CM, Tortolero SR, Addy RC, Baumler ER, McKirahan NK, Escobar-Chaves SL, et al. Factors associated with frequent vaginal douching among alternative school youth. J Adolesc Health. 2007;41(5):509-12.
7. Bui TC, Thai TN, Tran LT, Shete SS, Ramondetta LM, Basen-Engquist KM. Association Between Vaginal Douching and Genital Human Papillomavirus Infection Among Women in the United States. J Infect Dis. 2016;214(9):1370-5.
8. Tsai CS, Shepherd BE, Vermund SH. Does douching increase risk for sexually transmitted infections? A prospective study in high-risk adolescents. Am J Obstet Gynecol. 2009;200(1):38 e1-8.
9. Brotman RM, Klebanoff MA, Nansel TR, Andrews WW, Schwebke JR, Zhang J, et al. A longitudinal study of vaginal douching and bacterial vaginosis--a marginal structural modeling analysis. Am J Epidemiol. 2008;168(2):188-96.
10. Borgdorff H, Tsivtsivadze E, Verhelst R, Marzorati M, Jurriaans S, Ndayisaba GF, et al. Lactobacillus-dominated cervicovaginal microbiota associated with reduced HIV/STI prevalence and genital HIV viral load in African women. ISME J. 2014;8(9):1781-93.
11. Borgdorff H, van der Veer C, van Houdt R, Alberts CJ, de Vries HJ, Bruisten SM, et al. The association between ethnicity and vaginal microbiota composition in Amsterdam, the Netherlands. PLoS One. 2017;12(7):e0181135.
12. Gosmann C, Anahtar MN, Handley SA, Farcasanu M, Abu-Ali G, Bowman BA, et al. Lactobacillus-Deficient Cervicovaginal Bacterial Communities Are Associated with Increased HIV Acquisition in Young South African Women. Immunity. 2017;46(1):29-37.
13. van der Veer C, Bruisten SM, van der Helm JJ, de Vries HJ, van Houdt R. The Cervicovaginal Microbiota in Women Notified for Chlamydia trachomatis Infection: A Case-Control Study at the Sexually Transmitted Infection Outpatient Clinic in Amsterdam, The Netherlands. Clin Infect Dis. 2017;64(1):24-31.
14. Turner AN, Morrison CS, Munjoma MW, Moyo P, Chipato T, van de Wijgert JH. Vaginal practices of HIV-negative Zimbabwean women. Infect Dis Obstet Gynecol. 2010;2010.
15. Ness RB, Hillier SL, Kip KE, Richter HE, Soper DE, Stamm CA, et al. Douching, pelvic inflammatory disease, and incident gonococcal and chlamydial genital infection in a cohort of high-risk women. Am J Epidemiol. 2005;161(2):186-95.
16. Ott MA, Ofner S, Fortenberry JD. Beyond douching: use of feminine hygiene products and STI risk among young women. J Sex Med. 2009;6(5):1335-40.
17. van der Helm JJ, Bom RJ, Grunberg AW, Bruisten SM, Schim van der Loeff MF, Sabajo LO, et al. Urogenital Chlamydia trachomatis infections among ethnic groups in Paramaribo, Suriname; determinants and ethnic sexual mixing patterns. PLoS One. 2013;8(7):e68698.
18. van 't Klooster C, Haabo V, Ruysschaert S, Vossen T, van Andel TR. Herbal bathing: an analysis of variation in plant use among Saramaccan and Aucan Maroons in Suriname. J Ethnobiol Ethnomed. 2018;14(1):20.
19. van der Helm JJ, Sabajo LO, Grunberg AW, Morre SA, Speksnijder AG, de Vries HJ. Point-of-care test for detection of urogenital chlamydia in women shows low sensitivity. A performance evaluation study in two clinics in Suriname. PLoS One. 2012;7(2):e32122.
20. Bom RJ, van der Helm JJ, Bruisten SM, Grunberg AW, Sabajo LO, Schim van der Loeff MF, et al. The role of Surinamese migrants in the transmission of Chlamydia trachomatis between Paramaribo, Suriname and Amsterdam, The Netherlands. PLoS One. 2013;8(11):e77977.
21. van Melle A, Parriault MC, Basurko C, Jolivet A, Flamand C, Pigeon P, et al. Knowledge, attitudes, behaviors, and practices differences regarding HIV in populations living along the Maroni

For peer review only - http://bmjopen.bmj.com/site/about/guidelines.xhtml

river: particularities of operational interest for Amerindian and Maroon populations. AIDS Care. 2015;27(9):1112-7.
22. Heng LS, Yatsuya H, Morita S, Sakamoto J. Vaginal douching in Cambodian women: its prevalence and association with vaginal candidiasis. J Epidemiol. 2010;20(1):70-6.
23. Li J, Jiang N, Yue X, Gong X. Vaginal douching and sexually transmitted infections among female sex workers: a cross-sectional study in three provinces in China. Int J STD AIDS. 2015;26(6):420-7.
24. Carter M, Gallo M, Anderson C, Snead MC, Wiener J, Bailey A, et al. Intravaginal cleansing among women attending a sexually transmitted infection clinic in Kingston, Jamaica. West Indian Med J. 2013;62(1):56-61.
25. Hickey RJ, Zhou X, Pierson JD, Ravel J, Forney LJ. Understanding vaginal microbiome complexity from an ecological perspective. Transl Res. 2012;160(4):267-82.
26. Hutchinson KB, Kip KE, Ness RB, Gynecologic Infection Follow-Through I. Vaginal douching and development of bacterial vaginosis among women with normal and abnormal vaginal microflora. Sex Transm Dis. 2007;34(9):671-5.
27. McClelland RS, Richardson BA, Graham SM, Masese LN, Gitau R, Lavreys L, et al. A prospective study of risk factors for bacterial vaginosis in HIV-1-seronegative African women. Sex Transm Dis. 2008;35(6):617-23.
28. Veer CV, Bruisten S, Houdt RV, editors. O04.2 Effects of over-the-counter lactic acid-containing vaginal douching products on the vaginal microbiota. Sex Transm Infect; 2017.
29. Klebanoff MA, Andrews WW, Yu KF, Brotman RM, Nansel TR, Zhang J, et al. A pilot study of vaginal flora changes with randomization to cessation of douching. Sex Transm Dis. 2006;33(10):610-3.
30. Sivapalasingam S, McClelland RS, Ravel J, Ahmed A, Cleland CM, Gajer P, et al. An effective intervention to reduce intravaginal practices among HIV-1 uninfected Kenyan women. AIDS Res Hum Retroviruses. 2014;30(11):1046-54.
31. Ravel J, Gajer P, Abdo Z, Schneider GM, Koenig SS, McCulle SL, et al. Vaginal microbiome of reproductive-age women. Proc Natl Acad Sci U S A. 2011;108 Suppl 1:4680-7.
32. Fettweis JM, Brooks JP, Serrano MG, Sheth NU, Girerd PH, Edwards DJ, et al. Differences in vaginal microbiome in African American women versus women of European ancestry. Microbiology. 2014;160(Pt 10):2272-82.
33. Terborg JRH. Sexual behaviour and sexually transmitted diseases among Saramaka and Ndjuka Maroons in the hinterland of Suriname. Paramaribo: MZ Primary Health Care Suriname : ProHealth; 2001. v, 83 p. p.

For peer review only - http://bmjopen.bmj.com/site/about/guidelines.xhtml

Table 1. Epidemiological characteristics of Creole, Hindustani, Javanese, Maroon and Mixed descent women (n=1040) recruited at the Family planning clinic and STI clinic, Paramaribo, Suriname, 2009-2010.

		Creole	Hindustani	Javanese	Maroon	Mixed descent	P value
		(N=278)	(N=256)	(N=163)	(N=138)	(N=205)	
		n (%)	n (%)	n (%)	n (%)	n (%)	
**Demographic characteristics**							
Recruitment site							<0.001
	Family planning clinic	199 (71.6)	226 (88.3)	146 (89.6)	85 (61.6)	138 (67.3)	
	STI clinic	79 (28.4)	30 (11.7)	17 (10.4)	53 (38.4)	67 (32.7)	
Median age in years [IQR]		29 [24-35]	31 [26-39]	30 [25-36]	28 [23-34]	27 [23-33]	<0.001
Age in years	<25	81 (29.1)	44 (17.2)	39 (23.9)	48 (34.8)	69 (33.7)	0.001
	25-29	64 (23.0)	63 (24.6)	41 (25.2)	29 (21.0)	53 (25.9)	
	30-34	60 (21.6)	50 (19.5)	33 (20.2)	30 (21.7)	40 (19.5)	
	≥35	73 (26.3)	99 (38.7)	50 (30.7)	31 (22.5)	43 (21.0)	

For peer review only - http://bmjopen.bmj.com/site/about/guidelines.xhtml

Education							<0.001
	Low	83 (29.9)	103 (40.2)	51 (31.3)	78 (56.5)	40 (19.5)	
	Medium	147 (52.9)	130 (50.8)	89 (54.6)	41 (29.7)	109 (53.2)	
	High	43 (15.5)	21 (8.2)	21 (12.9)	9 (6.5)	52 (25.4)	
	Unknown	5 (1.8)	2 (0.8)	2 (1.2)	10 (7.2)	4 (2.0)	
**Symptoms**							
Any symptoms		204 (73.4)	181 (70.7)	120 (73.6)	117 (84.8)	140 (68.3)	0.012
Dysuria[a]		60 (21.8)	80 (31.2)	39 (23.9)	46 (33.6)	42 (20.6)	0.008
Dyspareunia** [b]		66 (24.3)	73 (29.0)	41 (25.2)	41 (31.1)	51 (24.9)	0.506
Change in fluor/vaginal discharge[c]		149 (54.0)	126 (49.2)	92 (56.4)	79 (57.7)	110 (53.7)	0.488
Irregular menstruation [d]		81 (29.3)	71 (28.1)	32 (19.9)	51 (38.1)	52 (25.7)	0.012
Abdominal pain[e]		106 (39.8)	106 (41.9)	67 (42.1)	74 (54.4)	78 (38.6)	0.041
**Vaginal hygiene**							

For peer review only - http://bmjopen.bmj.com/site/about/guidelines.xhtml

Performs vaginal hygiene		109 (39.2)	51 (19.9)	29 (17.8)	112 (81.2)	78 (38.0)	<0.001
Vaginal products used[f]	Water	35 (12.6)	28 (10.9)	17 (10.4)	29 (21.0)	31 (15.1)	0.039
	Herbs	70 (25.2)	9 (3.5)	8 (4.9)	95 (68.8)	29 (14.1)	<0.001
	Lactacyd®	9 (3.2)	5 (2.0)	1 (0.6)	2 (1.4)	14 (6.8)	0.003
	Vinegar	9 (3.2)	2 (0.8)	4 (2.5)	2 (1.4)	9 (4.4)	0.118
	Dettol®	4 (1.4)	4 (1.6)	0 (0.0)	0 (0.0)	0 (0.0)	0.105
	Other[g]	10 (3.6)	7 (2.7)	2 (1.2)	1 (0.7)	4 (2.0)	0.314
Frequency of vaginal hygiene[h] #							0.027
	Daily	36 (43.9)	11 (32.4)	7 (41.2)	53 (63.1)	25 (39.7)	
	Once a week	18 (22.0)	12 (35.3)	3 (17.6)	19 (22.6)	11 (17.5)	
	Once a month	19 (23.2)	7 (20.6)	5 (29.4)	7 (8.3)	20 (31.7)	
	Less than once a month	9 (11.0)	4 (11.8)	2 (11.8)	5 (6.0)	7 (11.1)	
Reason for vaginal use of herbs[f,i] ##	Hygiene	50 (71.4)	4 (44.4)	5 (62.5)	49 (51.6)	16 (55.2)	0.107
	Sexual	24 (34.3)	0 (0.0)	1 (12.5)	46 (48.4)	11 (37.9)	0.0150.485
	Health	11 (15.7)	0 (0.0)	1 (12.5)	16 (16.8)	2 (6.9)	-[*]
	Other	3 (4.3)	0 (0.0)	0 (0.0)	6 (6.3)	0 (0.0)	

For peer review only - http://bmjopen.bmj.com/site/about/guidelines.xhtml

Last vaginal use of herbs[j] ##							-[*]
	Today	7 (12.7)	0 (0.0)	0 (0.0)	19 (26.4)	1 (5.9)	
	Yesterday	5 (9.1)	0 (0.0)	0 (0.0)	23 (31.9)	1 (5.9)	
	Last week	20 (36.4)	0 (0.0)	2 (33.3)	15 (20.8)	4 (23.5)	
	More than a week ago	23 (41.8)	4 (100.0)	4 (66.7)	15 (20.8)	11 (64.7)	
**Sexual behaviour**							
Number of partners in the preceding month[k]							<0.001
	0	17 (6.2)	8 (3.1)	2 (1.2)	10 (7.5)	11 (5.7)	
	1	237 (87.1)	236 (92.5)	148 (91.4)	117 (87.3)	158 (82.3)	
	2	16 (5.9)	6 (2.4)	11 (6.8)	7 (5.2)	10 (5.2)	
	>2	2 (0.7)	5 (2.0)	1 (0.6)	0 (0.0)	13 (6.8)	
Condom use during sex[l]							<0.001
	Always	49 (18.0)	17 (6.7)	10 (6.2)	23 (16.8)	37 (18.0)	
	Never or inconsistent	223 (82.0)	236 (93.3)	152 (93.8)	114 (83.2)	168 (82.0)	

For peer review only - http://bmjopen.bmj.com/site/about/guidelines.xhtml

Number of partners in the preceding 12 months							<0.001
	0	8 (2.9)	12 (4.7)	5 (3.1)	11 (8.0)	11 (5.4)	
	1	208 (74.8)	209 (81.6)	123 (75.5)	82 (59.4)	127 (62.0)	
	2	35 (12.6)	27 (10.5)	20 (12.3)	33 (23.9)	35 (17.1)	
	>2	27 (9.7)	8 (3.1)	15 (9.2)	12 (8.7)	32 (15.6)	
Ethnic sexual mixing	Reported ≥ 1 sexual partner from another ethnic group	89 (32.0)	45 (17.6)	80 (49.1)	43 (31.2)	123 (60.0)	<0.001
Sex in exchange for money or goods[b]		7 (2.6)	6 (2.4)	0 (0.0)	5 (3.7)	19 (9.4)	<0.001
Chlamydia trachomatis infection ###		37 (13.3)	16 (6.2)	25 (15.3)	15 (10.9)	29 (14.1)	0.022

a: 5 missings, b: 16 missings, c: 3 missings, d: 14 missings, e:24 missings, f: multiple options could be chosen, g: other vaginal practices are products like lactacyd but from other brands; soap, anti fungal and eggs., h: 99 missings, i: 56 missings, % of those who filled in the question, j: 57 missings, k: 25 missings, l: 11 missings.
*: *p* values could not obtained due to low numbers. IQR = interquartile range.

** pain during sexual intercourse

the denominator for the percentages is the group of women who indicated they performed vaginal hygiene.

the denominator for the percentages is the group of women who indicated they performed vaginal hygiene.

as diagnosed by nucleic acid amplification test

For peer review only - http://bmjopen.bmj.com/site/about/guidelines.xhtml

Table 2. Univariable and multivariable logistic regression analyses of determinants associated with vaginal herb use among Creole and Maroon women, Paramaribo, Suriname, 2009-2010.

		Use of vaginal herbs	Univariable OR (95%CI)	*P* value	Multivariable Adjusted OR (95%CI)*	*P* value
		n/N (%)				
**Demographic characteristics**						
Ethnic groups	Creole	70/278 (25.2)	1	<0.001	1	<0.001
	Maroon	95/138 (68.8)	6.57 (4.18-10.30)		5.33 (3.26-8.71)	
Recruitment site	Family planning clinic	96/284 (33.8)	1	<0.001	1	0.005
	STI clinic	69/132 (52.3)	2.15 (1.41-3.27)		2.04 (1.24-3.36)	
Age in years				0.068		0.067
	<25	59/129 (45.7)	1.99 (1.15-3.42)		2.23 (1.17-4.27)	
	25-29	35/93 (37.6)	1.42 (0.79-2.57)		1.78 (0.88-3.57)	
	30-34	40/90 (44.4)	1.88 (1.04-3.40)		2.23 (1.13-4.42)	
	≥35	31/104 (29.8)	1		1	
Education[a]				<0.001		0.002
	Low	88/161 (54.7)	5.06 (2.38-10.79)		3.80 (1.68-8.57)	
	Medium	59/188 (31.4)	1.92 (0.90-4.09)		2.02 (0.90-4.51)	
	High	10/52 (19.2)	1		1	

For peer review only - http://bmjopen.bmj.com/site/about/guidelines.xhtml

**Sexual behaviour**				
Number partners preceding month[b]				0.106
	0	10/27 (37.0)	1	
	1	135/354 (38.1)	1.05 (0.47-2.36)	
	≥2	15/25 (60.0)	2.55 (0.83-7.80)	
Number partners preceding 12 months				0.216
	0	9/19 (47.4)	1	
	1	107/290 (36.9)	0.65 (0.26-1.65)	
	≥2	49/107 (45.8)	0.94 (0.35-2.50)	
Ethnic sexual mixing	No	115/284 (40.5)	1	0.612
	Yes	50/132 (37.9)	0.90 (0.59-1.37)	
Sex in exchange for money or goods[c]	No	155/396 (39.1)	1	0.191
	Yes	7/12 (58.3)	2.17 (0.68-6.98)	
**Symptoms**				
Any symptoms	No	24/95 (25.3)	1	0.001
	Yes	141/321 (43.9)	2.32 (1.39-3.87)	
Dysuria[d]	No	114/306 (37.3)	1	0.104
	Yes	49/106 (46.2)	1.45 (0.93-2.26)	

For peer review only - http://bmjopen.bmj.com/site/about/guidelines.xhtml

Dyspareunia (pain during sexual intercourse)[e]	No<br>Yes	113/297 (38.0)<br>47/107 (43.9)	1<br>1.28 (0.82-2.00)	0.287
Change in fluor/vaginal discharge[f]	No<br>Yes	71/185 (38.4)<br>93/228 (40.8)	1<br>1.11 (0.74-1.65)	0.619
Irregular menstruation[g]	No<br>Yes	102/278 (36.7)<br>59/132 (44.7)	1<br>1.40 (0.92-2.13)	0.122
Abdominal pain[h]	No<br>Yes	78/222 (35.1)<br>83/180 (46.1)	1<br>1.58 (1.06-2.36)	0.026

*ORs in the multivariable model are adjusted for all factors for which adjusted ORs are shown. In the final model 401 participants were included.
a:15 missings, b: 10 missings, c: 8 missings, d: 4 missings , e: 12 missings, f: 3 missings, g: 6 missings, h: 14 missings

For peer review only - http://bmjopen.bmj.com/site/about/guidelines.xhtml

Table 3. Vaginal products mentioned among female participants performing vaginal hygiene, Paramaribo, Suriname, 2009-2010.

Vaginal product (scientific names if applicable)	Maroon ethnicity (N=112)	Creole ethnicity (N=109)	Other ethnicities[a] (N=158)
Almond leaves (*Terminalia catappa*)	1	3	1
Andoya (*Campomanesia aromatica*)	6	1	1
Blaka masusa	0	1	0
Blaka uma	0	5	2
Broko pie (*Bellucia grossularioides*)	1	1	0
Dettol®	0	4	4
Djamu (*Syzygium cumini*)	9	4	0
Douchegel	0	0	1
Dram (alcohol)	0	1	0
Eggs	0	1	0
Eva products	0	1	0
Faya lobi (*Ixora coccinea)*	2	0	0
Feififingawiwiri	1	1	0
Guave (*Psidium guajava)*	3	2	0
Intimate wash products	0	3	5
Kill somebody (*Dimorphandra conjugata)*	13	4	1
Lactacyd®	2	9	20
Manjablad (Mango leaves)	4	9	3
Odany jewa	0	0	3
Paraklem	4	1	0
Pedreku	1	1	2
Pikin bë (*Vismia* Vand. spp)	7	5	14
Redikatun (*Gossypium barbadense*)	4	16	6
Suku trobi	7	3	0
Twigs	0	0	1
Umaanesi	4	1	2
Unknown leaves	13	6	6
Vagisil	0	1	0
Vinegar	2	9	15

For peer review only - http://bmjopen.bmj.com/site/about/guidelines.xhtml

Wasduku (*Clidemia capitellata*)	9	1	3
Water	29	35	76
Yarakopie	2	3	1
Yoghurt	0	0	1

a: Hindustani, Javanese and Mixed race ethnicity

For peer review only - http://bmjopen.bmj.com/site/about/guidelines.xhtml

Table 4. Univariable and multivariable logistic regression analyses of variables associated with cervicovaginal *C. trachomatis* infection among Creole and Maroon women, Paramaribo, Suriname, 2009-2010.

		NAAT positive	Univariable OR (95%CI)	*P* value	Multivariable Adjusted OR (95%CI)*	*P* value
		n/N (%)				
**Demographic characteristics**						
Ethnic group	Creole	37/278 (13.3)	1	0.479		
	Maroon	15/138 (10.9)	0.79 (0.42-1.50)			
Recruitment site	Family planning clinic	25/284 (8.8)	1	0.001	1	0.003
	STI clinic	27/132 (20.5)	2.66 (1.48-4.80)		2.59 (1.39-4.83)	
Age in years				0.001		0.002
	<25	25/129 (19.4)	4.76 (1.75-12.92)		4.37 (1.59-12.00)	
	25-29	17/93 (18.3)	4.43 (1.56-12.54)		4.96 (1.72-14.26)	
	30-34	5/90 (5.6)	1.17 (0.33-4.16)		1.20 (0.33-4.35)	
	≥35	5/104 (4.8)	1		1	

For peer review only - http://bmjopen.bmj.com/site/about/guidelines.xhtml

Education				0.444		
	Low	19/161 (11.8)	1.03 (0.39-2.73)			
	Medium	23/188 (12.2)	1.07 (0.41-2.78)			
	High	6/52 (11.5)	1			
	Unknown	4/15 (26.7)	2.79 (0.67-11.60)			
**Vaginal hygiene**						
Performed vaginal hygiene	No	21/195 (10.8)	1	0.317		
	Yes	31/221 (14.0)	1.35 (0.75-2.44)			
Herb use	No	27/251 (10.8)	1	0.187	1	0.564
	Yes	25/165 (15.2)	1.48 (0.83-2.66)		1.20 (0.65-2.22)	
Frequency of performing vaginal hygiene[b]	Daily	16/89 (18.0)	1.82 (0.90-3.68)	0.282		
	At least once a week	7/36 (19.4)	2.00 (0.78-513)			
	At least once a month	2/25 (8.0)	0.72 (0.16-3.28)			
	Less then once a month	3/14 (21.4)	2.26 (0.58-8.76)			
	Never	21/195 (10.8)	1			
**Sexual behaviour**						
Number of partners preceding month[c]	0-1	43/381 (11.3)	1	0.018		
	≥2	7/25 (28.0)	3.06 (1.21-7.74)			

For peer review only - http://bmjopen.bmj.com/site/about/guidelines.xhtml

Condom use[d]	Always<br>Never or inconsistent	11/72 (15.3)<br>41/337 (12.2)	1.30 (0.63-2.68)<br>1	0.473
Number of partners in the preceding 12 months	0-1<br>≥2	37/309 12.0)<br>15/107 (14.0)	1<br>1.20 (0.63-2.28)	0.582
Ethnic sexual mixing	Reported only sexual partners from own ethnic group	32/284 (11.3)	1	0.267
	Reported at least one sexual partner from another ethnic group	20/132 (15.2)	1.41 (0.77-2.57)	
Sex in exchange for money or goods[e]	No<br>Yes	47/396 (11.9)<br>4/12 (33.3)	1<br>3.71 (1.08-12.81)	0.038

*ORs in the multivariable model are adjusted for all factors for which adjusted ORs are shown. In the final model 416 participants were included.
Footnote: NA: not applicable.
, b: 57 missings, c: 10 missings, d: 7 missings, e: 8 missings,

For peer review only - http://bmjopen.bmj.com/site/about/guidelines.xhtml

STROBE Statement—Checklist of items that should be included in reports of ***cross-sectional studies***

	Item No	Recommendation	Reported on page #
**Title and abstract**	1	(*a*) Indicate the study's design with a commonly used term in the title or the abstract	1
		(*b*) Provide in the abstract an informative and balanced summary of what was done and what was found	2
**Introduction**			
Background/rationale	2	Explain the scientific background and rationale for the investigation being reported	4
Objectives	3	State specific objectives, including any prespecified hypotheses	4/5
**Methods**			
Study design	4	Present key elements of study design early in the paper	5
Setting	5	Describe the setting, locations, and relevant dates, including periods of recruitment, exposure, follow-up, and data collection	5
Participants	6	(*a*) Give the eligibility criteria, and the sources and methods of selection of participants	5
Variables	7	Clearly define all outcomes, exposures, predictors, potential confounders, and effect modifiers. Give diagnostic criteria, if applicable	5/6
Data sources/ measurement	8*	For each variable of interest, give sources of data and details of methods of assessment (measurement). Describe comparability of assessment methods if there is more than one group	*6*
Bias	9	Describe any efforts to address potential sources of bias	5/6
Study size	10	Explain how the study size was arrived at	Ref 19
Quantitative variables	11	Explain how quantitative variables were handled in the analyses. If applicable, describe which groupings were chosen and why	6
Statistical methods	12	(*a*) Describe all statistical methods, including those used to control for confounding	6
		(*b*) Describe any methods used to examine subgroups and interactions	6
		(*c*) Explain how missing data were addressed	15
		(*d*) If applicable, describe analytical methods taking account of sampling strategy	n.a.
		(*e*) Describe any sensitivity analyses	6
**Results**			

For peer review only - http://bmjopen.bmj.com/site/about/guidelines.xhtml

Participants	13*	(a) Report numbers of individuals at each stage of study—eg numbers potentially eligible, examined for eligibility, confirmed eligible, included in the study, completing follow-up, and analysed	6
		(b) Give reasons for non-participation at each stage	n.a.
		(c) Consider use of a flow diagram	n.a.
Descriptive data	14*	(a) Give characteristics of study participants (eg demographic, clinical, social) and information on exposures and potential confounders	6/13
		(b) Indicate number of participants with missing data for each variable of interest	15
Outcome data	15*	Report numbers of outcome events or summary measures	7/16/20
Main results	16	(*a*) Give unadjusted estimates and, if applicable, confounder-adjusted estimates and their precision (eg, 95% confidence interval). Make clear which confounders were adjusted for and why they were included	16/20
		(*b*) Report category boundaries when continuous variables were categorized	13-21
		(*c*) If relevant, consider translating estimates of relative risk into absolute risk for a meaningful time period	n.a.
Other analyses	17	Report other analyses done—eg analyses of subgroups and interactions, and sensitivity analyses	n.a.
**Discussion**			
Key results	18	Summarise key results with reference to study objectives	8
Limitations	19	Discuss limitations of the study, taking into account sources of potential bias or imprecision. Discuss both direction and magnitude of any potential bias	8
Interpretation	20	Give a cautious overall interpretation of results considering objectives, limitations, multiplicity of analyses, results from similar studies, and other relevant evidence	8/9
Generalisability	21	Discuss the generalisability (external validity) of the study results	9
**Other information**			
Funding	22	Give the source of funding and the role of the funders for the present study and, if applicable, for the original study on which the present article is based	1

*Give information separately for exposed and unexposed groups.

Note: An Explanation and Elaboration article discusses each checklist item and gives methodological background and published examples of transparent reporting. The STROBE checklist is best used in conjunction with this article (freely available on the Web sites of PLoS Medicine at http://www.plosmedicine.org/, Annals of Internal Medicine at

For peer review only

For peer review only - http://bmjopen.bmj.com/site/about/guidelines.xhtml

http://www.annals.org/, and Epidemiology at http://www.epidem.com/). Information on the STROBE Initiative is available at www.strobe-statement.org.

For peer review only

For peer review only - http://bmjopen.bmj.com/site/about/guidelines.xhtml
