# Supplementary material for: Vaginal herb use and Chlamydia trachomatis infection: cross-sectional study among women of various ethnic groups in Suriname
Source: BMJ Open. 2019 May 16;9(5):e025417. doi: 10.1136/bmjopen-2018-025417 (PMC6530446; doi:10.1136/bmjopen-2018-025417)
Supplement: Reviewer comments [file bmjopen-2018-025417.reviewer_comments.pdf]

PEER REVIEW HISTORY

BMJ Open publishes all reviews undertaken for accepted manuscripts. Reviewers are asked to complete a checklist review form (**http://bmjopen.bmj.com/site/about/resources/checklist.pdf**) and are provided with free text boxes to elaborate on their assessment. These free text comments are reproduced below.

ARTICLE DETAILS

**TITLE (PROVISIONAL)**	Vaginal herb use and Chlamydia trachomatis infection: a cross-sectional study among women of various ethnic groups in Suriname
**AUTHORS**	Van der Helm, Jannie; Schim van der Loeff, Maarten; de Vries, Esther; van der Veer, Charlotte; Grünberg, Antoon; Mans, Dennis; de Vries, H

VERSION 1 - REVIEW

**REVIEWER**	Richard Harry Asmah, DPhil<br>University of Ghana, Ghana
**REVIEW RETURNED**	23-Aug-2018

**GENERAL COMMENTS**	The study is an excellent one. Could you however, explain why high proportions of Javanese women were infected Chlamydia trachomatis even though they did not engage in vaginal practices compared to the other ethnic groups.

**REVIEWER**	Ramdas Sahienshadebie<br>Academic Medical Centre, The Netherlands
**REVIEW RETURNED**	27-Aug-2018

**GENERAL COMMENTS**	4) Methods: In the abstract under 'methods' the authors stated:<br>"Nurses collected a questionnaire and vaginal swabs for nucleic acid amplification C. trachomatis testing."<br>When I read this it is unclear for me whether the nurses interviewed the participants or whether the nurses only collected self-administered questionnaires<br>.<br>When I read the method section in the text, there also the authors do not clarify who interviewed the women.<br>The authors also insufficiently described what type of questionnaire was used: structured, semi-structured, open? How did they analyze the open question on the reasoning of women to use a certain type of herb? I would suggest to elaborate or clarify a bit.<br><br>12) About the strenghts of this study: the authors stated:<br>"This is the first study to show that vaginal herb use in Suriname is common among Maroon and Creole women (of African descent)."

	This is incorrect to my knowledge. Earlier studies reported already on the use of vaginal herbs as being common among Maroon and Creole women in Suriname (and the Netherlands):<br>- Van Andel, T., de Korte, S, Koopmans, D. , Behari-Ramdas, J. & S. Ruysschaert (2008) 'Wasi Ondrosei' Het gebruik van vaginale stoombaden, OSO, Tijdschrift voor Surinamistiek en het Caraibisch gebied 27(1):52-71<br>-Terborg, J (2001) Sexual behaviour and sexually transmitted diseases among the Saramaka and the Ndjuka Maroons in the hinterland of Suriname. Paramaribo: ProHealth & Primary Health Care Suriname.<br><br>The authors also used these, or at least one of these articles, so what do they mean with their statement?

**REVIEWER**	Lisa Vallely<br>Kirby Institute UNSW Sydney Sydney, NSW Australia
**REVIEW RETURNED**	30-Aug-2018

**GENERAL COMMENTS**	Dear Authors<br>This was an intersting paper to review. I think the introduction and discussion are well written. Howevere I have some concerns ove rthe results section which was difficult to understand. I hope the tracked changes in the pdf copy may help you to present the results more clearly.<br><br>The reviewer provided a marked copy with additional comments. Please contact the publisher for full details.

VERSION 1 – AUTHOR RESPONSE

Reviewer: 1

The study is an excellent one. Could you however, explain why high proportions of Javanese women were infected Chlamydia trachomatis even though they did not engage in vaginal practices compared to the other ethnic groups.

Response: Thank you for your compliments. Your comment on the higher C. trachomatis prevalence among Javanese women is a very important one. We previously described that apart from a higher prevalence among Javanese women, also disassortative mixing (having sexual partners outside of one's ethnic group) was higher among Javanese women, but this was not associated with C. trachomatis positivity (van der Helm JJ, Bom RJM, Gru¨nberg AW, Bruisten SM, Schim van der Loeff MF, et al. (2013) Urogenital Chlamydia trachomatis Infections among Ethnic Groups in Paramaribo, Suriname; Determinants and Ethnic Sexual Mixing Patterns. PLoS ONE 8(7): e68698. doi:10.1371/journal.pone.0068698). Here we found no association with the use of vaginal herb use. So, the increased prevalence among Javanese women remains unexplained.

Reviewer: 2

4) Methods: In the abstract under 'methods' the authors stated: "Nurses collected a questionnaire and vaginal swabs for nucleic acid amplification C. trachomatis testing."

When I read this it is unclear for me whether the nurses interviewed the participants or whether the nurses only collected self-administered questionnaires

When I read the method section in the text, there also the authors do not clarify who interviewed the women.

The authors also insufficiently described what type of questionnaire was used: structured, semi-structured, open? How did they analyze the open question on the reasoning of women to use a certain type of herb? I would suggest to elaborate or clarify a bit.

We have clarified the way the questionnaires were filled out in the method section under paragraph “vaginal hygiene practices”: “Using a structured questionnaire, nurses asked women in detail about the use of products for vaginal practices such as…” and “Women who used vaginal herbs were asked about their most recent application, and the reasons for use (hygiene, sexual pleasure, health or other). Items under reason other that fitted the 3 former reasons were regrouped accordingly.”

12) About the strenghts of this study: the authors stated:

“This is the first study to show that vaginal herb use in Suriname is common among Maroon and Creole women (of African descent).”

This is incorrect to my knowledge. Earlier studies reported already on the use of vaginal herbs as being common among Maroon and Creole women in Suriname (and the Netherlands):

- Van Andel, T., de Korte, S, Koopmans, D. , Behari-Ramdas, J. & S. Ruysschaert (2008) ‘Wasi Ondrosei’ Het gebruik van vaginale stoombaden, OSO, Tijdschrift voor Surinamistiek en het Caraibisch gebied 27(1):52-71

-Terborg, J (2001) Sexual behaviour and sexually transmitted diseases among the Saramaka and the Ndjuka Maroons in the hinterland of Suriname. Paramaribo: ProHealth & Primary Health Care Suriname.

The authors also used these, or at least one of these articles, so what do they mean with their statement?

Thank you for pointing on this statement. You are correct that this is not the first study describing the use of vaginal herbs among ethnic groups in Suriname. We have nuanced our statement as follows: “This is the first study into the relationship between vaginal herb use and a sexually transmitted infection in Suriname.”

Reviewer: 3

Dear Authors

This was an intersting paper to review. I think the introduction and discussion are well written. Howevere I have some concerns ove rthe results section which was difficult to understand. I hope the tracked changes in the pdf copy may help you to present the results more clearly.

Thank you for your helpful suggestions that have improved our manuscript. Below, please find a point by point rebuttal to your comments.

Page 4 Line 4: “Depending on cultural habits” I think you could delete these first few words, don't add anything.

We added this phrase to place our study objective, vaginal herb use among different ethnic groups, in perspective.

Page 4 Line 34-35: A reference is asked for the claim that Suriname is ethnically and culturally among the most varied in the world.

We have rephrased this sentence into: “The population of approximately 570,000 is characterized by it’s ethnical and cultural diversity.”

Page 4 Line 45: According to your suggestion we rephrased the sentence to: “Among these ethnic groups, Afro-Surinamese women (Creoles and Maroons) in particular…”

Page 4 Line 54: We removed the word “specific” here.

Page 5 Line 4: We added 2 references (3 and 18) here.

Page 5 Line 6: We added a specification of the setting of the study: “women visiting an STI outpatient clinic and a family planning (FP) clinic”.

Page 5 Line 35: We clarified: “participation within the CUSTEPA study.”

Page 5 Line 40: On patient and public involvement, we only consulted representatives of the Maroon population on the content of this study. We did recruit participants with other backgrounds though. The reason we specifically consulted the Maroon representatives is because of the specific sensitivity existing around the use of vaginal herb use within this community.

Page 6 Line 8: We added a reference here (19)

Page 6 Lines 48-53 were rephrased: “We initially included 1,093 women who self-identified with the following ethnic backgrounds: 17 (1.6%) Caucasian, 12 (1.1%) Chinese, 278 (25.4%) Creole, 256 (23.4%) Hindustani, 19 (1.7%) Indigenous, 163 (14.9%) Javanese, 138 (12.6%) Maroon, 205 (18.8%) Mixed descent, and 5 unknown (0.5%). Due to the small numbers we excluded Caucasian, Chinese, Indigenous women, and women with unknown background from further analysis. As a result, the study population included 1,040 women of either Creole, Hindustani, Javanese, Maroon, or Mixed descent (Table 1).”

Page 7 Line 14-22 were rephrased: “Vaginal hygiene

Table 1 shows the ways in which vaginal hygiene was performed within the study population. Vaginal hygiene was most common in Maroon women (81.2%) followed by Creole (39.2%) and Mixed descent women (38.0%). Between 10.4% and 21.0% of women used tap water for vaginal cleansing, with the largest proportion in Maroon (21.0%) and Mixed descent women (15.1%). Lactacyd®, vinegar, and other substances such as Dettol® were used less often.

In those women that performed vaginal hygiene, daily use was reported by Maroons (63.1%), Creoles (43.9%), Javanese (41.2%), Mixed descent (39.7%), and Hindustani (32.4%).”

Page 7 Lines 38-45 were rephrased, as suggested.

Page 7 Lines 45-: All plant names were placed between brackets.

Page 8 Lines 10-18 were rephrased as suggested.

VERSION 2 – REVIEW

**REVIEWER**	Richard Harry Asmah, DPhil<br>University of Ghana, Ghana
**REVIEW RETURNED**	21-Dec-2018

**GENERAL COMMENTS**	The paper is an excellent one and you addressed the question I asked.

**REVIEWER**	Sahienshadebie Ramdas<br>Academic Medical Center, University of Amsterdam, The Netherlands
**REVIEW RETURNED**	02-Jan-2019

**GENERAL COMMENTS**	I complement the authors with the revised version of their article. It is an important topic presented in a digestible and structured way. It is well written and reads fluently. All areas needing clarity were addressed by the authors. They provided sufficient information, especially in the methods section, elaborated parts where necessary and altered sentences wherever needed or as pointed out by the reviewers. I have no further comments.

**REVIEWER**	Lisa Vallely<br>Kirby Institute UNSW Australia
**REVIEW RETURNED**	17-Feb-2019

**GENERAL COMMENTS**	My aplogies for the late response to review this paper.
